# Deciphering transcriptome alterations in bone marrow hematopoiesis at single-cell resolution in immune thrombocytopenia

**DOI:** 10.1038/s41392-022-01167-9

**Published:** 2022-10-07

**Authors:** Yan Liu, Xinyi Zuo, Peng Chen, Xiang Hu, Zi Sheng, Anli Liu, Qiang Liu, Shaoqiu Leng, Xiaoyu Zhang, Xin Li, Limei Wang, Qi Feng, Chaoyang Li, Ming Hou, Chong Chu, Shihui Ma, Shuwen Wang, Jun Peng

**Affiliations:** 1 Department of Hematology, Qilu Hospital, Cheeloo College of Medicine, Shandong University, Jinan, 250012 China; 2 State Key Laboratory of Experimental Hematology, National Clinical Research Center for Blood Diseases, Haihe Laboratory of Cell Ecosystem, Institute of Hematology & Blood Diseases Hospital, Chinese Academy of Medical Sciences & Peking Union Medical College, Tianjin, 300020 China; 3 Department of Hematology, the Affiliated Hospital of Qingdao University, Qingdao, 266000 China; 4 Advanced Medical Research Institute, Shandong University, Jinan, 250012 China; 5 Shangdong Key Laboratory of Immunochematology, Qilu Hospital, Cheeloo College of Medicine, Shandong University, Jinan, 250012 China; 6 Key Laboratory of Cardiovascular Remodeling and Function Research, Chinese Ministry of Education and Chinese Ministry of Health, Qilu Hospital, Cheeloo College of Medicine, Shandong University, Jinan, 250012 China; 7 Department of Biomedical Informatics, Harvard Medical School, Boston, 02115 MA USA

**Keywords:** Haematological diseases, Immunopathogenesis, Immunological disorders, Haematopoietic stem cells

## Abstract

Immune thrombocytopenia (ITP) is an autoimmune disorder, in which megakaryocyte dysfunction caused by an autoimmune reaction can lead to thrombocytopenia, although the underlying mechanisms remain unclear. Here, we performed single-cell transcriptome profiling of bone marrow CD34^+^ hematopoietic stem and progenitor cells (HSPCs) to determine defects in megakaryopoiesis in ITP. Gene expression, cell-cell interactions, and transcriptional regulatory networks varied in HSPCs of ITP, particularly in immune cell progenitors. Differentially expressed gene (DEG) analysis indicated that there was an impaired megakaryopoiesis of ITP. Flow cytometry confirmed that the number of CD9^+^ and HES1^+^ cells from Lin^−^CD34^+^CD45RA^−^ HSPCs decreased in ITP. Liquid culture assays demonstrated that CD9^+^Lin^−^CD34^+^CD45RA^−^ HSPCs tended to differentiate into megakaryocytes; however, this tendency was not observed in ITP patients and more erythrocytes were produced. The percentage of megakaryocytes differentiated from CD9^+^Lin^−^CD34^+^CD45RA^−^ HSPCs was 3-fold higher than that of the CD9^−^ counterparts from healthy controls (HCs), whereas, in ITP patients, the percentage decreased to only 1/4th of that in the HCs and was comparable to that from the CD9^−^ HSPCs. Additionally, when co-cultured with pre-B cells from ITP patients, the differentiation of CD9^+^Lin^−^CD34^+^CD45RA^−^ HSPCs toward the megakaryopoietic lineage was impaired. Further analysis revealed that megakaryocytic progenitors (MkP) can be divided into seven subclusters with different gene expression patterns and functions. The ITP-associated DEGs were MkP subtype-specific, with most DEGs concentrated in the subcluster possessing dual functions of immunomodulation and platelet generation. This study comprehensively dissects defective hematopoiesis and provides novel insights regarding the pathogenesis of ITP.

## Introduction

Immune thrombocytopenia (ITP) is an acquired autoimmune disorder characterized by reduction in platelet count and increase in the risk of bleeding.^1^ Its pathogenesis has been extensively studied, with immune-mediated increase in destruction and decrease in the production of platelets as the accepted mechanisms.^2^ Platelet production is a complex biological process that involves hematopoietic stem cell commitment to the megakaryocytic lineage, megakaryocyte maturation, and platelet release.^3,4^ Previously, our group and others have demonstrated that antiplatelet autoantibodies, bone marrow (BM) CD8^+^ T cells, and tumor necrosis factor-related apoptosis-inducing ligand in BM plasma and megakaryocytes impair megakaryopoiesis in ITP.^5–10^ Recent studies have reported selective activations of hematopoietic stem and progenitor cells (HSPCs) in a murine model of ITP or acute thrombocytopenia.^11,12^ However, whether and how hematopoietic differentiation contributes to the pathogenesis of ITP in humans remain unclear.

On the one hand, decrease in platelet count and depletion of immune cells activated hematopoiesis,^13–16^ while on the other hand, HSPCs themselves could be targets of autoimmune attack because of the target antigens present on both platelets and the more immature hematopoietic progenitors.^17–19^ In addition, the cellular crosstalk among different subclusters of HSPCs might play critical roles in inducing defective megakaryopoiesis. However, comprehensive documentation of the underlying molecular interactions in HSPCs in ITP remains an open area of investigation.

We hypothesized that HSPCs participated in the defective megakaryopoiesis in ITP patients. Here, we investigated and compared the transcriptomes of CD34^+^ HSPCs in ITP and healthy controls (HC) using single-cell RNA-seq (scRNA-seq) to reveal the overall transcriptome alterations in HSPCs in ITP. Our results provide new insights regarding the cellular and molecular basis of ITP pathogenesis.

## Results

### Single-cell transcriptomes of BM CD34^+^ HSPCs from ITP patients and HCs

**Fig. 1:**
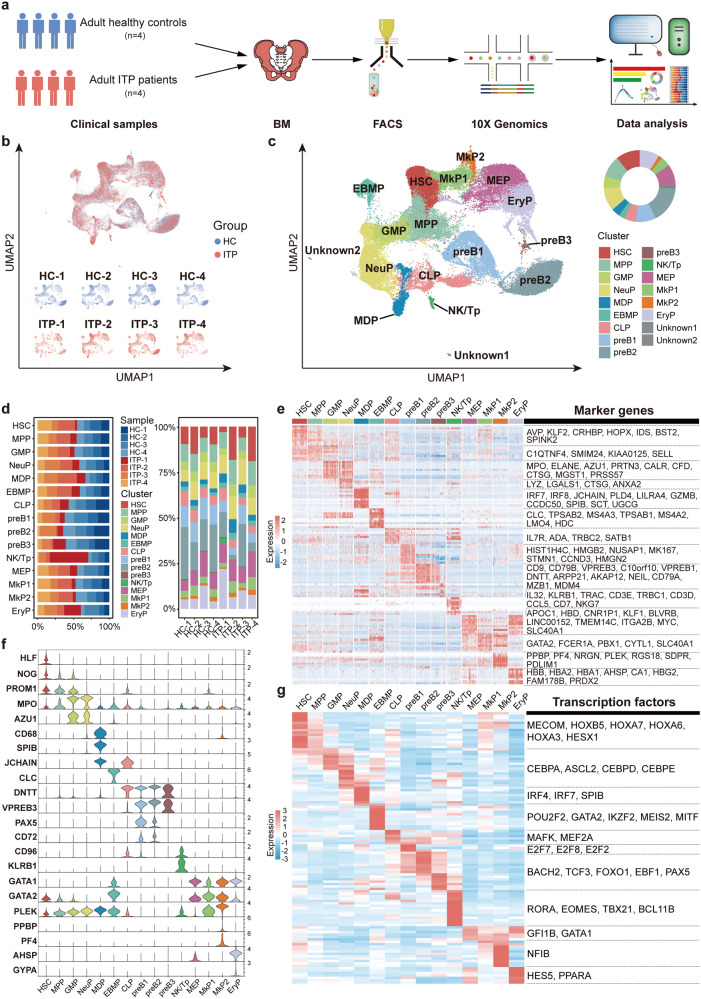
Identifying HSPCs in ITP and HC bone marrows. **a** Schematic overview. Related to Figs. 1, 2, 4, 8, and 9. **b** UMAP plot showing cell contribution by samples. **c** Cell clusters were visualized using UMAP. Colors indicate cell types. Each dot represents one cell. HSC hematopoietic stem cells, MPP multipotent progenitors, GMP granulocyte and monocyte progenitors, NeuP neutrophil progenitors, MDP monocyte-dendritic-cell progenitors, EBMP eosinophil-basophil-mast-cell progenitors, CLP common lymphoid progenitors, pre-B pre-B cells, NK/Tp natural killer/T-cell progenitors, MEP megakaryocyte-erythroid progenitors, MkP megakaryocytic progenitors, EryP erythroid progenitors. Two unknown clusters could not be identified. Both were irrelevant to this study and were not analyzed later. **d** Stacked barplots show the percentage of sample contributions per annotated cell type (left) and the percentage of annotated cell type contributions per sample (right). **e** Heat map showing the scaled expression of top 10 marker genes in each cell cluster. Vital marker genes are highlighted on the right. **f** Violin plots showing the expression of specific marker genes in each cell cluster. Colors represent the cell clusters indicated in **c**. **g** Top 10 differentially expressed transcription factors (TFs) in each cell cluster. The vital TFs related to differentiation are listed on the right

To investigate the hematopoietic transcriptional landscape and determine the alterations in ITP, we performed scRNA-seq of BM CD34^+^ HSPCs from four ITP patients and four healthy donors on the 10X Chromium platform (Fig. 1a and Supplemental Table 1). The baseline characteristics of the eight participants were summarized in Table 1.

**Table 1:** Baseline characteristics of participants enrolled for scRNA-seq

	ITP-1	ITP-2	ITP-3	ITP-4	HC-1	HC-2	HC-3	HC-4
Group	ITP	ITP	ITP	ITP	HC	HC	HC	HC
Sex	F	M	F	M	M	M	F	F
Age (year)	23	19	46	54	48	40	35	40
Treatment	--	--	--	--	--	--	--	--
Course of disease (day)	7	14	32	35	--	--	--	--
Platelet count in PB (10^9^ per L)	5	18	56	41	212	220	277	196
Serum antiplatelet antibodies (MAIPA)	+, a	+, a, b	--	+, a	--	--	--	--
Megakaryocyte count in BM (per slide)	175	155	310	225	56	22	85	32
Proportion of CD34^+^ cells in BMNCs (%)	0.70	0.65	0.91	0.58	0.86	0.79	0.48	0.75

*a* GPIIb/IIIa, *b* GPIb/IX, *BMNCs* bone marrow nuclear cells, *F* female, *HC* healthy controls, *ITP* immune thrombocytopenia, *M* male, *MAIPA* monoclonal antibody immobilization of platelet antigens, *PB* peripheral blood

After quality control and batch effect correction, 56,312 (ITP, *n* = 28,507; HC, *n* = 27,805) single-cell profiles were included in the downstream analyses (Supplemental Fig. 1a, b). Dimensional reduction using uniform manifold approximation and projection (UMAP) showed the effective integration of the datasets from different samples (Fig. 1b). Considering that the cell cycle machinery is related to the self-renewal and differentiation potential of HSPCs,^20^ we did not perform cell cycle regression (Supplemental Fig. 1c). We manually annotated the cell clusters into 17 different cell types (Fig. 1c, d and Supplemental Fig. 1b, d–f) with distinct gene expression patterns (Fig. 1e–g and Supplemental Table 2). These populations included hematopoietic stem cells (HSC), multipotent progenitors (MPP), granulocyte-macrophage progenitors (GMP), neutrophil progenitors (NeuP), monocyte-dendritic-cell progenitors (MDP), eosinophil-basophil-mast-cell progenitors (EBMP), common lymphoid progenitors (CLP), three pre-B cell populations (preB1, preB2, and preB3), natural killer/T-cell progenitors (NK/Tp), megakaryocyte-erythroid progenitors (MEP), two subpopulations of megakaryocytic progenitors (MkP1 and MkP2), erythroid progenitors (EryP), and two unknown clusters (could not be identified).

**Fig. 2:**
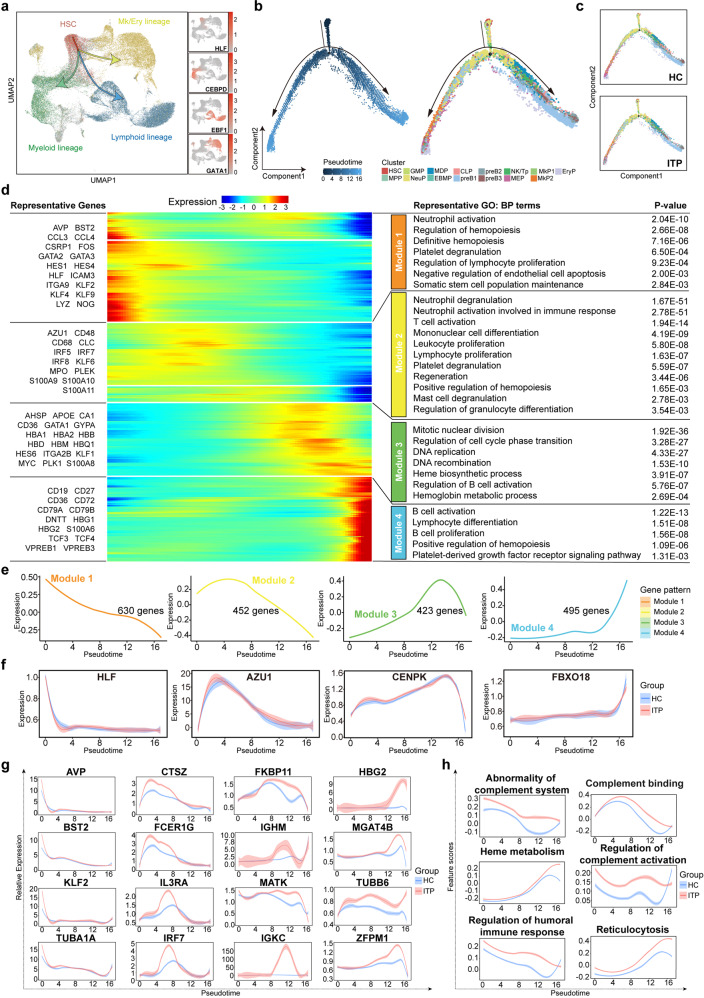
Analysis of HSPC transition states in ITP and HC samples. **a** UMAP plots displaying the expression of known marker genes (*HLF, EBF1, CEBPD*, and *GATA1*) during hematopoietic development. Arrows indicate the main directions of differentiation, inferred from the analysis of typical marker genes. **b** Pseudotime-ordered analysis of HSPCs from the ITP and HC samples. Colors represent the cell clusters indicated in Fig. 1c. **c** 2D graph of the pseudotime-ordered HSPCs from HC (top) and ITP (bottom) samples. **d** Heat map showing dynamic changes in gene expression along the pseudotime (cataloged hierarchically into four gene modules). Adjusted *p* value < 0.05 was considered statistically significant for Gene Ontology (GO) enrichment analysis. **e** Loess-smoothed curves fitted to the *z* scored averaged expression of genes in modules 1–4 along the pseudotime trajectory. **f** Dynamic expression of representative genes in each module along the pseudotime trajectory. **g** Two-dimensional plots showing the dynamic expression of significantly enhanced genes in ITP compared with HC along the pseudotime. A log-transformed fold change value greater than 0.25, the minimum percentage >0.25, and adjusted *p* value < 0.05 were used to define significantly upregulated genes. **h** Two-dimensional plots showing the dynamic expression of scores for abnormality of complement system, complement binding, heme metabolism, regulation of complement activation, regulation of humoral immune response, and reticulocytosis along with the pseudotime in ITP (red) and HC (blue) groups. The values of the *y* axis are the calculated GSVA scores. Pathways are selected from the GSEA enrichment results in ITP (NES > 1, NOM *p* val < 0.05, and FDR *q* val < 0.25)

On the basis of the expression of four well-known genes (*HLF, EBF1, CEBPD*, and *GATA1*)^21–25^ and other canonical maker genes associated with hematopoietic development, the cells were divided into four continuous parts, indicating three main directions of fdifferentiation (Fig. 2a and Supplemental Fig. 1g). We next used Monocle2^26,27^ to order these annotated cells along a continuous pseudotime trajectory and visualized cell types by cluster (Fig. 2b and Supplemental Fig. 2a). This analysis showed that the HSC was at the beginning of the trajectory path, whereas EryP and pre-B were at the terminal state, consistent with the branched expression analysis modeling (BEAM) (Supplemental Fig. 2b, c).

We further analyzed the trajectories in ITP and HC samples separately (Fig. 2c) and obtained similar transition trajectories. On the basis of the transcriptional changes associated with transitional states, four different gene expression modules were identified (modules 1–4) (Fig. 2d–f). Genes of module 1 were downregulated along the pseudotime, showing enrichment of Gene Ontology (GO) terms related to regulation of hematopoiesis, definitive hematopoiesis, somatic stem cell population maintenance, and platelet degranulation. Module 2 and 3 consisted of genes with highest expression levels in the middle of transition, with annotations of immune cell proliferation and differentiation, platelet degranulation, and heme synthesis. Module 4 represented genes upregulated at the termination, matching the phenotypes of pre-B cells. Unexpectedly, module 4 was also associated with positive regulation of hematopoiesis and platelet-related signaling pathway, suggesting that terminal state cells such as pre-B cells might affect hematopoiesis and even megakaryopoiesis.

We assessed the enhanced genes of ITP compared with HC along the pseudotime (Fig. 2g). We observed upregulation of *BST2, KLF2*, and *TUBA1A* in ITP versus HC during the initial process of pseudotime, which participated in various aspects of cellular growth and development.^28–30^ In the middle of transition, many genes involved in immunomodulatory functions, such as *CTSZ, FCER1G, IL3RA, IRF7, IGHM*, and *IGKC*, were upregulated in ITP. The elevated expression of *ZFPM1* and *HBG2* at the end of pseudotime, which play essential roles in erythroid and megakaryocytic cell differentiation,^31,32^ was indicative of the involvement of specific biological and cellular processes during the terminal stage of hematopoiesis in ITP. In addition, we calculated gene set variation analysis (GSVA) scores of pathways on the scale of pseudotime. The results showed higher scores for adaptive immunity and heme metabolism in ITP (Fig. 2h).

**Fig. 3:**
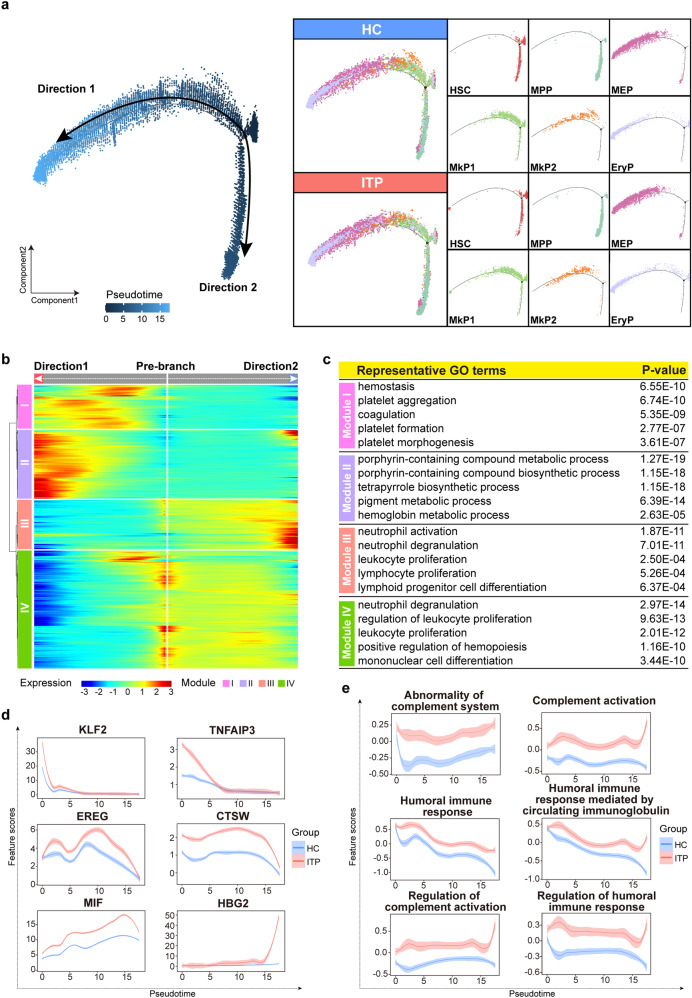
Transition state analysis of Mk/Ery lineages in ITP and HC samples. **a** Pseudotime-ordered analysis of HSC, MPP, MEP, MkP1, MkP2, and EryP populations from all ITP and HC samples. 2D graph of each cluster from HC (top) and ITP (bottom) samples are shown. **b** BEAM heat map depicting the expression of the branch-dependent genes over pseudotime. Genes are clustered to four modules based on expression patterns across pseudotime. The branch point shown in the middle of heat map is the beginning of pseudotime. Both sides of heat map are the ends of pseudotime. Color bar indicates the relative expression level. Directon1 matches the upper branch and Directon2 matches the lower branch as shown in Fig. 3a. **c** Representative GO: BP terms of each module. Adjusted *p* value < 0.05 was considered statistically significant for GO enrichment analysis. **d** Two-dimensional plots showing the dynamic expression of significantly enhanced genes in ITP along the pseudotime. **e** Two-dimensional plots showing the dynamic expression of scores for abnormality of complement system, complement activation, humoral immune response, humoral immune response mediated by circulating immunoglobulin, regulation of complement activation, and regulation of humoral immune response along with the pseudotime in ITP (red) and HC (blue) groups. The values of the *y* axis are the calculated GSVA scores. Pathways are selected from the GSEA enrichment results in ITP (NES > 1, NOM *p* val < 0.05, and FDR *q* val < 0.25)

To understand the cell transitions in megakaryocyte and erythrocyte (Mk/Ery) lineages, we next conducted a trajectory analysis of six relevant populations—HSC, MPP, MEP, MkP1, MkP2, and EryP (Fig. 3a). The results showed two main branches. MEP, MkP1, MkP2, and EryP were mainly present along the “direction 1” branch, whereas MPP was concentrated mainly along the “direction 2” branch (Fig. 3a). Notably, in the early stage of the “direction 1” branch, ITP exhibited a different distribution from HC, with sparser MkP2 and denser EryP (Fig. 3a). The BEAM and pseudotime heat maps revealed gene expression dynamics along directions and pseudotime, respectively (Fig. 3b and Supplemental Fig. 3a). The expression of genes associated with platelet formation was elevated earlier than that of heme synthesis-related genes, which was in agreement with the cell trajectory (Fig. 3c and Supplemental Fig. 3). As expected, immunomodulatory-related genes and pathways were increased in ITP (Fig. 3d, e), consistent with the results of the pseudotime analysis using all lineage cells.

Taken together, we concluded that HSCs differentiated towards the Mk lineage early during cell fate decision-making in both ITP and HC, however, the detailed distributions of Mk/Ery-lineage cells along the pseudotime were not entirely consistent, implying that the differentiation potential of HSPCs in ITP might be affected. Furthermore, the immune and transcriptional states differed considerably between ITP and HC, suggesting that immunological interactions within HSPCs should be considered for understanding the pathogenesis of ITP.

### Transcriptional changes in BM CD34^+^ HSPCs in ITP

**Fig. 4:**
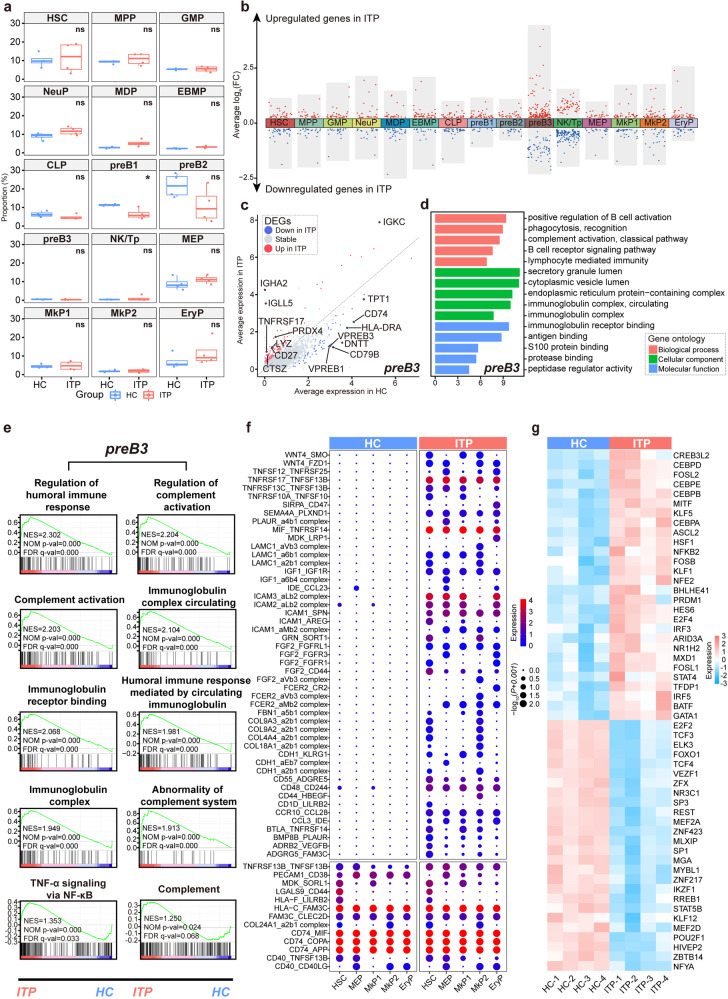
Cluster-associated alterations in HSPCs of ITP patients. **a** Boxplot showing the fraction of each HSPC cluster in ITP (blue) and HC (red) samples. The *p* values were calculated using two-tailed Student’s *t* test; *p < 0.05. **b** Differential gene expression (DGE) analysis showing up- (red) and down- (blue) regulated genes in ITP across the 15 HSPC clusters. A log-transformed fold change absolute value greater than 0.25, the minimum percentage >0.25, and adjusted *p* value <0.05 were used to define significantly differential expression genes (DEGs) in each cluster. **c** Correlation between ITP and HC transcriptomes in preB3. Each axis represents the mean expression level in the HSPC subset and each point represent a single gene. Red points represent significantly upregulated genes in ITP, blue points represent significantly downregulated genes in ITP, and gray points represent non-DEGs. A log-transformed fold change absolute value >0.25, the minimum percentage >0.25, and adjusted *p* value < 0.05 were used to define significance. **d** Representative GO: BP terms were relatively enriched in preB3 from ITP versus HC. **e** GSEA plots showing pathways enriched in preB3 from ITP versus HC. NES normalized enrichment score, FDR false discovery rate. **f** Bubble heat map of ligand-receptor interactions between preB3 and Mk/Ery-lineage cells. Interaction pairs with ITP (*p* < 0.05) were selected. ITP and HC are presented separately. Dot size indicates logarithmic transformed *p* values (permutation test). Color indicates the scaled mean expression levels of ligand and receptor molecules in the corresponding cell subpopulations. The upper panels represent interaction pairs specifically in ITP (*p* ≥ 0.05 in HC). The lower panel represents interaction pairs specific for both ITP and HC (*p* < 0.05 in HC). See also Supplemental Fig. 10. **g** Heat map of the area under the curve (AUC) scores of TF motifs estimated per sample in preB3 using SCENIC. A log-transformed fold change value >0.25, and adjusted *p* value < 0.05 were used to define significantly differential expression TFs. Significant TF motifs shared by at least two ITP samples would be selected for visualization. See also Supplemental Fig. 11

As HSPC clusters varied among samples, we compared a fraction of each cluster between ITP and HC and found that the proportion of preB1 differed significantly (Fig. 4a). We then assessed transcriptome alterations in each HSPC subset of ITP, yielding 1166 differentially expressed genes (DEGs) in total (Fig. 4b and Supplemental Table 3). Among these DEGs, the expression of 33.28% was altered in preB3 and NK/Tp. These results suggested that immune cells play an important role in the pathogenesis of ITP.

We next delineated the transcriptional changes of the three populations—preB1, preB3, and NK/Tp in ITP. In particular, genes associated with immune regulation or immune response were upregulated in these three clusters from ITP, such as *IGKC, CTSZ*, and *LYZ* in preB3, *IGKC, LYZ* and *PTPRC* in preB1, and *IL32, CD48*, and *B2M* in NK/Tp (Fig. 4c and Supplemental Fig. 4a, b). Consistent with these observations, we observed GO terms related to immune activation to be enriched in ITP (Fig. 4d and Supplemental Fig. 4a, b). Gene set enrichment analysis (GSEA) also revealed the enhancement of typical immune pathways in ITP (Fig. 4e and Supplemental Fig. 4c, d).

Based on the pseudotime trajectory, we defined HSCs, MkP1, MkP2, MEP, and EryP as Mk/Ery-lineage cells. To understand the molecular basis of autoimmunity in ITP, we used CellPhoneDB to identify potential ligand-receptor pairs between immune cell progenitors and Mk/Ery-lineage cells (Fig. 4f, Supplemental Fig. 4e, g and Supplemental Table 4). After analysis, 62 significant ligand-receptor interactions of preB3 were identified in ITP (Fig. 4f and Supplemental Table 4), which were more than that in HC. Among these predicted molecular interactions, TNFSF/TNFRSF, whose overactivation had been reported to be associated with the pathogenesis of various autoimmune diseases, was significantly activated in ITP.^33^ In addition, chemokine signaling (CCL3_IDE, CCR10_CCL28, and IDE_CCL23) and integrin-mediated cell adhesion and migration (e.g., COL9A2_a2b1 complex and ICAM2_aLb2 complex) were enhanced in ITP. These results suggested that immune dysfunctions in preB3 might be associated with defective megakaryopoiesis and thrombocytopenia in ITP. We also identified potential ligand-receptor pairs between other immune cell progenitors and Mk/Ery-lineage cells, whose ITP-association was not as obvious as that in preB3, suggesting preB3 play crucial role in ITP autoimmune response (Supplemental Fig. 4i).

We further identified several differentially expressed transcription factors (TFs) in preB3, preB1, and NK/Tp between ITP and HC (Fig. 4g and Supplemental Fig. 4f, h). TFs, including *CEBPB*, *IRF3*, *NFKB2*, and *PRDM1*, regulated immune and inflammatory responses and displayed enhanced activity in ITP, consistent with the results of gene expression data analysis. In addition, the expression of *BHLHE41* and *ARID3A*, which were the upregulated TFs, allowed mature B cell generation and maintenance with self-renewal;^34^ thus, they could indirectly exacerbate the B cell-mediated autoimmune response in ITP. Collectively, our analyses suggested that immune-associated progenitors, especially preB3, might participate in ITP pathogenesis via an immune response. The unique cell-cell interactions and dysregulation of transcriptional regulatory networks may play important roles in this process.

### *HES1* and *CD9* were downregulated in ITP patients

**Fig. 5:**
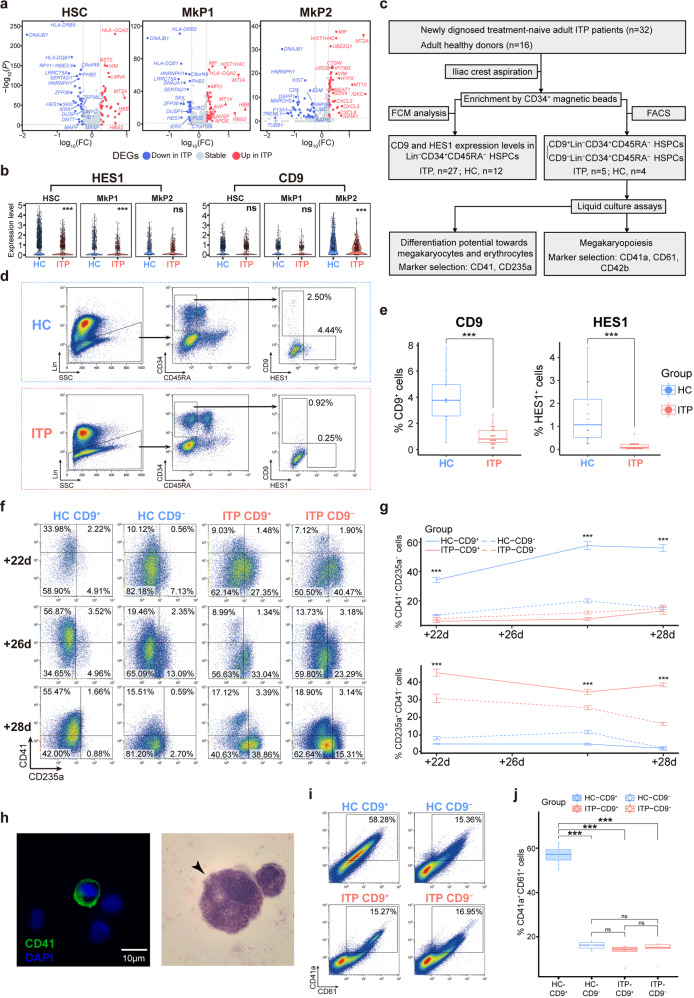
Further investigation of *HES1* and *CD9* in BM. **a** Volcano plots highlighting significant differences in gene expression between ITP and HC in HSC (left), MkP1 (middle), and MkP2 (right). Red points represent significantly upregulated genes, blue points represent significantly downregulated genes, and gray points represent non-DEGs. Genes with an adjusted *p* value < 0.05, log-transformed fold change absolute value >0.25, and minimum percentage >0.25 were considered as differentially expressed genes. **b** Violin plot representing the expression levels of *HES1* and *CD9* in HSC, MkP1, and MkP2. ***adjusted *p* value < 0.001. ns, not significant. **c** Flowchart. Related to Fig. 5d–j. **d** Expression of HES1 and CD9 in Lin^−^CD34^+^CD45RA^−^ HSPCs of ITP and HC samples. **e** Box plots showing the proportion of CD9 (left) and HES1 (right) in Lin^−^CD34^+^ CD45RA^−^ HSPCs of ITP (*n* = 27) and HC (*n* = 12) samples. ***p <0.001 by two-tailed Student’s *t* test. **f** Expression of CD41 and CD235a in cultures of CD9^+^Lin^−^CD34^+^CD45RA^−^ HSPCs and CD9^−^Lin^−^CD34^+^CD45RA^−^ HSPCs on the indicated days. Cells varied in size and granularity at different culture time-points, thus the voltages for flow cytometric analysis were adjusted accordingly, resulting in inconsistent thresholds. **g** Proportion of CD41^+^CD235a^−^ and CD235a^+^CD41^−^ cells on days 22, 26, and 28 after culturing flow-sorted CD9^+^Lin^−^CD34^+^CD45RA^−^ HSPCs and CD9^−^Lin^−^CD34^+^CD45RA^−^ HSPCs. Error bars, mean ± S.E. Data were subjected to variable transformation (arcsine square root transformed) and analyzed using one-way ANOVA. ITP, *n* = 5; HC, *n* = 4. ***p < 0.001. **h** Representative immunofluorescence microscopy images (left panel) showing the morphology of megakaryocytes after culture. Scale bar, 10 μm. Representative light microscopy images of Wright–Giemsa-stained cytospins (right panel) showing the morphology of megakaryocytes after culture. **i** The expression of CD41a, and CD61 in cultures of CD9^+^Lin^−^CD34^+^CD45RA^−^ HSPCs and CD9^−^Lin^−^CD34^+^CD45RA^−^ HSPCs on day 28. **j** Box plots showing the proportion of CD41a^+^CD61^+^ cells in cultures of CD9^+^Lin^−^CD34^+^CD45RA^−^ HSPCs and CD9^−^Lin^−^CD34^+^CD45RA^−^ HSPCs flow^-^sorted from ITP and HC BM samples. Data were subjected to variable transformation (arcsine square root transformed) and analyzed using one-way ANOVA with Scheffe’s post hoc test. ITP, *n* = 5; HC, *n* = 4. ***p < 0.001. ns, not significant

On the basis of gene expression patterns and cell trajectories, we concluded that transcriptional variation in HSC, MkP1, and MkP2 populations might be at the core of megakaryopoietic defects in ITP. DEG analysis indicated that the top upregulated genes in MkP1 of ITP included *HBB*, *HBG2*, and *AHSP*, which were associated with erythropoiesis, while the top downregulated genes in MkP2 of ITP included *CD9* and *TUBB1*, which are well-established genes related to thrombopoiesis, suggesting impaired megakaryopoiesis in ITP (Fig. 5a). We, therefore, focused on DEGs that affected the differentiation potential, and *HES1* and *CD9* were selected for further verification (Fig. 5b and Supplemental Fig. 5a–d). The role of the transcriptional repressor, *HES1*, in hematopoiesis has been well studied and it is known to participate in NOTCH signaling.^35^ Previous studies have shown that HES1 expression significantly increased during Mk differentiation, but remained statistically unchanged during erythrocyte differentiation.^36^ CD9, an integral membrane protein, is heterogeneously expressed in human HSPCs and has been used as a marker to identify MkP.^37^

To further confirm our findings, we collected BM samples from 27 ITP patients and 12 healthy donors (Supplemental Table 1). Multi-parameter flow cytometric analysis of the lineage (Lin)^−^CD34^+^CD45RA^−^ HSPCs compartment in BM samples that had been enriched using the magnetic human CD34 MicroBead kit was performed to compare the frequencies of HES1^+^ and CD9^+^ cells between ITP and HC (Fig. 5c). Results showed a reduction in the number of HES1^+^ cells and CD9^+^ cells in ITP, confirming the results of our single-cell transcriptome analysis (Fig. 5d, e).

### CD9^+^Lin^−^CD34^+^CD45RA^−^ HSPCs were biased toward megakaryopoiesis in vitro and were defective in ITP

It is well known that the dearth of appropriate markers for developmental stages is a major challenge in hematopoietic research. We hypothesized that CD9 may be a key marker of Mk differentiation. We collected BM samples from additional five ITP patients and four healthy donors (Supplemental Table 1), and then tested the differentiation potential of CD9^+^Lin^−^CD34^+^CD45RA^−^ HSPCs and CD9^−^Lin^−^CD34^+^ CD45RA^−^ HSPCs sorted from every sample using liquid culture assays (Fig. 5c).

We first assessed the dynamics of CD41 and CD235a expression. Results showed that CD9^+^ HSPCs tended to differentiate into megakaryocytes; however, this tendency was not observed in ITP patients and more erythrocytes were produced (Fig. 5f, g and Supplemental Fig. 6a). Morphological and ploidy-level analyses confirmed the differentiation into megakaryocytes in liquid culture systems (Fig. 5h and Supplemental Fig. 6b). We determined the expression of typical megakaryocyte markers (CD41a, CD61, and CD42a) after culturing for up to 4 weeks (Fig. 5i, j and Supplemental Fig. 6c). The percentage of megakaryocytes differentiated from CD9^+^Lin^−^CD34^+^CD45RA^−^ HSPCs was approximately threefold higher than that of the CD9^−^ counterpart in HC; in ITP patients, the percentage decreased to only 1/4th of that in HC, comparable to that from the CD9^−^Lin^−^CD34^+^ CD45RA^−^ HSPCs. This implied that the differentiation potential of CD9^+^ HSPCs, rather than that of CD9^−^ HSPCs, toward megakaryopoiesis was significantly impaired in ITP.

Collectively, the proportion of CD9^+^ cells in the Lin^−^CD34^+^ CD45RA^−^ HSPCs decreased in ITP. CD9 can be used to enrich Mk-biased HSPCs, and CD9^+^Lin^−^CD34^+^CD45RA^−^ HSPCs were implicated in the pathogenesis of ITP.

### Co-culture with pre-B cells from ITP markedly decreased the generation of megakaryocytes from CD9^+^Lin^−^CD34^+^CD45RA^−^ HSPCs

**Fig. 6:**
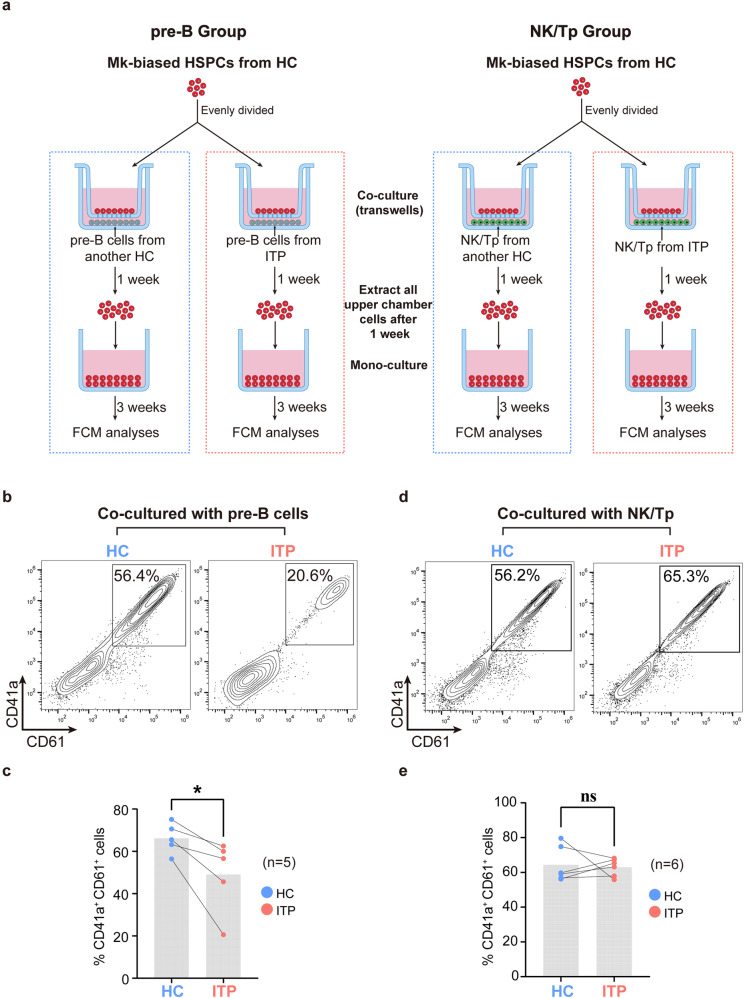
Co-culture of immune cell progenitors and Mk-biased HSPCs. **a** Experimental strategy. Schematic illustration of the transwell co-culture systems. Related to Fig. 6. **b**, **c** The proportion of CD41a^+^CD61^+^ cells derived from CD9^+^Lin^−^CD34^+^CD45RA^−^ HSPCs co-cultured with pre-B cells of HC and ITP, respectively. Two-tailed Student’s *t* test; *p < 0.05; *n* = 5. **d**, **e** The proportion of CD41a^+^CD61^+^ cells derived from CD9^+^Lin^−^CD34^+^CD45RA^−^ HSPCs co-cultured with NK/Tp cells of HC and ITP, respectively. Two-tailed Student’s *t* test; ns, not significant; *n* = 6

To confirm the interaction between immune cell progenitors and the Mk-biased HSPCs, we collected BM samples from additional 11 ITP patients and 12 healthy donors to establish transwell co-culture systems (Fig. 6a, Supplemental Fig. 6d, and Supplemental Table 1). Based on the results of cell-cell interaction analysis, pre-B cells and NK/Tp were selected and co-cultured with the Mk-biased HSPCs (Supplemental Fig. 6d). CD9^+^Lin^−^CD34^+^CD45RA^−^ HSPCs from one healthy donor were evenly divided into two upper chambers, while pre-B cells or NK/Tp from another healthy donor and an ITP patient were seeded into the corresponding two lower compartments. After one week of co-culture, all cells in each upper chamber were extracted and cultured separately for another three weeks (Fig. 6a). After culture for four weeks in total, the proportion of megakaryocytes in the CD9^+^Lin^−^CD34^+^CD45RA^−^ HSPC progeny decreased significantly in the group co-cultured with pre-B cells from ITP (Fig. 6b, c). However, there was no statistical difference in the NK/Tp groups (Fig. 6d, e). The data demonstrated that the defective megakaryopoiesis in ITP might be related to aberrant pre-B cells.

### Cellular heterogeneity during megakaryopoiesis across normal human ontogeny

**Fig. 7:**
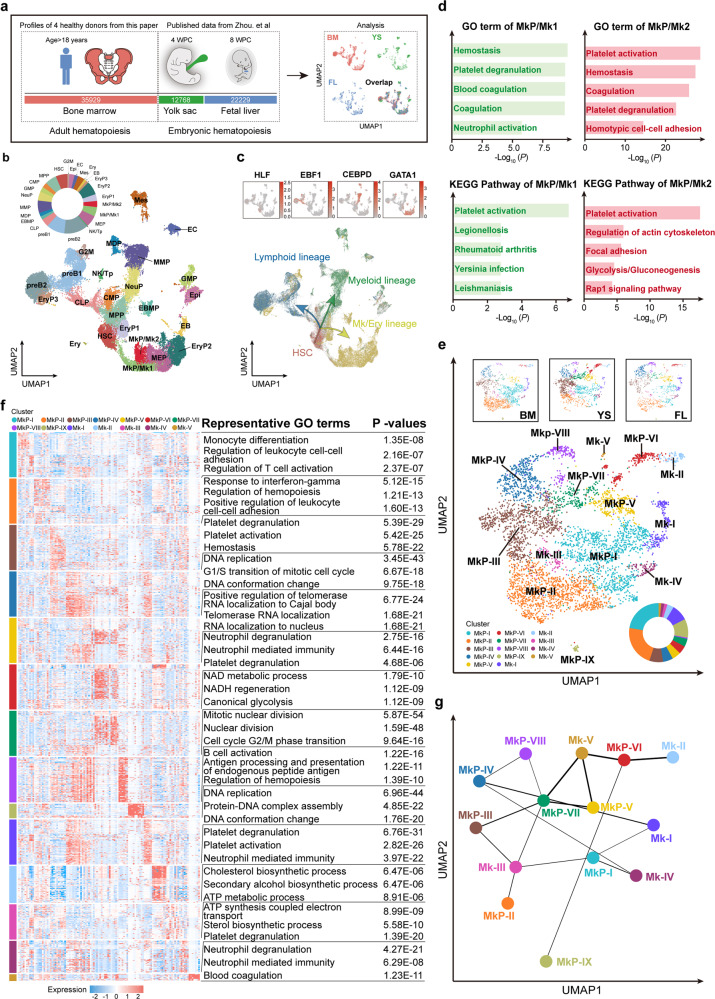
Transcriptomic analysis of different cell populations in human integrated dataset. **a** Schematic overview. Related to Fig. 7. **b** Cell clusters were visualized using UMAP. Colors indicate cell types. Each dot represents one cell. HSC hematopoietic stem cells, MPP multipotent progenitors, CMP common myeloid progenitors, GMP granulocyte and monocyte progenitors, NeuP neutrophil progenitors, MMP monocyte-macrophage progenitors, MDP monocyte-dendritic-cell progenitors, EBMP eosinophil-basophil-mast-cell progenitors, CLP common lymphoid progenitors, pre-B pre-B cells, NK/Tp natural killer/T cell progenitors, MEP megakaryocyte-erythroid progenitors, MkP/Mk megakaryocytic progenitors/megakaryocytes, EryP erythroid progenitors, EB erythroblasts, Ery erythrocytes, Mes mesenchymal cells, EC endothelial cells, Epi epithelial cells, G2M cells in G2/M phase. Pie chart showing the relative abundance of each cell cluster in the integrated dataset. **c** UMAP plots displaying the expression of four known marker genes (*HLF, EBF1, CEBPD*, and *GATA1*) during hematopoietic development. Arrows indicate the main directions of differentiation, inferred from the analysis of typical marker genes. **d** Top 5 GO: BP terms (upper) and KEGG pathways (lower) enriched in MkP/Mk1 (left) and MkP/Mk2 (right). **e** UMAP visualization of 14 subclusters resulted from sub-dividing the cells in MkP/Mk clusters, as described in Fig. 7b. Color according to subclusters. Pie chart showing the relative abundance of each subcluster. **f** Heat map of the top 10 significant DEGs and enriched GO terms in the MkP and Mk subclusters. See also Supplemental Fig. 12. **g** PAGA topology tree of MkP and Mk subclusters. Edge weights indicate the strength of the connectivity between clusters

To provide a foundation for cellular heterogeneity and transcriptional changes in ITP, we analyzed megakaryopoiesis across normal human multi-hematopoietic tissues. We integrated our single-cell transcriptome data of adult HC BM CD34^+^ HSPCs with a previously published single-cell dataset,^38^ which includes two yolk sac (YS) and two fetal liver (FL) scRNA-seq libraries, containing 12,768 and 22,229 single-cell profiles, respectively (Fig. 7a and Supplemental Fig. 7a–c).

These cells were further clustered into 24 clusters, including two megakaryocytic progenitor/megakaryocyte populations (MkP/Mk1 and MkP/Mk2) (Fig. 7b and Supplemental Fig. 7d). The heterogeneous cell populations were aligned with similar identity rather than with origin (Supplemental Fig. 7e). The expression of established markers in each cluster was clear (Supplemental Fig. 7f-h and Supplemental Table 5), which is consistent with the results shown in Fig. 2. On the basis of the expression of classical lineage-specific genes, the cells were divided into four continuous parts, indicating three main directions of differentiation (Fig. 7c and Supplemental Fig. 7i). Cells from BM, YS, and FL accounted for 39.4%, 31.4%, and 29.2% of the two MkP/Mk cells, respectively (Supplemental Fig. 7j).

GO term enrichment and Kyoto Encyclopedia of Genes and Genomes (KEGG) pathway analyses validated our identification of MkP/Mk populations. The major enriched biological processes and pathways were related to “hemostasis” and “platelet activation” which was consistent with their known functions (Fig. 7d), although the features might differ with origin (Supplemental Fig. 8a, b). To investigate cellular heterogeneity, the MkP/Mk clusters were further divided into 14 subclusters (Fig. 7e and Supplemental Fig. 8c, d). Assessment of the molecular features in each subpopulation revealed that MkP and Mk populations were heterogeneous at the transcriptional level (Fig. 7f, Supplemental Fig. 8e, f, and Supplemental Table 6). The immunomodulatory functions of MkP and Mk were non-negligible. Partition-based graph abstraction (PAGA) analysis, used to define cellular trajectories, revealed multiple putative developmental orientations (Fig. 7g).

The analysis of HSPCs across normal human ontogeny revealed the development process and differentiation trajectory in hematopoiesis and megakaryopoiesis, providing a foundation for constructing the differentiation model and annotating cell types in disease.

### Transcriptional changes in MkP subclusters of ITP

Our characterization of MkP/Mk from multi-hematopoietic tissues revealed the universality of their heterogeneity and provided an analytical basis for the study of megakaryocyte-related diseases, including ITP.

**Fig. 8:**
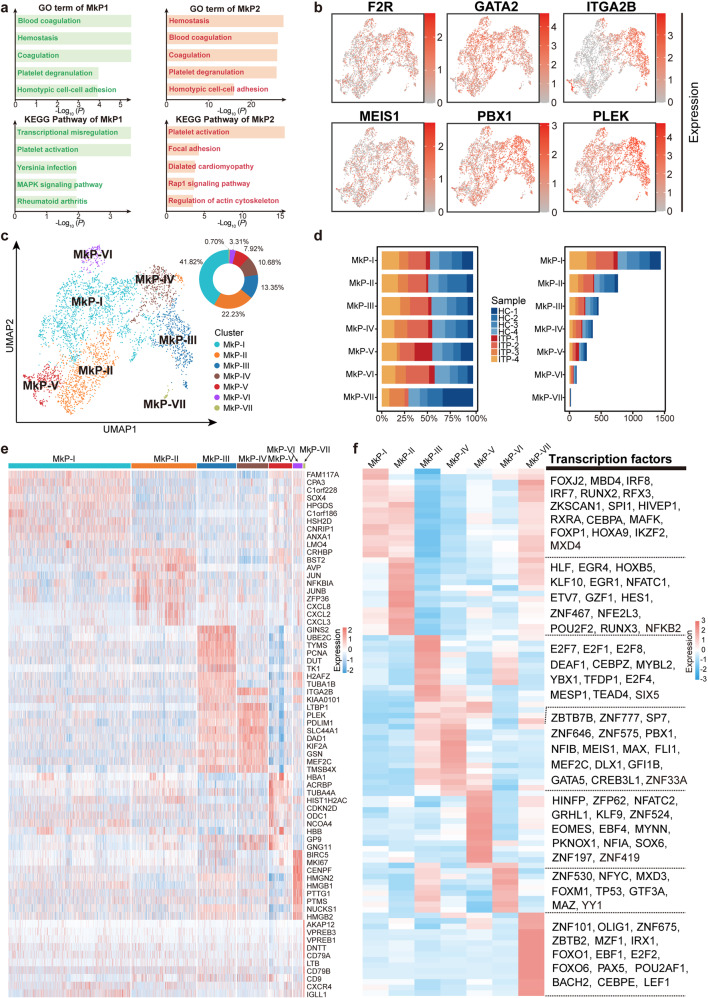
Detailed analysis of MkP populations in BM. **a** Top 5 GO: BP terms (upper) and KEGG pathways (lower) enriched in MkP1 (left) and MkP2 (right). **b** UMAP plots displaying the expression of six molecular features of MkP (*F2R*, *GATA2*, *ITGA2B*, *MEIS1*, *PBX1*, and *PLEK*). **c** UMAP visualization of seven subclusters, resulting from sub-dividing the BM cells in the MkP1 and MkP2 clusters described in Fig. 1c. Color according to subclusters. Pie chart showing the relative abundance of each subcluster. **d** Stacked barplots showing the numbers of cells from different sample sources in each subcluster (left), and proportions of cells from different sample sources in each subcluster (right). **e** Heat map showing the scaled expression of the top 10 marker genes in each MkP subcluster. See also Supplemental Fig. 13. **f** Top 15 differentially expressed TFs in each subcluster. See also Supplemental Fig. 14

**Fig. 9:**
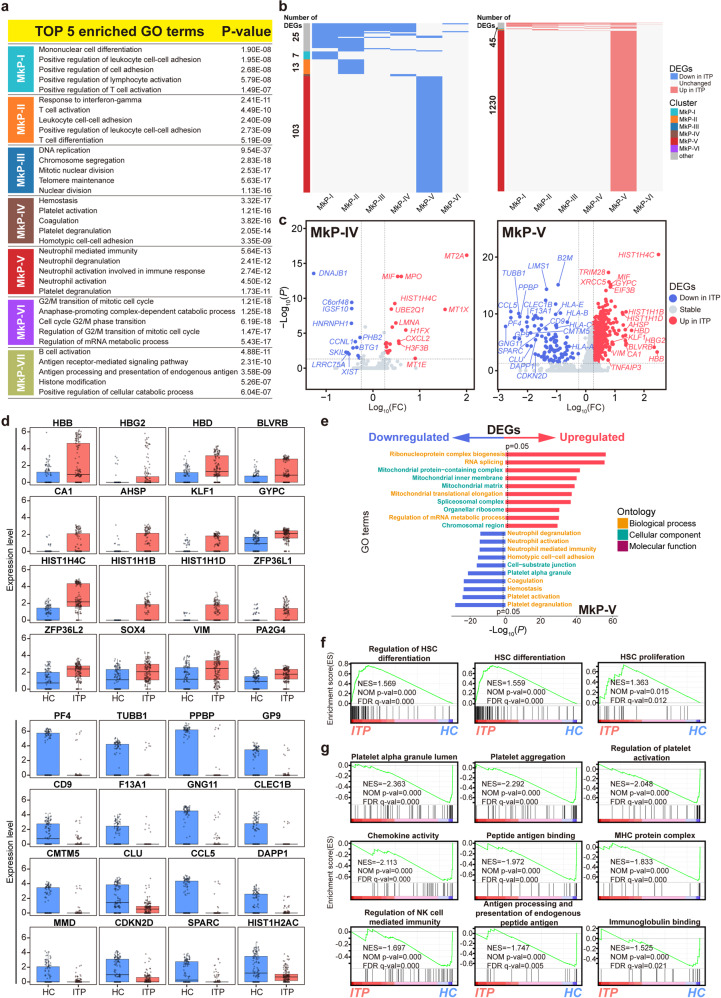
ITP-associated alterations in MkP subclusters. **a** TOP 5 GO: BP terms enriched in each MkP subcluster. **b** Heat maps showing the distribution of downregulated DEGs (left) and upregulated DEGs (right) between ITP and HC samples in each MkP subcluster. The gray bars on the left of the heat maps denote DEGs shared by at least two subclusters, while the others are subcluster-specific DEGs. **c** Volcano plots highlighting significant differences in gene expression between ITP and HC in MkP-IV (left) and MkP-V (right). Red points represent significantly upregulated genes, blue points represent significantly downregulated genes, and gray points represent non-differentially expressed genes. Genes with an adjusted *p* value < 0.05, log-transformed fold change absolute value >0.25, and minimum percentage >0.25 were considered as differentially expressed genes. **d** Boxplot showing the expression of DEGs in MkP-V. **e** Two-sided bar graph showing the top 10 enriched upregulated and downregulated GO terms in MkP-V in ITP. **f** GSEA plots showing representative pathways enriched in MkP-V from ITP versus HC. NES normalized enrichment score, NOM *p* value nominal *p* value, FDR *q* value false discovery rate *q* value. **g** GSEA plots showing representative pathways enriched in MkP-V from HC versus ITP. NES normalized enrichment score, NOM *p* value nominal *p* value, FDR *q* value false discovery rate *q* value

Enrichment analysis confirmed the definition of MkP in our ITP integrated dataset (Fig. 8a). To further analyze the transcriptional difference between ITP and HC, we pooled MkP from all samples. The expression of several molecular features of MkP was visualized and shown in Fig. 8b. Seven transcriptionally heterogeneous subpopulations of MkP were identified (Fig. 8c, d and Supplemental Fig. 9a–c). We then assessed the expression of marker genes in each subpopulation and characterized the gene sets enriched in different clusters (Fig. 8e, f, Fig. 9a, Supplemental Fig. 9d, and Supplemental Table 7), which showed pronounced heterogeneity within MkP, consistent with the results of previous analyses of MkP/Mk from multi-hematopoietic tissues.

We evaluated ITP-associated DEGs in each MkP subcluster (Fig. 9b and Supplemental Table 8). Notably, most of these DEGs were subtype-specific, indicative of the subtype-specific effects of the disorder. In particular, transcriptome alterations in MkP-V were the most prominent, as 98.5% of the upregulated genes and 82.4% of the downregulated genes in these subtype-specific genes occurred in MkP-V, suggesting that it was the subset with dual functions of immunomodulation and platelet generation that was most associated with ITP, rather than MkP-IV, the subset with relatively pure platelet-generating activity. We found that thrombopoiesis-related genes, such as *GP9, PF4, CD9*, and *PPBP*, were downregulated significantly in MkP-V, meanwhile, erythropoiesis-related genes, such as *HBB, HBD, CA1*, and *AHSP*, were upregulated significantly (Fig. 9c, d), consistent with the finding in Fig. 5a, suggesting a possible advanced location of ITP-affected MkP cells. GO analysis and GSEA revealed that platelet activation and cellular immunity pathways were significantly downregulated in MkP-V of ITP, while RNA splicing and regulation of HSC differentiation were significantly upregulated (Fig. 9e–g and Supplemental Fig. 9e).

Our results suggested that distinct subpopulations of MkP perform diverse functions, which contributed to the variation in their pathogenic significance in ITP. Irrespective of normal or pathological status, the immune function of MkP warrants further study.

## Discussion

Here, we provided a comprehensive single-cell transcriptomic atlas of HSPCs to characterize transcriptome alterations in newly diagnosed treatment-naive ITP. Our analysis revealed a distinct immune ecosystem in ITP BM, characterized by enhancement of immune-related genes and pathways during hematopoiesis, abundant transcriptome changes in pre-B and NK/Tp subpopulations, activation of immune crosstalk within HSPC clusters, and weakened immunomodulatory functions of an MkP subcluster, along with downregulation of platelet-generation potential. Functional assays demonstrated that the number of CD9^+^ cells in Lin^−^CD34^+^CD45RA^−^ HSPCs decreased in ITP, and the differentiation of CD9^+^Lin^−^CD34^+^CD45RA^−^ HSPCs toward the megakaryopoietic lineage was impaired. We constructed the single-cell transcriptome profile of BM CD34^+^ HSPCs in ITP for the first time, providing valuable resources for future research on the molecular mechanism of HSPCs underlying ITP.

In ITP, various immune cells participate in autoimmune progression.^2,39,40^ Here, using trajectory analysis, we demonstrated that immune states were activated during most phases of hematopoiesis in ITP, suggesting that HSPCs are components of autoimmunity in ITP. We observed upregulation of the complement activation pathway in HSPCs, which might be stimulated by platelet autoantibodies^41,42^ and may damage platelets and megakaryocytes.^43^ This suggested that complement inhibitors, such as sutimlimab,^44^ may be effective in ITP, not only by acting on circulating mature cells but also by regulating the complement system of BM HSPCs.

We identified various progenitors of immune cells and found a trend of reduced pre-B cells in ITP BM, which was consistent with a previous report of decreased total B cells in ITP BM.^45^ In humans, BM is the major site of B cell development where the pre-B cells differentiated into mature B cells.^46^ Generally, antibodies produced by autoreactive B cell clones lead to platelet destruction and thrombopoiesis defects in ITP.^39^ In our dataset, the pre-B cells had obvious immune dysregulation in ITP, which might promote the pro-inflammatory environment and defective megakaryopoiesis in ITP. Considering that preB3 was an important participant in autoimmunity of BM HSPCs in ITP, we investigated the crosstalk between preB3 and Mk/Ery-lineage cells, and discovered many specific interaction pairs mainly related to apoptotic signals, immune chemotaxis, and cell adhesion, that might mediate immune attacks. Interestingly, we found a series of collagen and integrin pairs to be expressed specifically in ITP.^47,48^ These interactions might lead to an intensive immune attack and decrease platelet production capacity in MkP.

According to our trajectory analysis, HSCs preferentially differentiated towards the Mk lineage in both ITP and HC, but the potential was not entirely consistent. We also identified CD9, a well-known marker of the Mk lineage,^37,49^ which was significantly downregulated in ITP. Here, we report for the first time that the CD9^+^ subpopulation of Lin^−^CD34^+^CD45RA^−^ HSPCs decreased remarkably in ITP, and developed a strategy to enrich Mk-biased HSPCs (CD9^+^Lin^−^CD34^+^CD45RA^−^). Our data indicate that alterations of pre-B cells, which we observed in ITP patients, contribute to the impaired megakaryopoiesis in ITP. Pre-B cells could proliferate autonomously in vitro and persist for a long time. The addition of IL-7 enhanced pre-B cell expansion and prevented maturation.^50^ Thus, based on the results of our co-culture experiments, there might be a direct interaction between pre-B cells and Mk-biased HSPCs.

It is widely accepted that platelets and megakaryocytes perform significant immunomodulatory functions.^51,52^ However, whether the immunomodulatory functions are inherited from progenitor cells or are acquired from response to inflammation remains unclear. In addition, whether the immunomodulatory functions are possessed by distinct subpopulations with different identities or are shared by all cells is unknown. Our results for MkP/Mk from multi-hematopoietic tissues indicated that both human MkP and Mk are composed of multiple subpopulations with different gene expression and functional patterns, which was consistent with the results of previous studies.^38,53,54^ We found that immunomodulatory functions tended to be performed by specific subsets, and that the immunomodulatory functions of Mk might be inherited from MkP subpopulations with similar functions. Interestingly, the separation between platelet generation and immunomodulatory functions was not as clear as it was among other functions. There were several subclusters with dual functions of immune modulation and platelet generation, suggesting that these two functions might be closely related and may interact with each other. Analysis of MkP in ITP revealed that most of the ITP-associated DEGs were subtype-specific and were concentrated in a subcluster, possessing dual functions of immunomodulation and platelet generation. These two functions of this subcluster were downregulated in ITP, indicating that this MkP might be the main target of autoimmune attack in ITP, and that they might also be able to regulate autoimmunity against themselves, through a mechanism similar to negative feedback. Further research is required to test this interesting conjecture.

However, the study has certain limitations. A large part of our work is based on bioinformatics analysis, predictions of which have to be carefully validated experimentally. In addition, considering the complexity of ITP, our study was limited to newly diagnosed treatment-naïve adult ITP. In the future, our study will focus on patients with a good response to glucocorticoid therapy and patients with relapsed/refractory ITP.

In summary, this is the first study to show the significant heterogeneity and disease characteristics of BM CD34^+^ HSPCs from ITP patients at single-cell resolution using transcriptomic profiling, revealing new insights regarding the pathophysiology of ITP.

## Materials and methods

### Clinical sample collection

BM samples were obtained from 47 ITP patients (26 females and 21 males; age range: 19–70 years; median age: 37 years; platelet count range: 1–60 × 10^9^ /L; median platelet count: 21 × 10^9^/L) based on previously published criteria for ITP^1^ and 32 healthy donors (18 females and 14 males; age range, 19–65 years; median age, 42.5 years; platelet count range, 158–301 × 10^9^/L; median platelet count, 216.5 × 10^9^/L) (Table 2 and Supplemental Table 1). This study was approved by the Medical Ethical Committee of the Qilu Hospital, Shandong University. Informed consent was obtained from all patients and controls in accordance with the Declaration of Helsinki.

**Table 2:** Baseline characteristics of patients with ITP and healthy donors

	ITP patients	Healthy controls	
	(*n* = 47)	(*n* = 32)	*P*
Sex			>0.05
Female	26 (55.32)	18 (56.25)	
Male	21 (44.68)	14 (43.75)	
Age	37 (19–70)	42.5 (19–65)	>0.05
Platelet count			
(10^9^ per L)	21 (1–60)	216.5 (158–301)	<0.001

Data are median (range) or *n* (%)

All patients were newly diagnosed with ITP with no previous record of ITP treatment, characterized by isolated thrombocytopenia (peripheral blood platelet count <100 × 10^9^/L). The thrombocytopenia due to other causes was ruled out through a detailed inquiry of family history, physical examination, complete blood count, and BM morphology.^1^

### Single-cell RNA library preparation

BM samples were obtained by iliac crest aspiration, and removed erythrocytes by incubating with RBC lysis buffer (R1010, Solarbio, Beijing, China) for 8 minutes. BM cells were stained with APC anti-human CD34 antibody (343608, Biolegend, San Diego, CA, USA) in DPBS (14190144, Gibco, Grand Island, NY, USA) with 0.1% BSA (130-091-376, Miltenyi Biotec GmbH, Bergisch Gladbach, Germany) on ice for 30 minutes in the dark followed by two washes, and resuspended in DPBS with 0.1% BSA containing DAPI viability dye (1 μg/ml, D9542, Sigma-Aldrich, St. Louis, MO, USA), and sorted on a BD FACSAria III (BD Biosciences, San Jose, CA, USA), and the purity of sorted populations as verified by flow cytometry analysis was >90%.

The scRNA-seq dataset of each sample were produced with a Chromium system (10X Genomics, PN1000073, Pleasanton, CA, USA) following the manufacturer’s instructions. Cell viability was determined by trypan blue stain (T10282, Invitrogen, Carlsbad, CA, USA) with TC20 automated cell counter (Bio-rad Laboratories, Hercules, CA, USA). The ratio of viable cells in single-cell suspension was >90%. The concentration of single-cell suspension was adjusted to 700–1200 cells/μL. Suspended cells were loaded on a Chromium Controller (10X Genomics, Pleasanton, CA, USA) to generate Gel beads in EMulsion (GEMs). Cells were lysed, and the cytoplasmic RNA was fireleased. All barcoding steps with reverse transcription were finished in individual GEMs. Libraries were sequenced by the Illumina NovaSeq 6000 (Illumina, San Diego, CA, USA) with 150-bp paired-end reads.

### Single-cell RNA sequencing data processing

Raw files including the previously published datasets of yolk sac and fetal liver cells were processed with Cell Ranger 3.0.0 pipeline (10X Genomics, Pleasanton, CA, USA) using default mapping arguments. Reads were mapped to the human genome (GRCh38-1.2.0).

### Quality filtration of cells and Batch effects correction

We used Seurat R package (version 3.1)^55,56^ to analyze scRNA-seq data. The following criteria were applied to each cell of all samples including yolk sac and fetal liver samples of the previously published datasets: unique gene number >500, unique molecular identifier (UMI) count >1000 and mitochondrial gene percentage <0.15. Genes expressed in at least three cells were included. Besides, we filtered out cells with high ribosomal and heat shock protein gene percentage by setting a threshold that removes the last 2.5% of the data. In addition, we used DoubletFinder R package (version 2.0.3)^57^ to remove doublets. Finally, all ribosomal, mitochondrial and heat shock protein genes were removed.

We used the MergeSeurat function to merge datasets. The NormalizeData function was used to normalize the raw counts, and the FindVariableFeatures function was used to identify highly variable genes. Datasets were scaled and centered using the ScaleData function, and UMI numbers, the mitochondrial gene percentages, the ribosomal gene percentages, and the heat shock protein gene percentages were regressed out. Afterward, Harmony R package (version 1.0)^58^ was used to avoid the batch effect affecting downstream analysis.

### Dimensionality reduction and clustering

The first 20 harmony-corrected PCA embeddings and resolution 0.5 were used for dimensionality reduction and clustering on the integrated dataset consisted of ITP and HC BM transcriptional profiles, and the first 40 harmony-corrected PCA embeddings and resolution 1.5 were used for dimensionality reduction and clustering on the integrated dataset consisted of healthy human BM, YS, and FL transcriptional profiles. Cells were projected into a two-dimension space using UMAP or t-SNE. Cell cluster annotation was based on HSPC subsets specific marker genes reported previously.

### Differential expression analysis and enrichment analysis

The FindAllMarkers function implemented in Seurat was used to identify DEGs in each cluster. GO term enrichment and KEGG pathway analysis were performed by ClusterProfiler R package (version 4.0.5).^59^ GSVA scores were calculated by GSVA R package (version 1.34.0).^60^ GSEA software (version 4.1.0)^61^ was used to calculate the distribution of gene sets in lists of genes ordered by population expression differences. Normalized enrichment score (NES), nominal *P* value, and false discovery rate (FDR) *q* value were used for the statistical analysis. The pathways with |NES|> 1, NOM *p* value < 0.05, and FDR *q* value <0.25 were considered to be meaningful.

### Trajectory analysis

PAGA^62^ and Monocle2 R package (version 2.20.0)^26,27^ were used for the trajectory analysis. For PAGA, we imported the integrated matrix from Seurat into Scanpy (version 1.8.1)^63^ by constructing an Anndata data structure. Then we computed the neighborhood graph through the sc.pp.neighbors function and constructed the PAGA graph through the sc.tl.paga function. The sc.tl.draw_graph function was used for visualization. A threshold of 0.25 was set to discard low-connectivity edges. For Monocle2, we selected the top 1100 significant DEGs among all cell types and the top 500 significant DEGs among Mk/Ery lineages identified by the differentialGeneTest function as the ordering genes, respectively. Dimensionality reduction and trajectory construction were performed on the selected genes with default methods and parameters. For identification of major patterns along the pseudotime, the top 2000 pseudotime-dependent genes were selected by the differentialGeneTest function with setting the parameters “fullModelFormulaStr” as “~sm.ns(Pseudotime)” and were clustered into four distinct patterns by k-means clustering. BEAM analysis was used to get the expression patterns in branches during development.^64^

### Transcriptional regulatory network analysis

A single-cell regulatory network on cells passing the quality controls for each group was constructed using pySCENIC (version 0.11.1).^65^ GRNBoost2 in pySCENIC was used to build regulatory networks from raw count data. The ctx module was then used to predict potential direct-binding targets (regulons), which were pruned with cis-regulatory motif discovery (cisTarget) databases. Finally, activity of regulons in individual cells was scored by the aucell function. To measure the difference between ITP patients and healthy donors, the regulon activity scores in each sample were scaled and calculated using the Limma R package (version 3.42.2)^66^ with adj. *p* value < 0.05 and |logFoldChange|>0.25. The regulon activity heat maps were generated with the pheatmap R package (version 1.0.12). Significant TF motifs shared by at least two ITP samples would be selected for visualization. The regulon activity heat maps were generated with the pheatmap R package (version 1.0.12).

### Cell-cell interaction analysis

CellPhoneDB python package (version 2.1.7)^67^ was used to detect ligand-receptor interactions and predict communications among different subclusters of HSPCs. The lower cutoff for expression proportion of any ligand or receptor in each cell cluster was set to 5%, and the *p* value threshold was set to 0.05.

### Antibodies and flow cytometry

BM CD34^+^ cells were enriched using a magnetic human CD34 MicroBead Kit (130-046-703, Miltenyi Biotec GmbH, Bergisch Gladbach, Germany), and stained with Brilliant Violet 510 anti-human lineage cocktail (CD3, CD14, CD16, CD19, CD20, CD56; 348807, Biolegend, San Diego, CA, USA), APC anti-human CD34 antibody (343608, Biolegend, San Diego, CA, USA), APC/Cyanine7 anti-human CD45RA antibody (304128, Biolegend, San Diego, CA, USA), PE anti-human CD9 antibody (312105, Biolegend, San Diego, CA, USA), Foxp3/Transcription Factor staining buffer set (00-5523-00, eBioscience, San Diego, CA, USA), anti-Hes1 antibody (1:100; ab108937, Abcam, Cambridge, UK), and Alexa Fluor 488 secondary antibody (1:200; A21206, Invitrogen, Carlsbad, CA, USA) for verifying downregulation of HES1 and CD9 in clinical ITP patients. Human CD34 MicroBead Kit (130-046-703, Miltenyi Biotec GmbH, Bergisch Gladbach, Germany), Brilliant Violet 510 anti-human lineage cocktail (CD3, CD14, CD16, CD19, CD20, CD56; 348807, Biolegend, San Diego, CA, USA), APC anti-human CD34 antibody (343608, Biolegend, San Diego, CA, USA), APC/Cyanine7 anti-human CD45RA antibody (304128, Biolegend, San Diego, CA, USA), PE anti-human CD9 antibody (312105, Biolegend, San Diego, CA, USA), FITC anti-human CD38 antibody (303503, Biolegend, San Diego, CA, USA), Brilliant Violet 421 mouse anti-human CD10 antibody (562902, BD Biosciences, San Jose, CA, USA), Brilliant Violet 421 anti-human CD161 antibody (339914, Biolegend, San Diego, CA, USA), and DAPI viability dye (1 μg/ml, D9542, Sigma-Aldrich, St. Louis, MO, USA) were used for cell sorting on a BD FACSMelody (BD Biosciences, San Jose, CA, USA) for in vitro culture. PE anti-human CD41 antibody (303706, Biolegend, San Diego, CA, USA), APC/Cyanine7 anti-human CD235a antibody (349116, Biolegend, San Diego, CA, USA), FITC anti-human CD41a antibody (11-0419-42, eBioscience, San Diego, CA, USA), APC anti-human CD41 antibody (303710, Biolegend, San Diego, CA, USA), APC anti-human CD42b antibody (303912, Biolegend, San Diego, CA, USA), eFluor450 anti-human CD42a antibody (48-0428-42, eBioscience, San Diego, CA, USA), PE anti-human CD61 antibody (130-081-501, Miltenyi Biotec GmbH, Bergisch Gladbach, Germany), PE anti-human CD61 antibody (555754, BD Biosciences, San Jose, CA, USA), Zombie NIR Fixable Viability Kit (423105, Biolegend, San Diego, CA, USA), Hoechst 33342 (H1399, Invitrogen, Carlsbad, CA, USA), and DAPI viability dye (1 μg/ml, D9542, Sigma-Aldrich, St. Louis, MO, USA) were used for cultured cells assays. Flow cytometric analysis was conducted on a Gallios flow cytometer (Beckman Coulter, Brea, CA, USA). Data were analyzed using Kaluza Analysis (version 2.1, Beckman Coulter, Brea, CA, USA) and FlowJo software (version 10.8.1, Tree Star, Ashland, OR, USA).

### In vitro culture

CD9^+^Lin^−^CD34^+^CD45RA^−^ and CD9^−^Lin^−^CD34^+^CD45RA^−^ HSPCs were cultured in 96-well plates with StemSpan SFEM II medium (09655, STEMCELL Technologies, Vancouver, BC, Canada) supplemented with recombinant human stem-cell factor (rhSCF; 50 ng/mL; PeproTech, Rocky Hill, NJ, USA), rhIL-3 (50 ng/mL; PeproTech, Rocky Hill, NJ, USA), rhIL-6 (50 ng/mL; PeproTech, Rocky Hill, NJ, USA), rhIL-9 (50 ng/mL; PeproTech, Rocky Hill, NJ, USA), rh–G colony-stimulating factor (G-CSF; 50 ng/mL; PeproTech, Rocky Hill, NJ, USA), rh–GM colony-stimulating factor (GM-CSF; 50 ng/mL; PeproTech, Rocky Hill, NJ, USA), rh-thrombopoietin (TPO; 50 ng/mL; PeproTech, Rocky Hill, NJ, USA), and 20% BIT 9500 Serum Substitute (09500, STEMCELL Technologies, Vancouver, BC, Canada). Besides, 24-well Transwells (3470, Corning Incorporated, Corning, NY, USA) were used for the indirect co-culture of immune cell progenitors and CD9^+^Lin^−^CD34^+^CD45RA^−^ HSPCs, and additional rhIL-7 (50 ng/mL; PeproTech, Rocky Hill, NJ, USA) was added in the transwell co-culture system. Lin^−^CD34^+^CD45RA^+^CD38^+^CD9^+^CD10^+^ and Lin^−^CD34^+^CD45RA^+^CD38^+^CD161^+^ were used for pre-B cells and NK/Tp enrichment, respectively (Supplemental Fig. 6d).^68^ All cultures were incubated at 37 °C in a humidified chamber under 5% carbon dioxide.

### Morphological observation

For Wright-Giemsa staining, cultured cells were centrifuged (at 400 × *g* for 2 minute) onto glass slides, and cytospin specimens were subjected to Wright–Giemsa staining, and examined by light microscopy (microscope, Leica DM2000, N PLAN ×100/1.25 Oil, Leica Microsystems GmbH, Wetzlar, Germany; Digital camera, LONGBASE 1010, LONGBASE, Qingdao, China). For immunofluorescence staining, cultured cells were stained with anti-CD41 antibody (1:200; ab134131, Abcam, Cambridge, UK), Alexa Fluor 488 secondary antibody (1:2000; A21206, Invitrogen, Carlsbad, CA, USA), and Flouroshield containing DAPI (ab104139, Abcam, Cambridge, UK), and were dropped onto glass slides, and examined by immunofluorescence microscopy (EVOS FL Auto 2, Invitrogen, Carlsbad, CA, USA).

### Statistical analysis

Statistical analyses were performed using R (version 3.6.1). The chi-square test was used for the comparison of classification measurements in Table 2. The two-tailed Student’s *t* test was used to compare numerical variables in Table 2, the proportion of CD9 or HES1 in Lin^−^ CD34^+^CD45RA^−^ HSPCs between ITP and HC, and the proportion of megakaryocytes in co-culture assays. The assessment of CD41 and CD235a expression in liquid culture system underwent variable transformation (arcsine square root transformed) and one-way ANOVA. The assessment of CD41a and CD61 expression in liquid culture system underwent variable transformation (arcsine square root transformed) and one-way ANOVA with Scheffe’s post hoc test.

## Supplementary information

Supplemental Material

Supplemental Fig. 1

Supplemental Fig. 2

Supplemental Fig. 3

Supplemental Fig. 4–1

Supplemental Fig. 4–2

Supplemental Fig. 4–3

Supplemental Fig. 4–4

Supplemental Fig. 4–5

Supplemental Fig. 5

Supplemental Fig. 6

Supplemental Fig. 7–1

Supplemental Fig. 7–2

Supplemental Fig. 8–1

Supplemental Fig. 8–2

Supplemental Fig. 9

Supplemental Fig. 10

Supplemental Fig. 11

Supplemental Fig. 12

Supplemental Fig. 13

Supplemental Fig. 14

Supplemental Table 1. Detailed information of the human BM samples used in this study

Supplemental Table 2. Cluster-specifically expressed genes in BM CD34+ HSPCs

Supplemental Table 3. DEGs between ITP and HC in HSPC subsets

Supplemental Table 4. Significant interacting pairs between preB3 or preB1 or NK/Tp and Mk/Ery-lineage cells (HSCs, MkP1, MkP2, MEP, and EryP)

Supplemental Table 5. Cluster-specifically expressed genes in cells came from 4 BM samples, 2 YS samples, and 2 FL samples

Supplemental Table 6. Cluster-specifically expressed genes in MkPMk subclusters from 4 BM samples, 2 YS samples, and 2 FL samples

Supplemental Table 7. Cluster-specifically expressed genes in MkP subclusters from ITP and HC BM samples

Supplemental Table 8. DEGs between ITP and HC samples in each MkP subcluster

## Data Availability

Codes for bioinformatics analysis are available at https://github.com/liuyanxys/Code-for-itp.git. The scRNA-seq data have been deposited in the Gene Expression Omnibus (GEO) database with accession number GSE196676.
