# Supplementary material for: Deciphering transcriptome alterations in bone marrow hematopoiesis at single-cell resolution in immune thrombocytopenia
Source: Signal Transduct Target Ther. 2022 Oct 7;7:347. doi: 10.1038/s41392-022-01167-9 (PMC9537316; doi:10.1038/s41392-022-01167-9)
Supplement: Supplementary file 1 — Supplemental Material [file 41392_2022_1167_MOESM1_ESM.docx]

Supplementary Materials for

Deciphering transcriptome alterations in bone marrow hematopoiesis at single-cell resolution in immune thrombocytopenia

Yan Liu^1,2,*^, Xinyi Zuo^1,3,*^, Peng Chen^1,2,*^, Xiang Hu^1^, Zi Sheng^1^, Anli Liu^1^, Qiang Liu^1^, Shaoqiu Leng^1^, Xiaoyu Zhang^1^, Xin Li^1^, Limei Wang^4^, Qi Feng^1,5^, Chaoyang Li^5^, Ming Hou^1,5,6^, Chong Chu^7^, Shihui Ma^2^, Shuwen Wang^1,5^, and Jun Peng^1,2,4^

Correspondence to: junpeng88@sina.com.cn

**This file includes:**

Supplemental Fig. 1 to 14

**Other Supplementary Materials for this manuscript include the following:**

Supplemental Table 1 to 8

Supplemental Table 1. Detailed information of the human BM samples used in this study.

Supplemental Table 2. Cluster-specifically expressed genes in BM CD34^+^ HSPCs.

Supplemental Table 3. DEGs between ITP and HC in HSPC subsets.

Supplemental Table 4. Significant interacting pairs between preB3 or preB1 or NK/Tp and Mk/Ery-lineage cells (HSCs, MkP1, MkP2, MEP, and EryP).

Supplemental Table 5. Cluster-specifically expressed genes in cells came from 4 BM samples, 2 YS samples, and 2 FL samples.

Supplemental Table 6. Cluster-specifically expressed genes in MkP/Mk sub-clusters from 4 BM samples, 2 YS samples, and 2 FL samples.

Supplemental Table 7. Cluster-specifically expressed genes in MkP sub-clusters from ITP and HC BM samples.

Supplemental Table 8. DEGs between ITP and HC samples in each MkP sub-cluster.

Figure. S1.


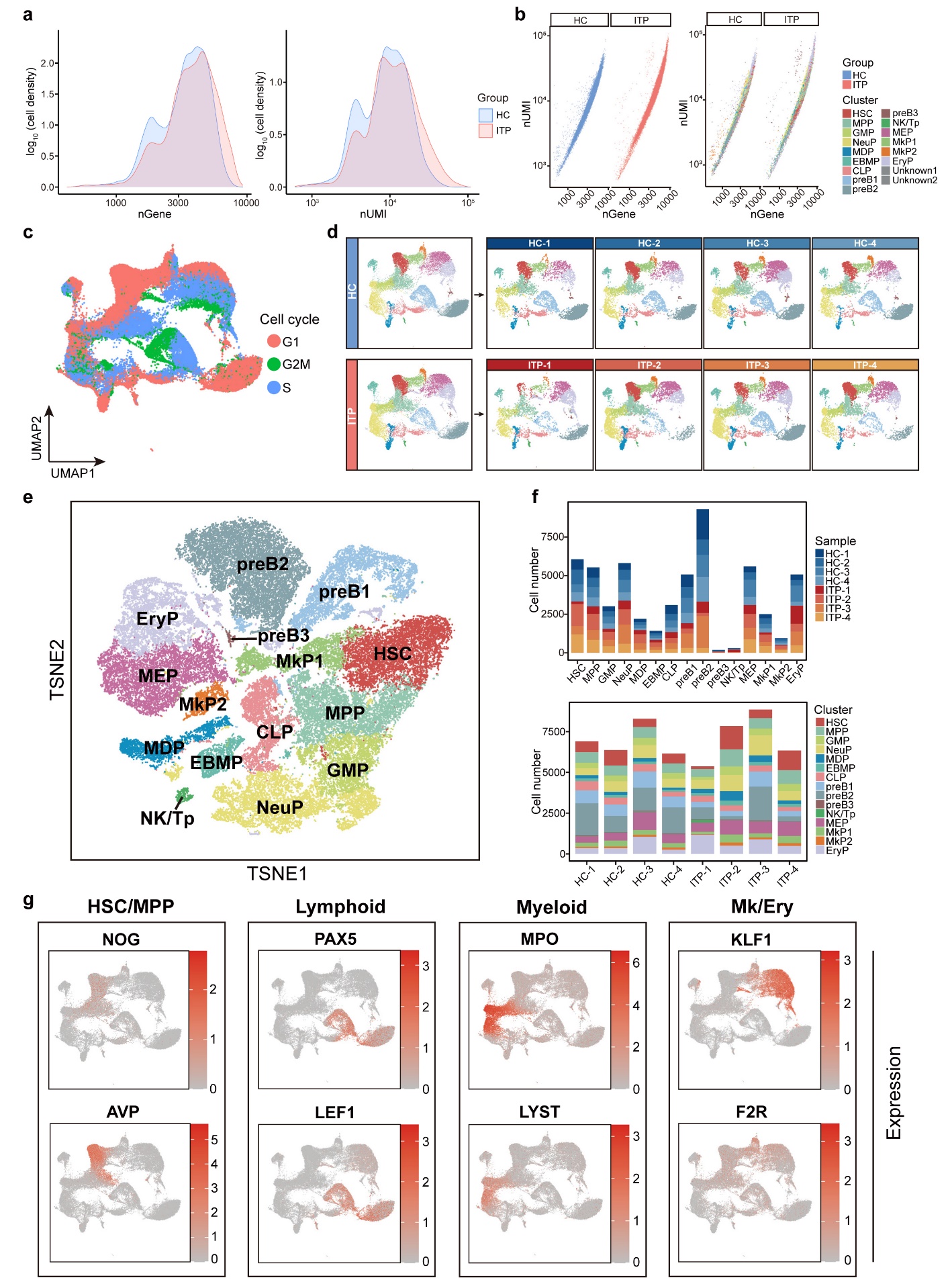


**Supplemental Fig. 1** **Detailed characterization of HSPCs from ITP and HC bone marrows, related to Fig. 1.** (a) Distribution of number of genes and UMIs detected per cell after filtering in ITP (red) and HC (blue) groups. (b) Scatter plot showing the linear correlation between the number of genes and the number of UMIs detected in HSPCs. Colors indicate groups (left) and cell types (right). (c) UMAP plot of scRNA-seq profiles. Each dot represents a cell and is colored based on the cell-cycle phase predicted by Seurat. (d) UMAP plot showing cell clusters. Colors indicate cell types. ITP and HC samples are shown separately. (e) Cell clusters visualized using t-SNE. Colors indicate cell types. Each dot represents one cell. (f) Histogram showing the cell number of sample contributions per annotated cell type (upper) and the cell number of annotated cell type contributions per sample (lower). (g) UMAP plots displaying the expression of canonical marker genes during hematopoietic development.

Figure. S2.


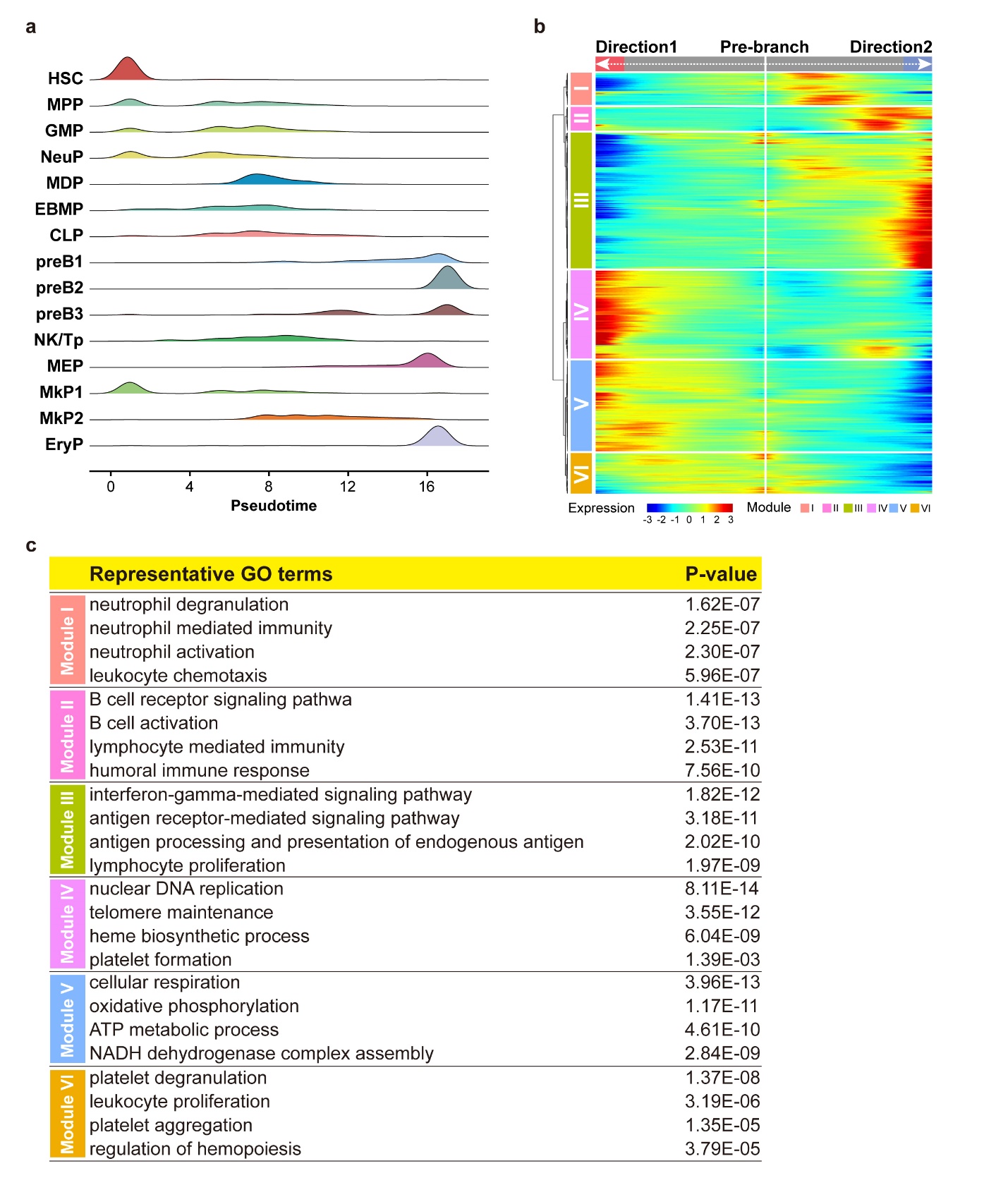


**Supplemental Fig. 2 Analysis of HSPC transition states in ITP and HC samples, related to Fig. 2.** (a) The distribution of HSPC types along the pseudotime. Colors represent the cell clusters shown in Fig. 1c. (b) Heat map depicting the expression of the branch-dependent genes over pseudotime. Genes are clustered to six modules based on expression patterns across pseudotime. The branch point shown in the middle of heat map is the beginning of pseudotime. Both sides of heat map are the ends of pseudotime. Color bar indicates the relative expression level. Directon1 matches the left branch and Directon2 matches the right branch in Fig. 2b. (c) Top 4 enriched GO: BP terms and p value of each module.

Figure. S3.


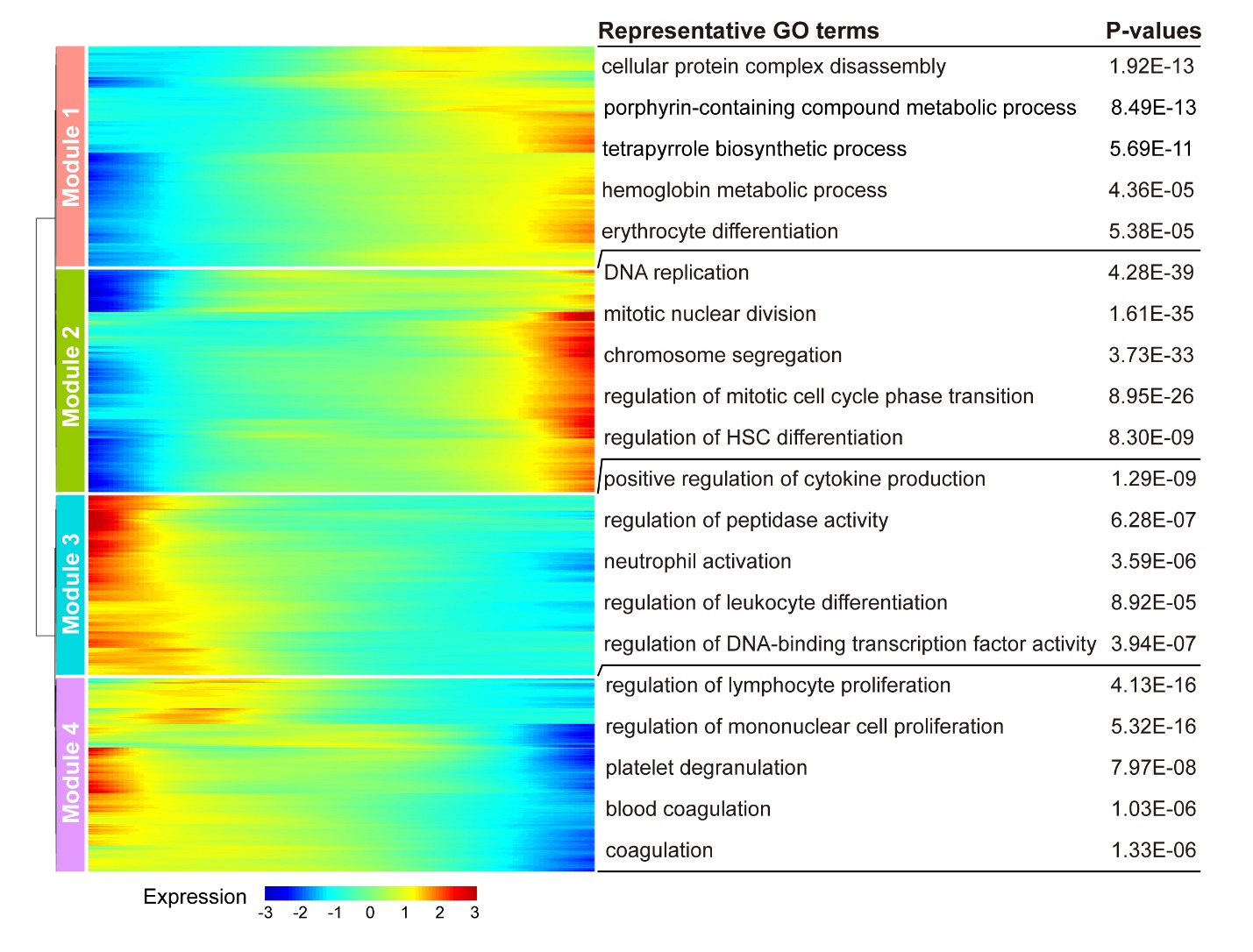


**Supplemental Fig. 3 Transition state analysis of Mk/Ery lineages in ITP and HC samples, related to Fig. 3.** Heat map showing dynamic changes in gene expression along the pseudotime (left). Representative GO: BP terms of each module (right). Adjusted p value less than 0.05 was considered statistically significant for GO enrichment analysis.

Figure. S4.


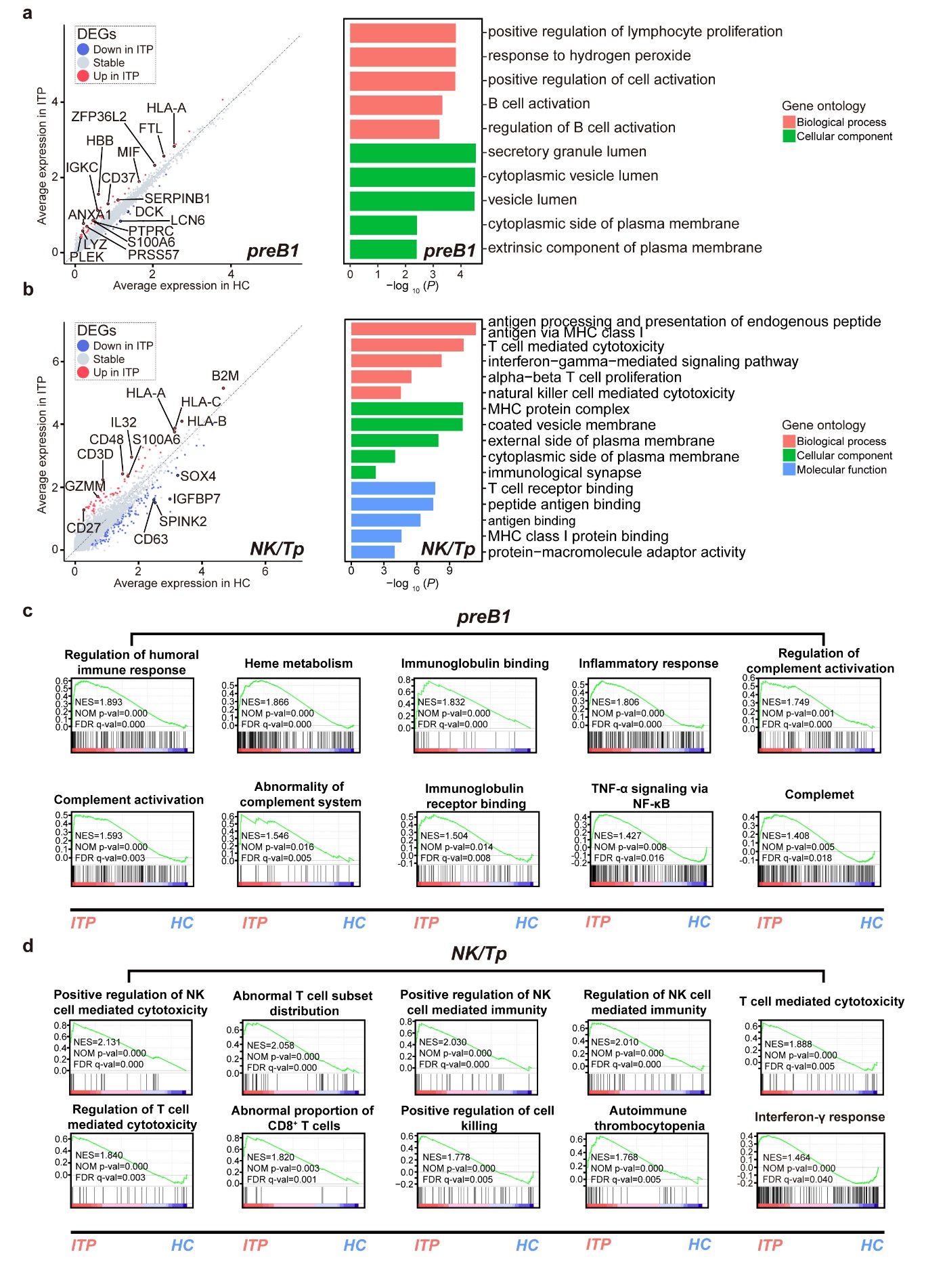


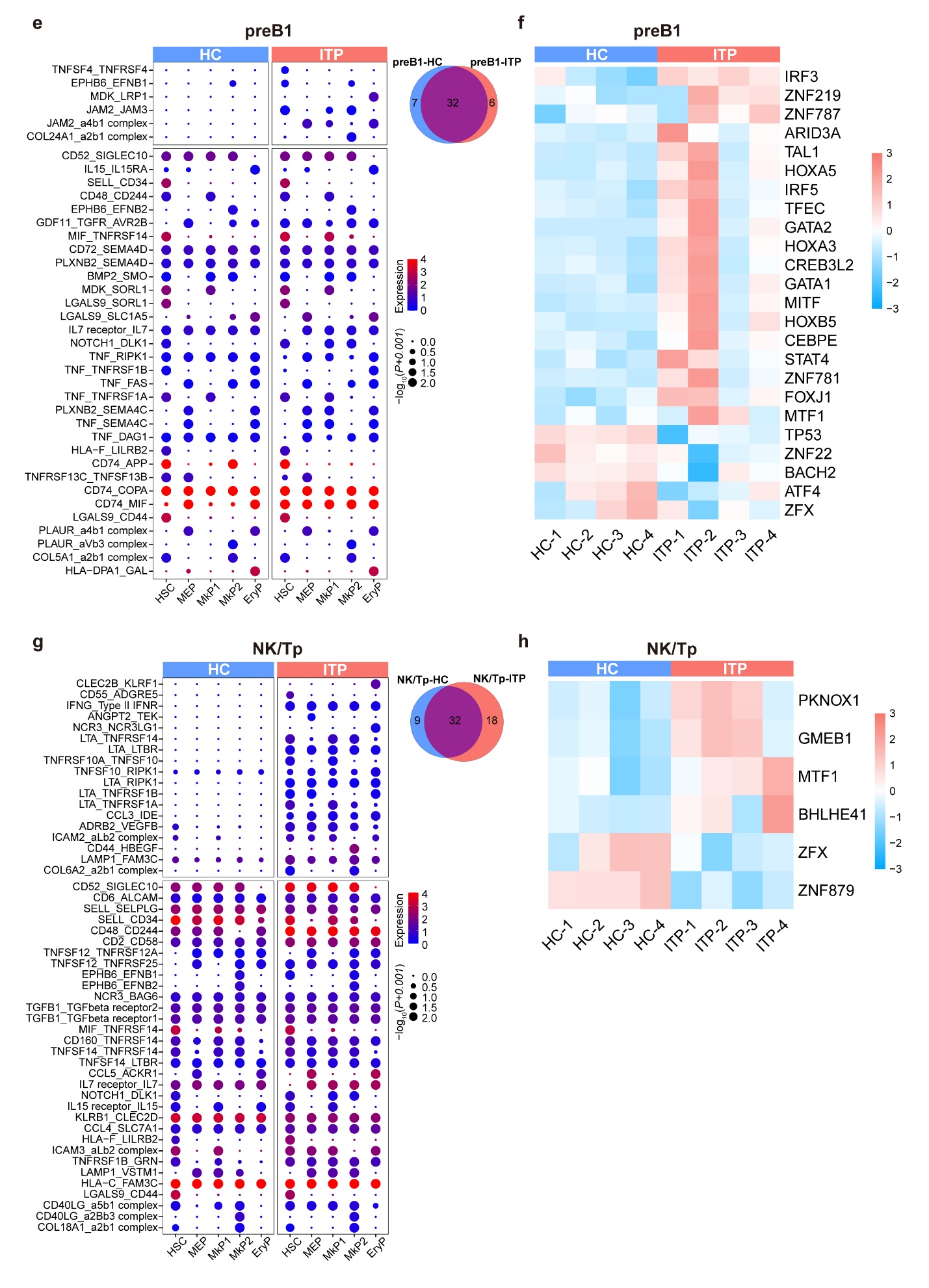


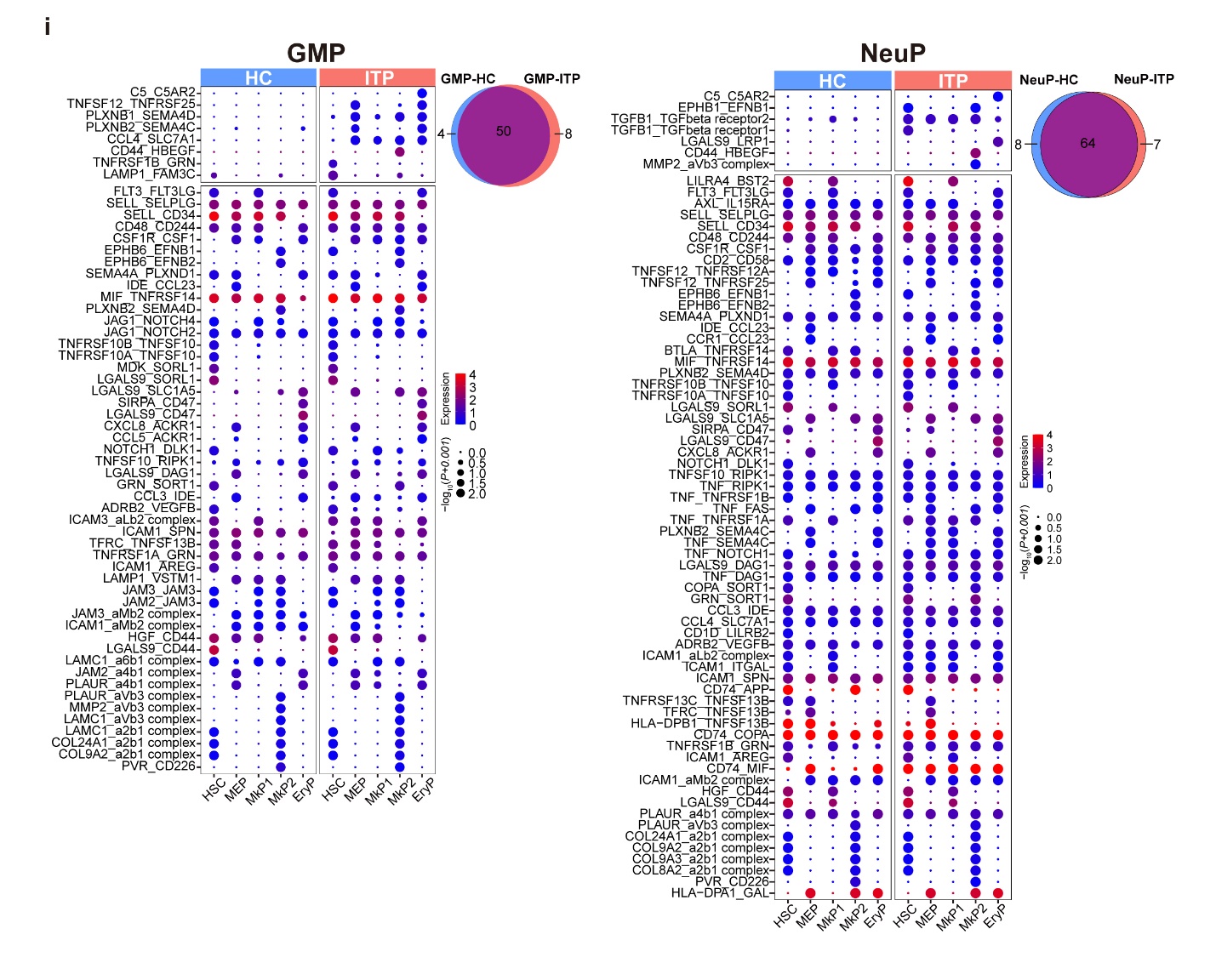


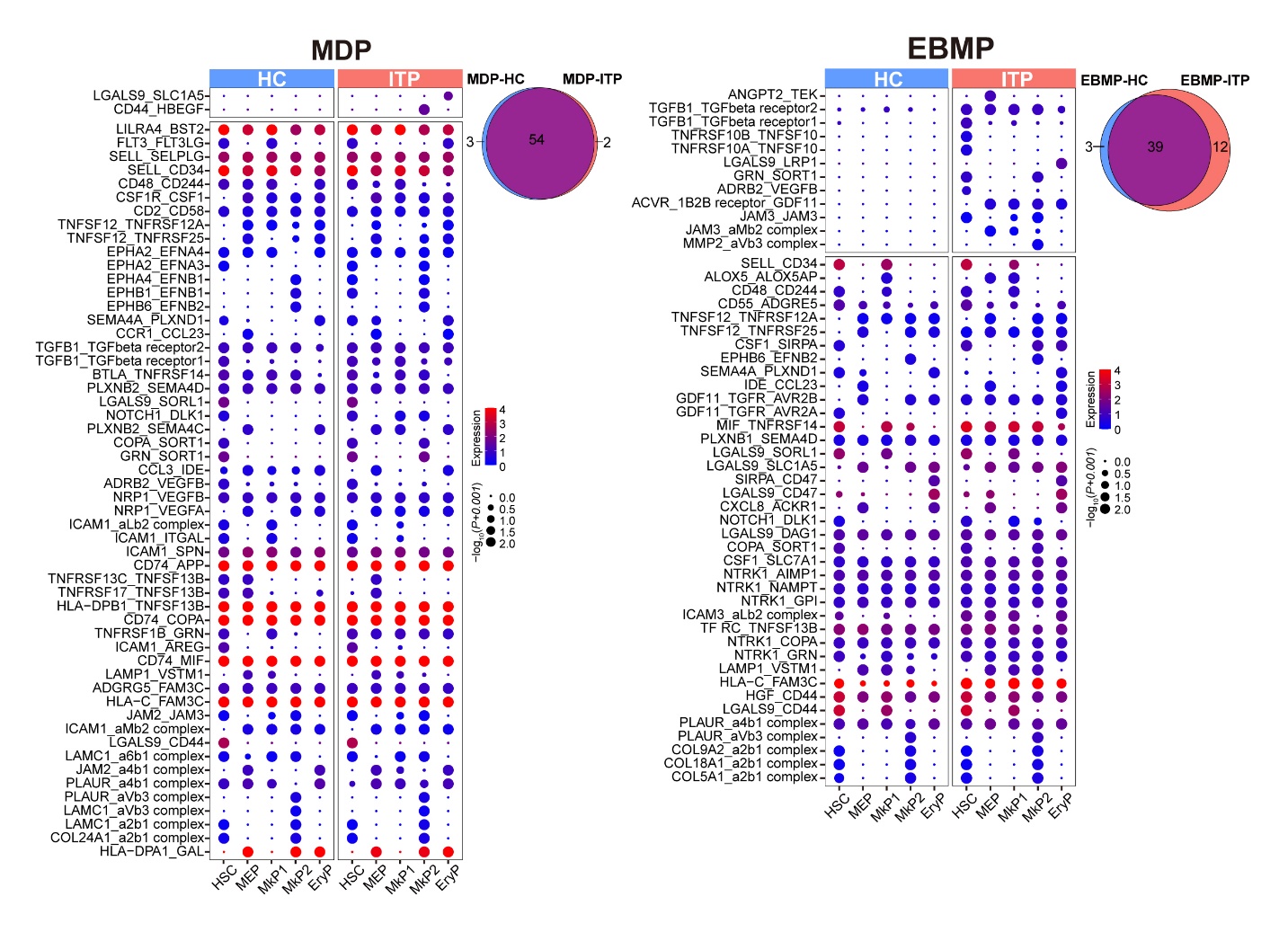


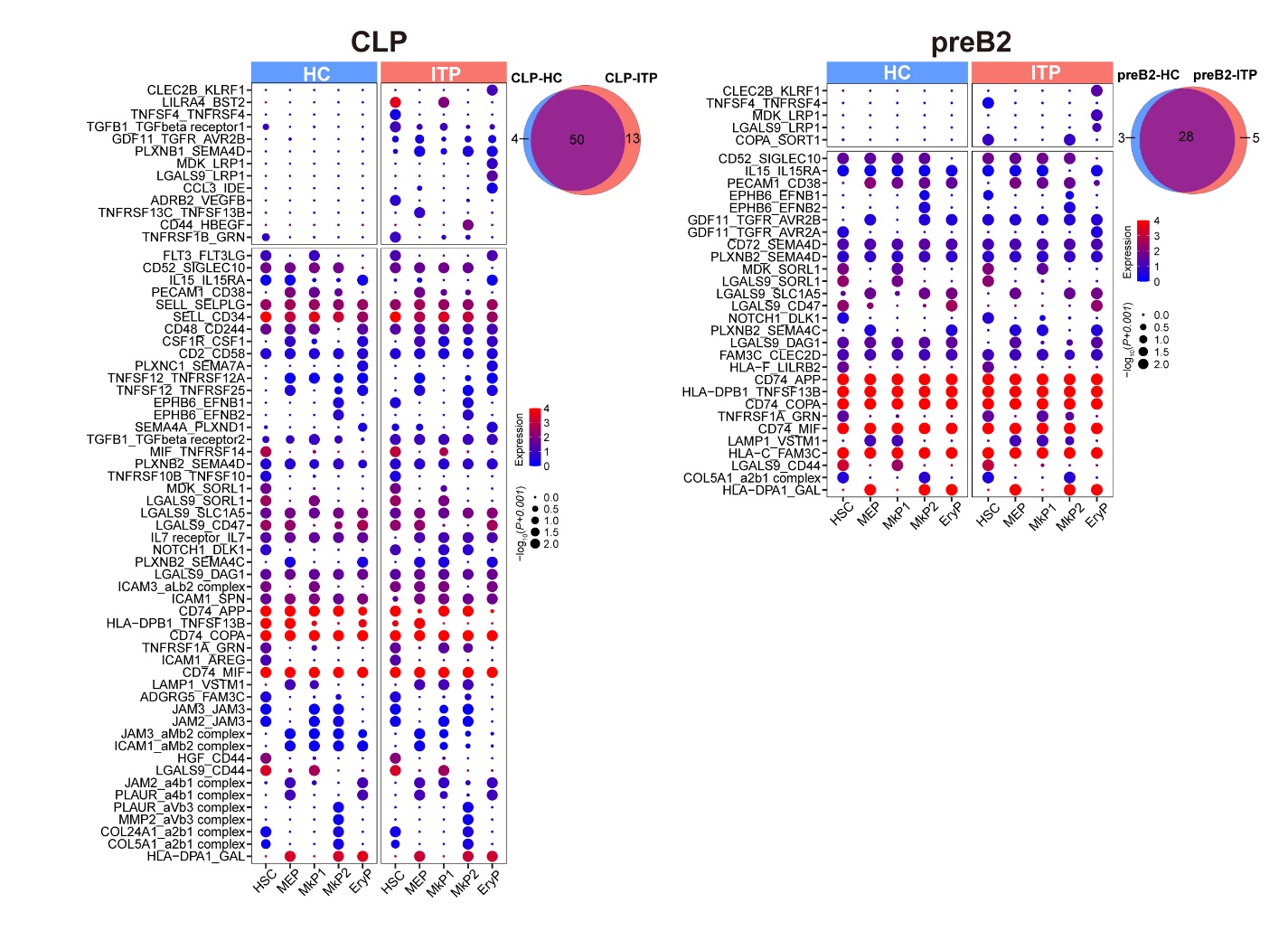


**Supplemental Fig. 4 Transcriptional changes of preB1 and NK/Tp in ITP and molecular interactions among HSPC subsets, related to Fig. 4.** (a) Correlation between ITP and HC transcriptomes in preB1 (left). Each axis represents the mean expression level in the HSPC subset and each point represent a single gene. Red points represent significantly upregulated genes in ITP, blue points represent significantly downregulated genes in ITP, and grey points represent non-DEGs. A log-transformed fold change absolute value greater than 0.25, the minimum percentage greater than 0.25, and adjusted p value less than 0.05 were used to define significance. Representative GO: BP terms were relatively enriched in preB1 from ITP versus HC (right). (b) Correlation between ITP and HC transcriptomes in NK/Tp (left). Each axis represents the mean expression level in the HSPC subset and each point represent a single gene. Red points represent significantly upregulated genes in ITP, blue points represent significantly downregulated genes in ITP, and grey points represent non-DEGs. A log-transformed fold change absolute value greater than 0.25, the minimum percentage greater than 0.25, and adjusted p value less than 0.05 were used to define significance. Representative GO: BP terms were relatively enriched in NK/Tp from ITP versus HC (right). (c) GSEA plots showing pathways enriched in preB1 from ITP versus HC. NES, normalized enrichment score; NOM p-value, nominal p-value; FDR q-value, false discovery rate q-value. (d) GSEA plots showing pathways enriched in NK/Tp from ITP versus HC. NES, normalized enrichment score; NOM p-value, nominal p-value; FDR q-value, false discovery rate q-value. (e) Bubble heat map of ligand-receptor interactions between preB1 and Mk/Ery-lineage cells. Interaction pairs with ITP (p < 0.05) were selected. ITP and HC are presented separately. Dot size indicates logarithmic transformed p values (permutation test). Color indicates the scaled mean expression levels of ligand and receptor molecules in the corresponding cell subpopulations. The upper panels represent interaction pairs specifically in ITP (p ≥ 0.05 in HC). The lower panel represents interaction pairs specific for both ITP and HC (p < 0.05 in HC). (f) Heat map of the area under the curve (AUC) scores of TF motifs estimated per sample in preB1 using SCENIC. A log-transformed fold change value greater than 0.25, and adjusted p value less than 0.05 were used to define significantly differential expression TFs. Significant TF motifs shared by at least two ITP samples would be selected for visualization. (g) Bubble heat map of ligand-receptor interactions between NK/Tp and Mk/Ery-lineage cells. Interaction pairs with ITP (p < 0.05) were selected. ITP and HC are presented separately. Dot size indicates logarithmic transformed p values (permutation test). Color indicates the scaled mean expression levels of ligand and receptor molecules in the corresponding cell subpopulations. The upper panels represent interaction pairs specifically in ITP (p ≥ 0.05 in HC). The lower panel represents interaction pairs specific for both ITP and HC (p < 0.05 in HC). (h) Heat map of the area under the curve (AUC) scores of TF motifs estimated per sample in NK/Tp using SCENIC. A log-transformed fold change value greater than 0.25, and adjusted p value less than 0.05 were used to define significantly differential expression TFs. Significant TF motifs shared by at least two ITP samples would be selected for visualization. (i) Bubble heat map of ligand-receptor interactions between six immune-related progenitor subsets (GMP, NeuP, MDP, EBMP, CLP, and preB2) and Mk/Ery-lineage cells. Interaction pairs with ITP (p < 0.05) were selected. ITP and HC are presented separately. Dot size indicates logarithmic transformed p values (permutation test). Color indicates the scaled mean expression levels of ligand and receptor molecules in the corresponding cell subpopulations. The upper panel represents interaction pairs specifically in ITP (p ≥ 0.05 in HC). The lower panel represents interaction pairs specific for both ITP and HC (p < 0.05 in HC). Venn diagrams showing overlap of ligand-receptor interactions.

Figure. S5.


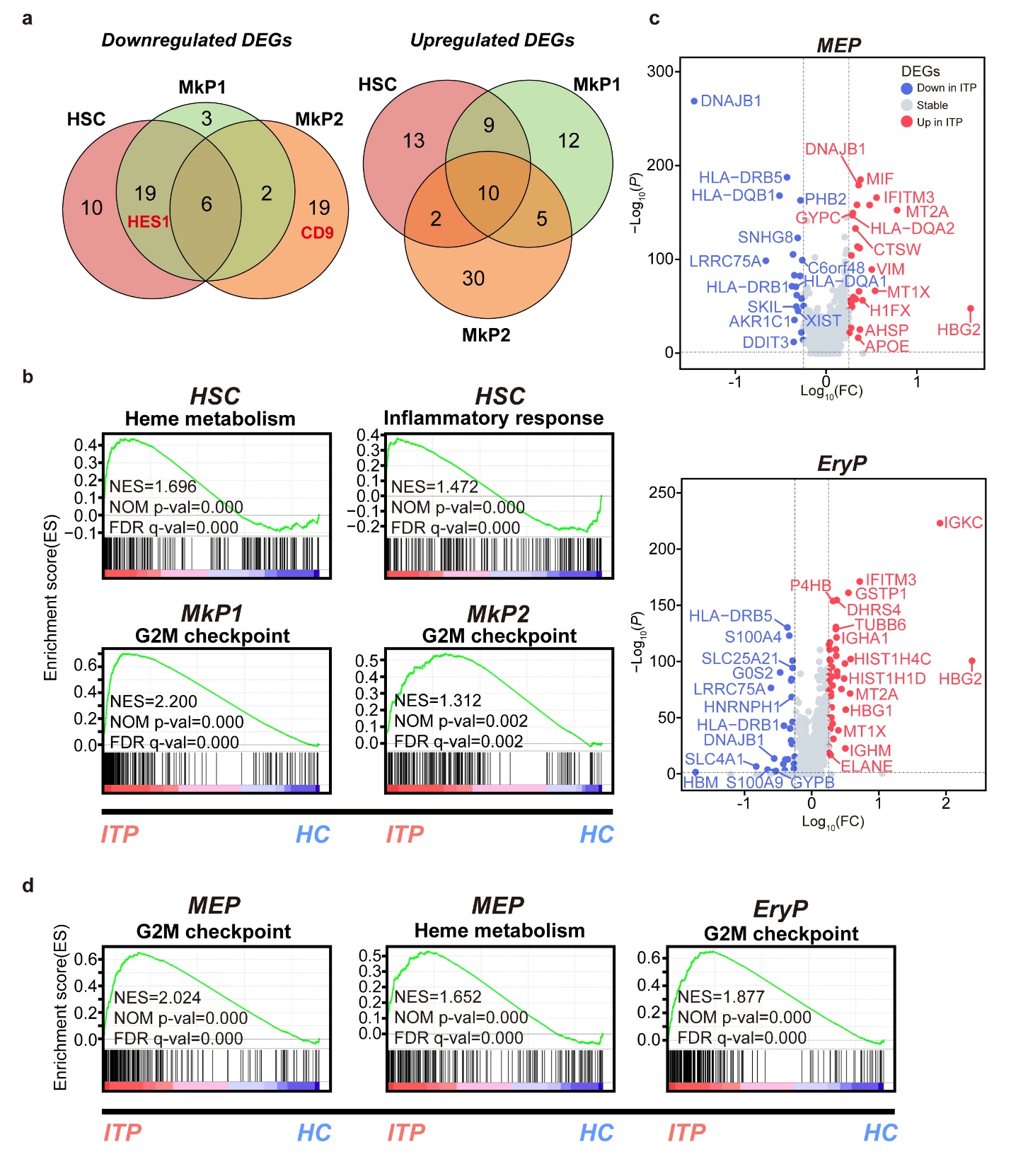


**Supplemental Fig. 5 Transcriptomic alterations of HSC, MEP, MkP1, MkP2, and EryP subsets in ITP, related to Fig. 5.** (a) Venn diagram showing the overlap of downregulated (left) and upregulated (right) DEGs in HSCs, MkP1, and MkP2 in ITP. Genes with an adjusted p value < 0.05, log-transformed fold change absolute value > 0.25, and minimum percentage > 0.25 were considered as DEGs. (b) GSEA plots showing pathways enriched in HSC, MkP1, and MkP2 from ITP versus HC. NES, normalized enrichment score; NOM p-value, nominal p-value; FDR q-value, false discovery rate q-value. (c) Volcano plots highlighting significant differences in gene expression between ITP and HC in MEP (upper) and EryP (lower). Red points represent significantly upregulated genes, blue points represent significantly downregulated genes, and grey points represent non-DEGs. Genes with an adjusted p value < 0.05, log-transformed fold change absolute value > 0.25, and minimum percentage > 0.25 were considered as DEGs. (d) GSEA plots showing pathways enriched in MEP and EryP from ITP versus HC. NES, normalized enrichment score; NOM p-value, nominal p-value; FDR q-value, false discovery rate q-value.

Figure. S6.


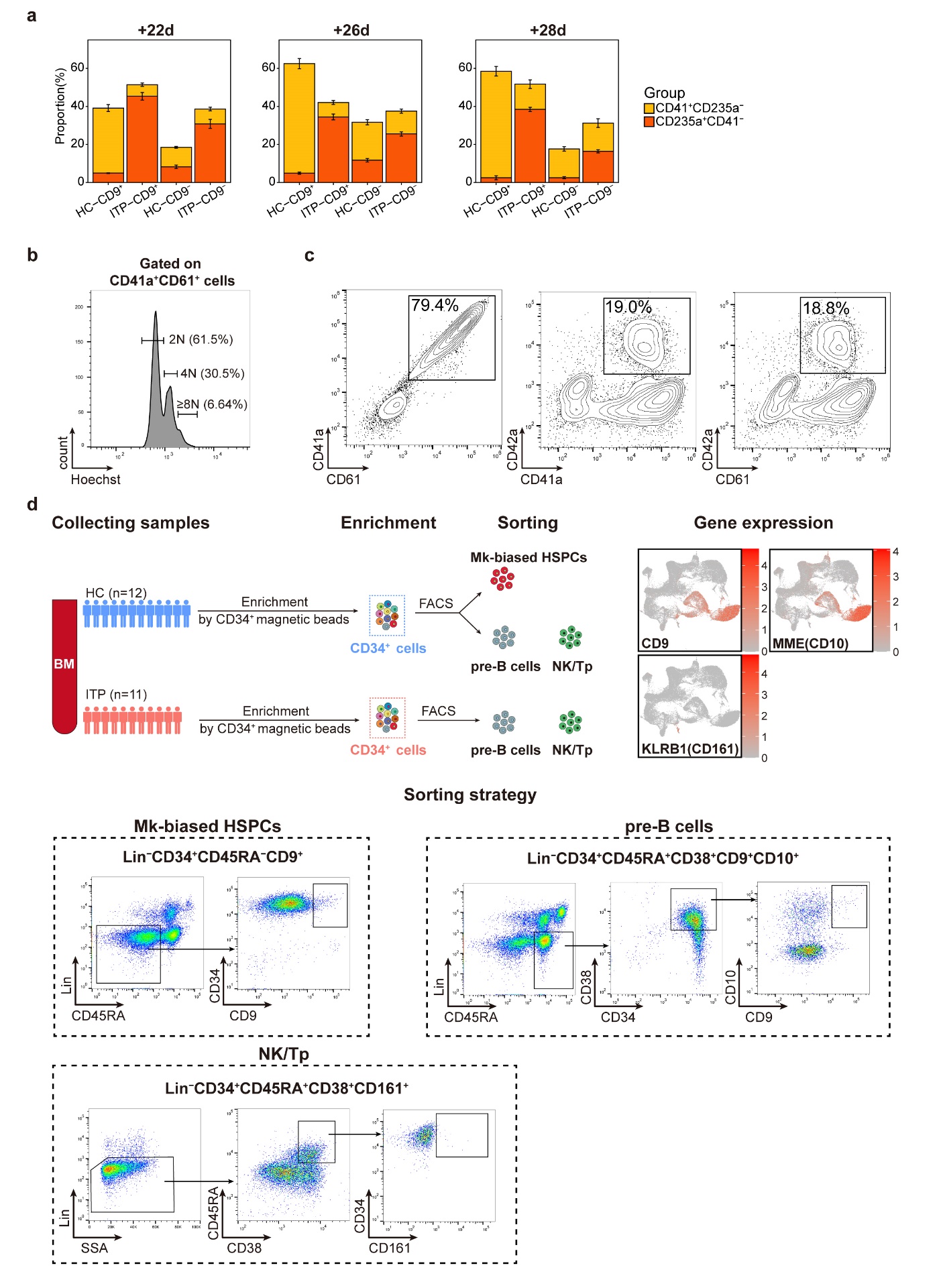


**Supplemental Fig. 6 Further investigation of CD9^+^Lin^−^CD34^+^CD45RA^−^ HSPCs, related to Fig. 5, 6.** (a) Stacked bar plot showing the proportion of CD41^+^CD235a^−^ and CD235a^+^CD41^−^ cells on days 22, 26, and 28 after culturing flow-sorted CD9^+^Lin^−^CD34^+^CD45RA^−^ HSPCs and CD9^−^Lin^−^CD34^+^CD45RA^−^ HSPCs. Error bars, mean ± S.E. ITP, n =5; HC, n = 4. (b) Ploidy analysis of CD41a^+^CD61^+^ cells cultured from CD9^+^Lin^−^CD34^+^CD45RA^−^ HSPCs. (c) The expression of CD41a, CD42a, and CD61 in cultures of CD9^+^Lin^−^CD34^+^CD45RA^−^ HSPCs from HC. (d) Schematic illustration of sample preparation for transwell co-culture systems. Related to Fig.6.

Figure. S7.


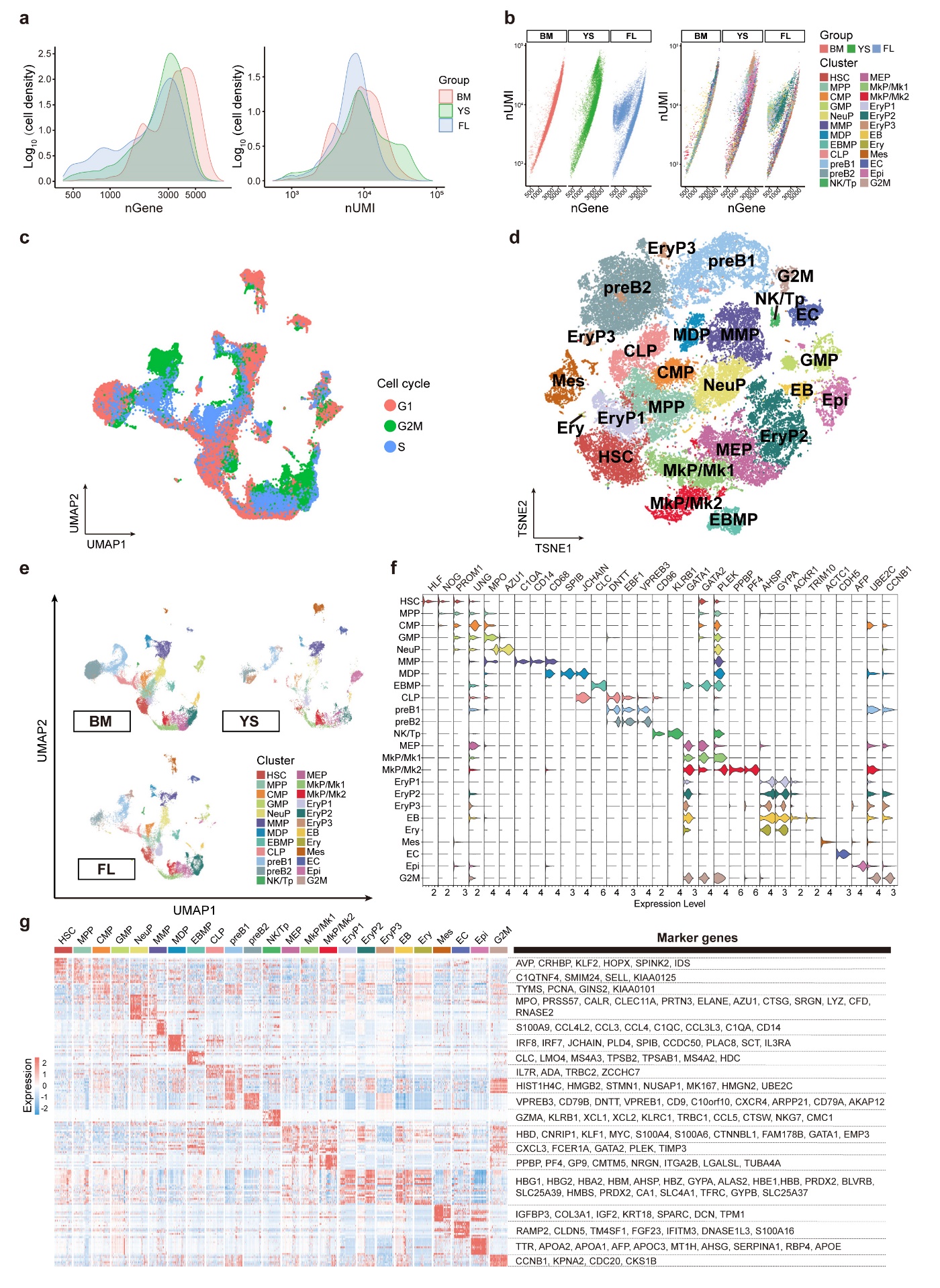


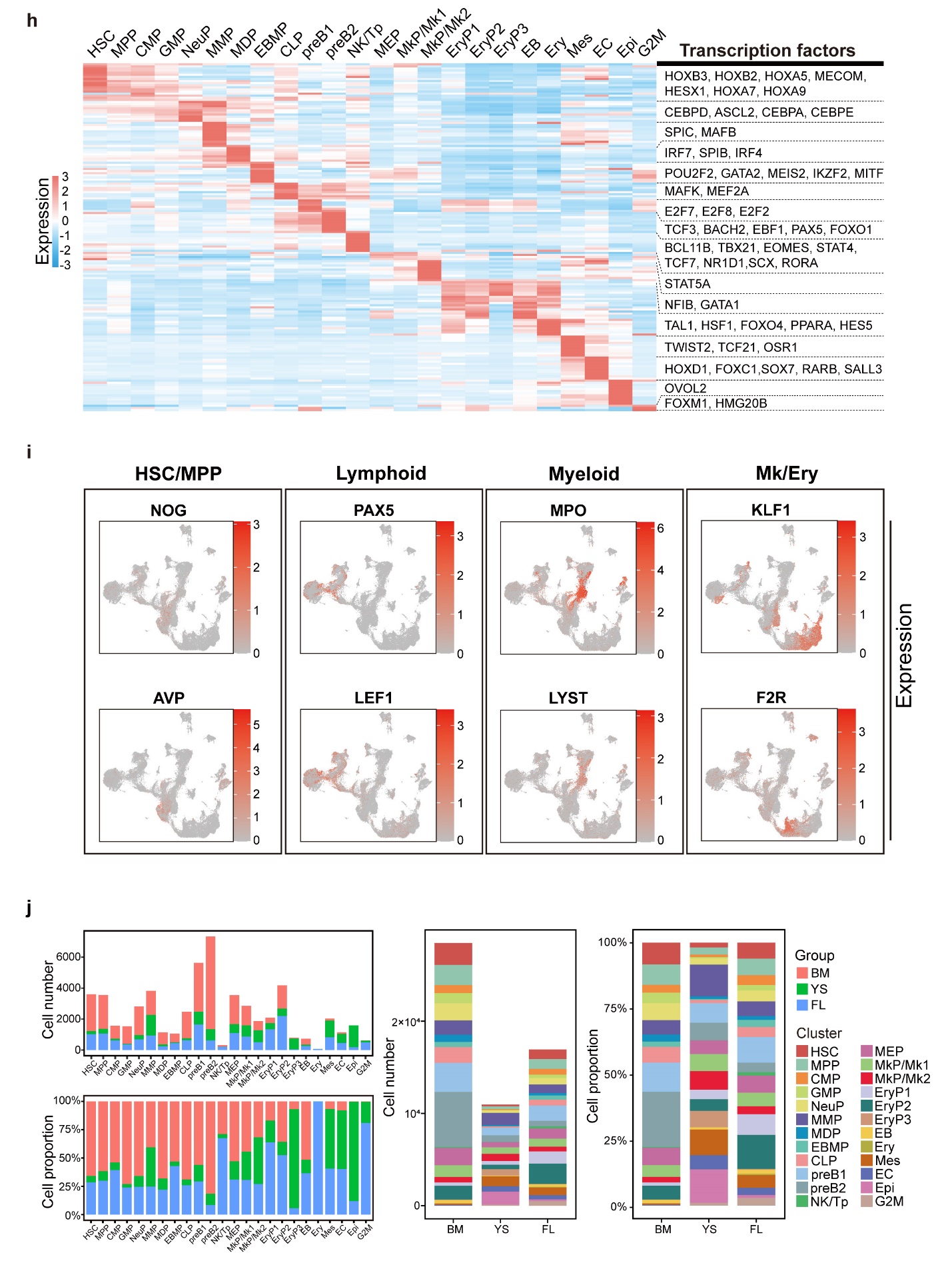


**Supplemental Fig. 7 Detailed characterization of cells came from 4 BM samples, 2 YS samples, and 2 FL samples, related to Fig. 7.** (a) Distribution of number of genes and UMIs detected per cell after filtering. (b) Scatter plot showing the linear correlation between the number of genes and the number of UMIs detected. Colors indicate groups (left) and cell types (right). (c) UMAP plot of scRNA-seq profiles. Each dot represents a cell and is colored based on the cell-cycle phase predicted by Seurat. (d) Cell clusters visualized using t-SNE. Colors indicate cell types. Each dot represents one cell. (e) UMAP plot showing cell clusters. Colors indicate cell types. Three sample sources are shown separately. (f) Violin plots showing the expression of specific marker genes in each cell cluster. Colors represent the cell clusters. (g) Heat map showing the scaled expression of top 10 marker genes in each cell cluster. Vital marker genes are highlighted on the right. (h) Top 10 differentially expressed TFs in each cell cluster. The vital TFs related to differentiation are listed on the right. (i) UMAP plots displaying the expression of canonical marker genes during hematopoietic development. (j) Stacked barplots show the proportions and numbers of cells from different sample sources in each cell cluster (left), and the proportions and numbers of annotated cell types in different sample sources (right).

Figure. S8.


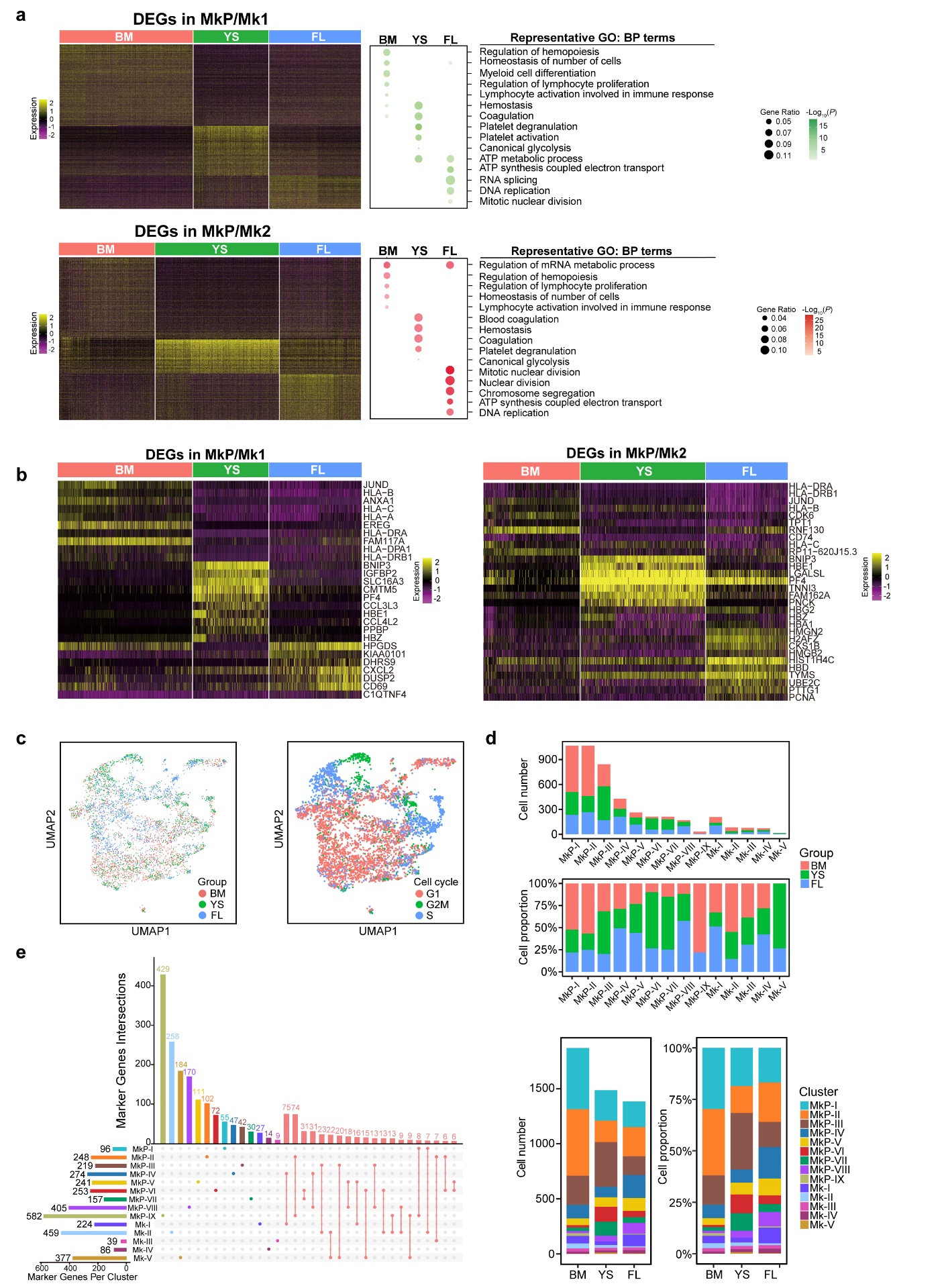


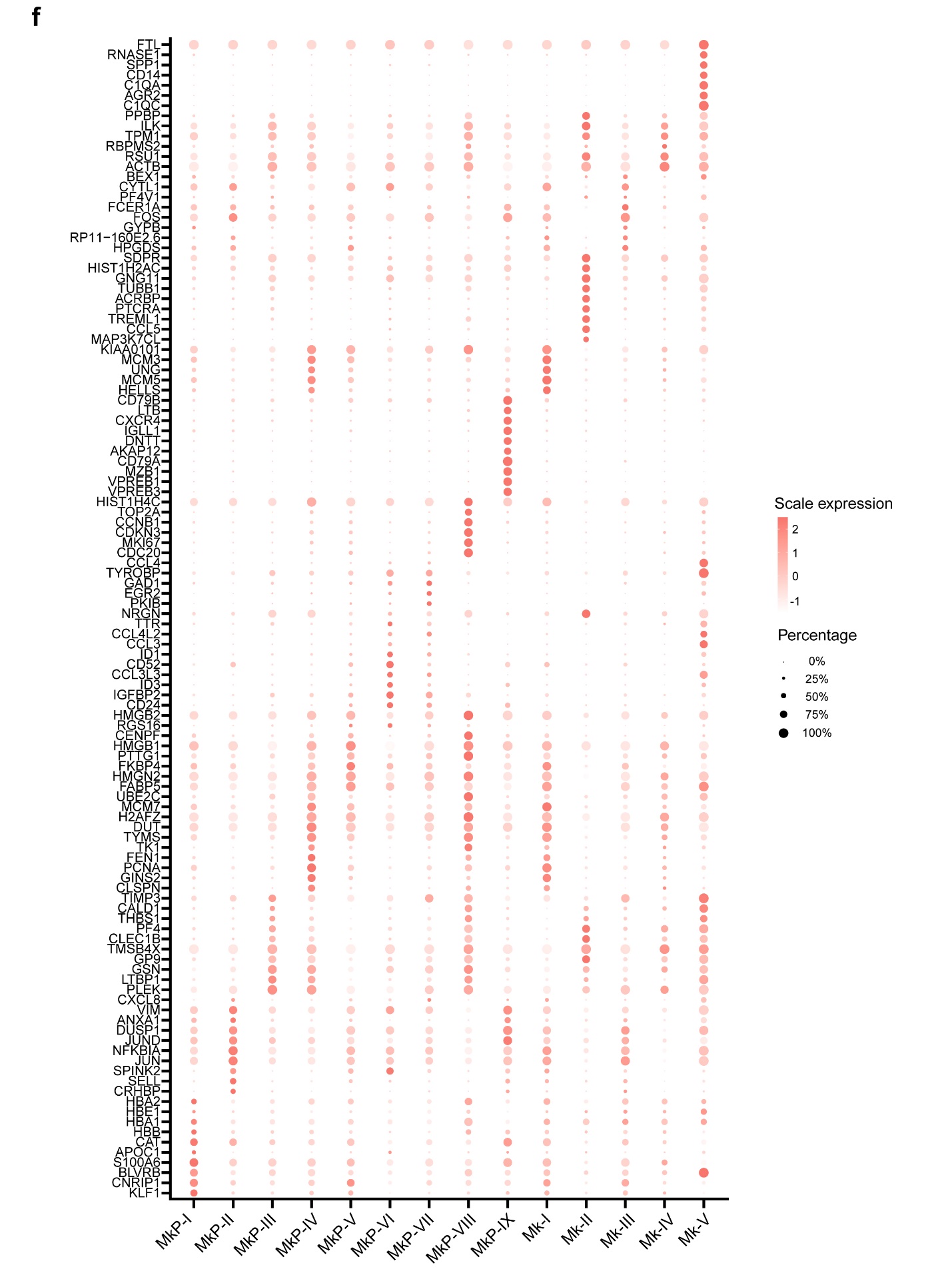


**Supplemental Fig. 8 Detailed analysis of MkP/Mk populations, related to Fig. 7.** (a) Left: Heat map showing the expression of DEGs in distinct sources of MkP/Mk1 (upper) and MkP/Mk2 (lower). Right: representative GO: BP terms. (b) Heat map of the top 10 significantly DEGs in distinct sources of MkP/Mk1 (left) and MkP/Mk2 (right). (c) UMAP plot showing cell distribution. Each dot represents one cell. Colors indicate groups (left) and cell-cycle phases (right). (d) Stacked barplots show the proportions and numbers of cells from different sample sources in each cell cluster (upper), and the proportions and numbers of annotated cell types in different sample sources (lower). (e) Comparative analysis of marker genes in each MkP/Mk sub-cluster. The horizontal bars represent the total number of marker genes per sub-cluster. The vertical bars or intersections represent the number of genes that were significantly upregulated in one or more sub-clusters. The top 20 intersections are shown. Genes with an adjusted p value < 0.05, log-transformed fold change value > 0.25, and minimum percentage > 0.25 were considered as significantly upregulated genes. (f) Dot plots showing the scaled expression level of the top 10 significantly DEGs in the MkP/Mk sub-clusters. Colors represent the scaled expression and size indicates the proportion of gene-expressing cells.

Figure. S9.


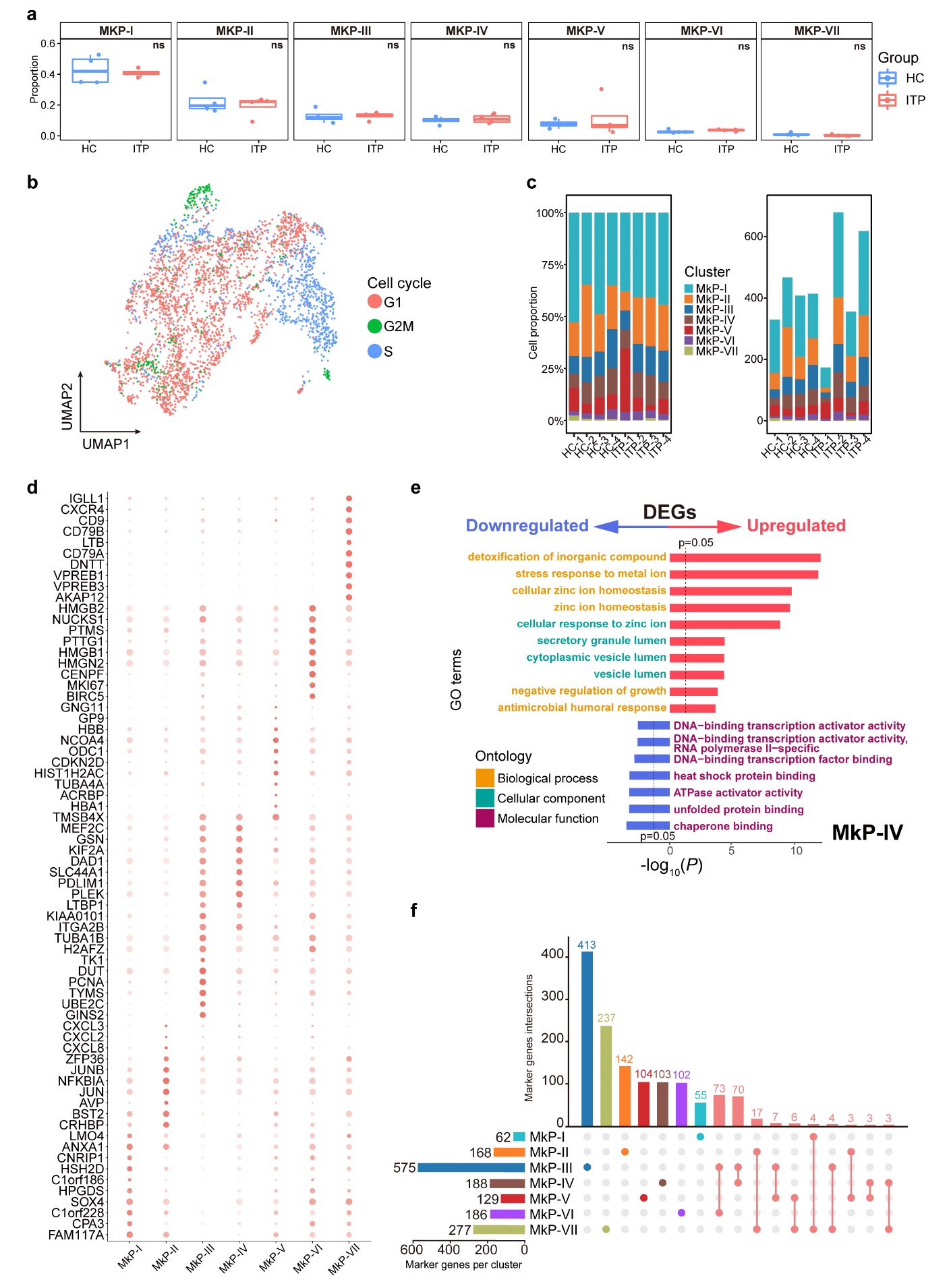


**Supplemental Fig. 9. Detailed analysis of MkP populations in BM, related to Fig. 8, 9.** (a) Boxplot showing the fraction of each MkP sub-cluster in ITP (blue) and HC (red) samples. The p values were calculated using two-tailed Student’s t-test; ns, not significant. (b) UMAP plot of scRNA-seq profiles. Each dot represents a cell and is colored based on the cell-cycle phase predicted by Seurat. (c) Stacked barplots show the proportions of annotated sub-clusters in different sample sources (left), and the numbers of annotated sub-clusters in different sample sources (right). (d) Dot plots showing the scaled expression level of the top 10 significantly DEGs in the MkP sub-clusters. Colors represent the scaled expression and size indicates the proportion of gene-expressing cells. (e) Two-sided bar graph showing the top 10 enriched upregulated and downregulated GO terms in MkP-Ⅳ in ITP. (f) Comparative analysis of marker genes in each sub-cluster. The horizontal bars represent the total number of marker genes per sub-cluster. The vertical bars or intersections represent the number of genes that were significantly upregulated in one or more sub-clusters. The top ten intersections are shown. Genes with an adjusted p value < 0.05, log-transformed fold change value > 0.25, and minimum percentage > 0.25 were considered as significantly upregulated genes.

Figure. S10.


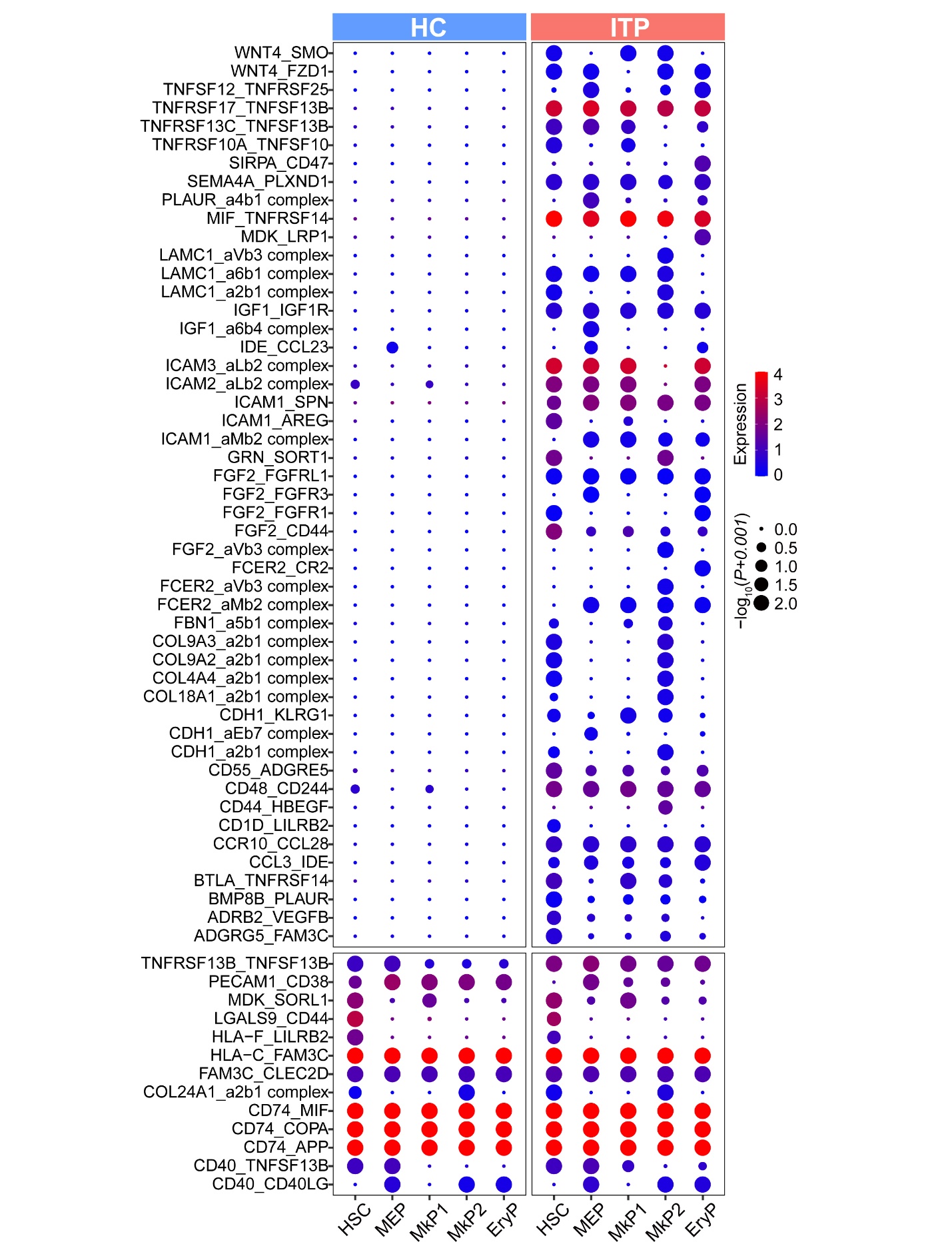


**Supplemental Fig. 10. The enlarged version of Fig. 4f.**

Figure. S11.


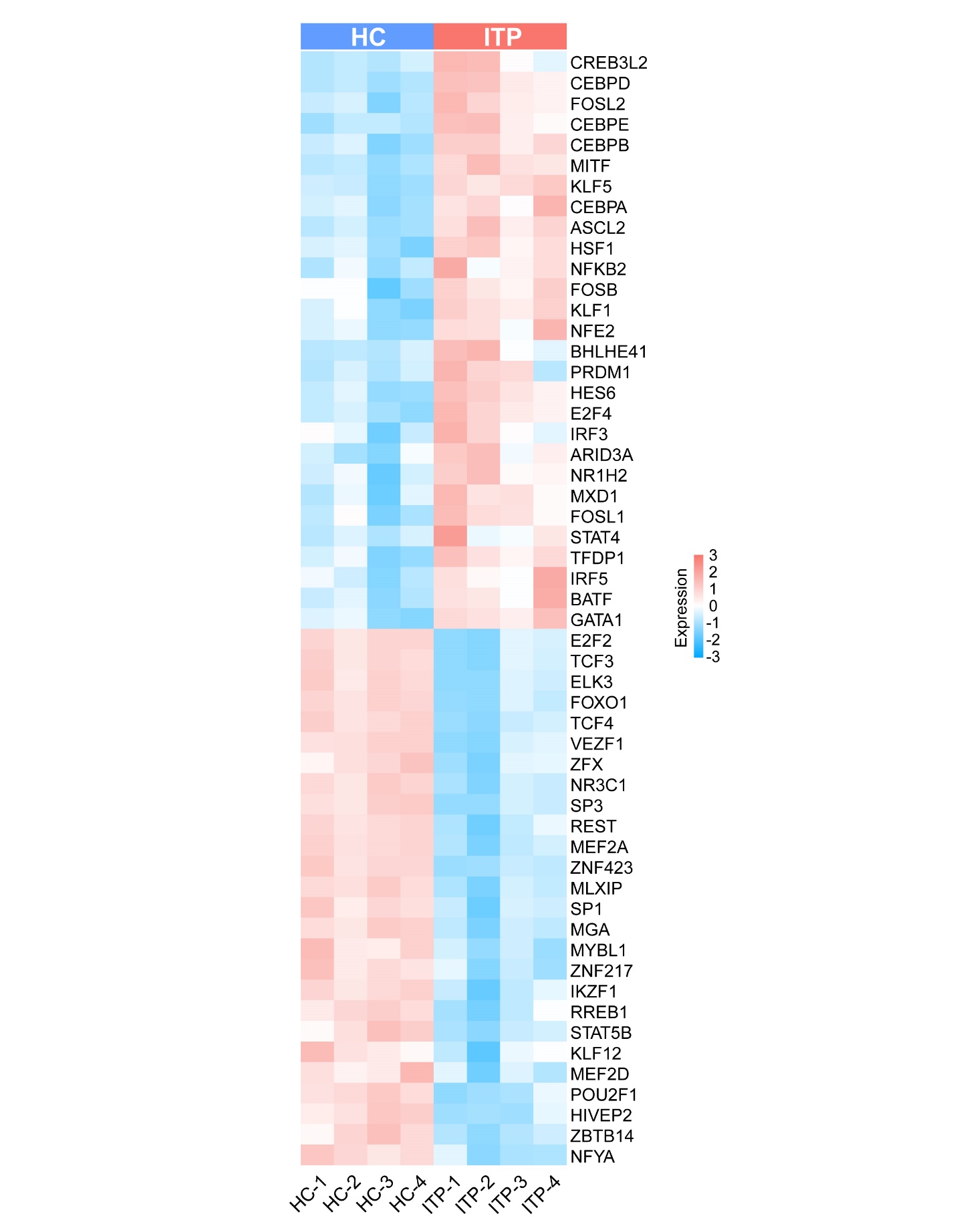


**Supplemental Fig. 11. The enlarged version of Fig. 4g.**

Figure. S12.


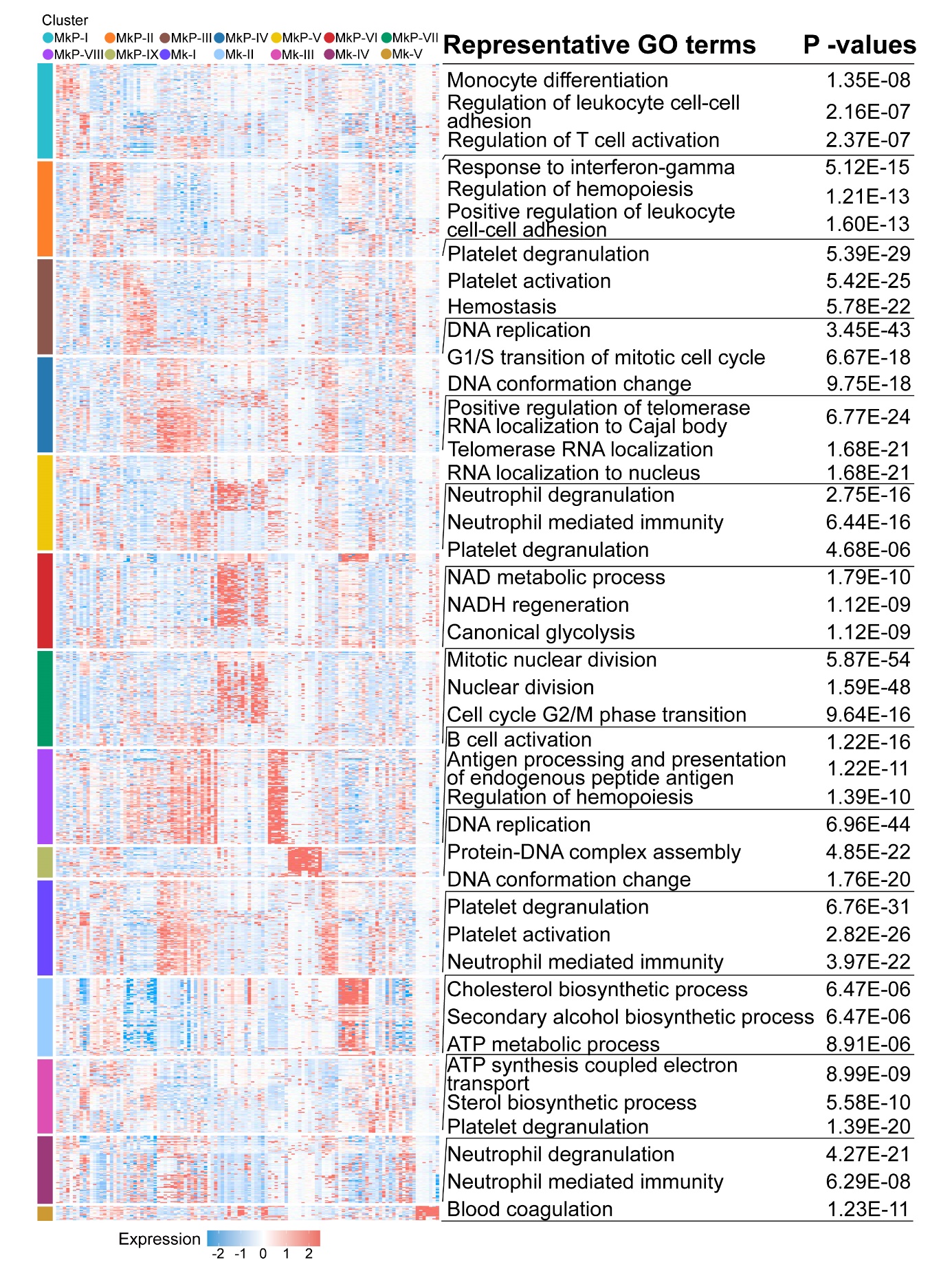


**Supplemental Fig. 12. The enlarged version of Fig. 7f.**

Figure. S13.


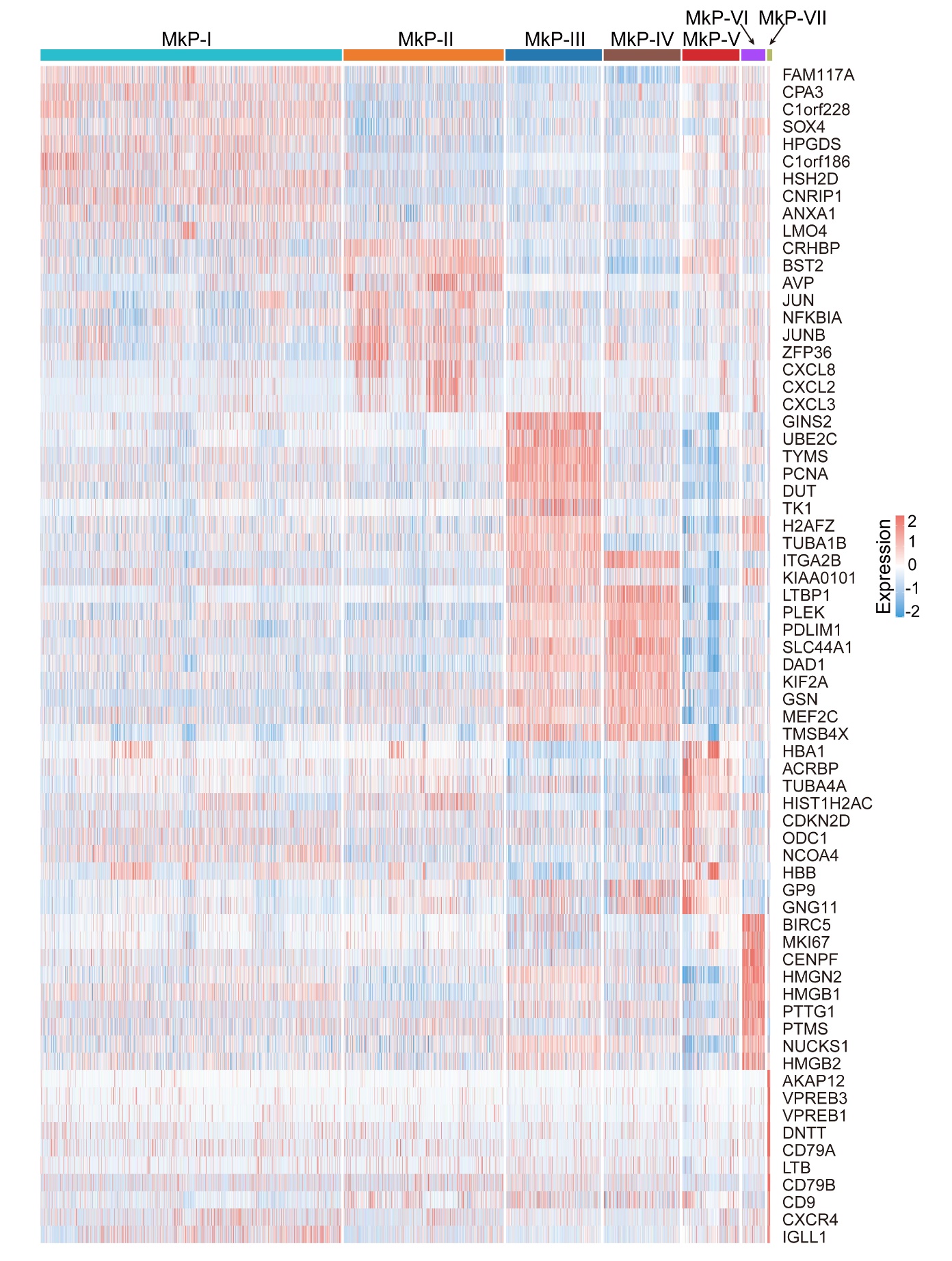


**Supplemental Fig. 13. The enlarged version of Fig. 8e.**

Figure. S14.


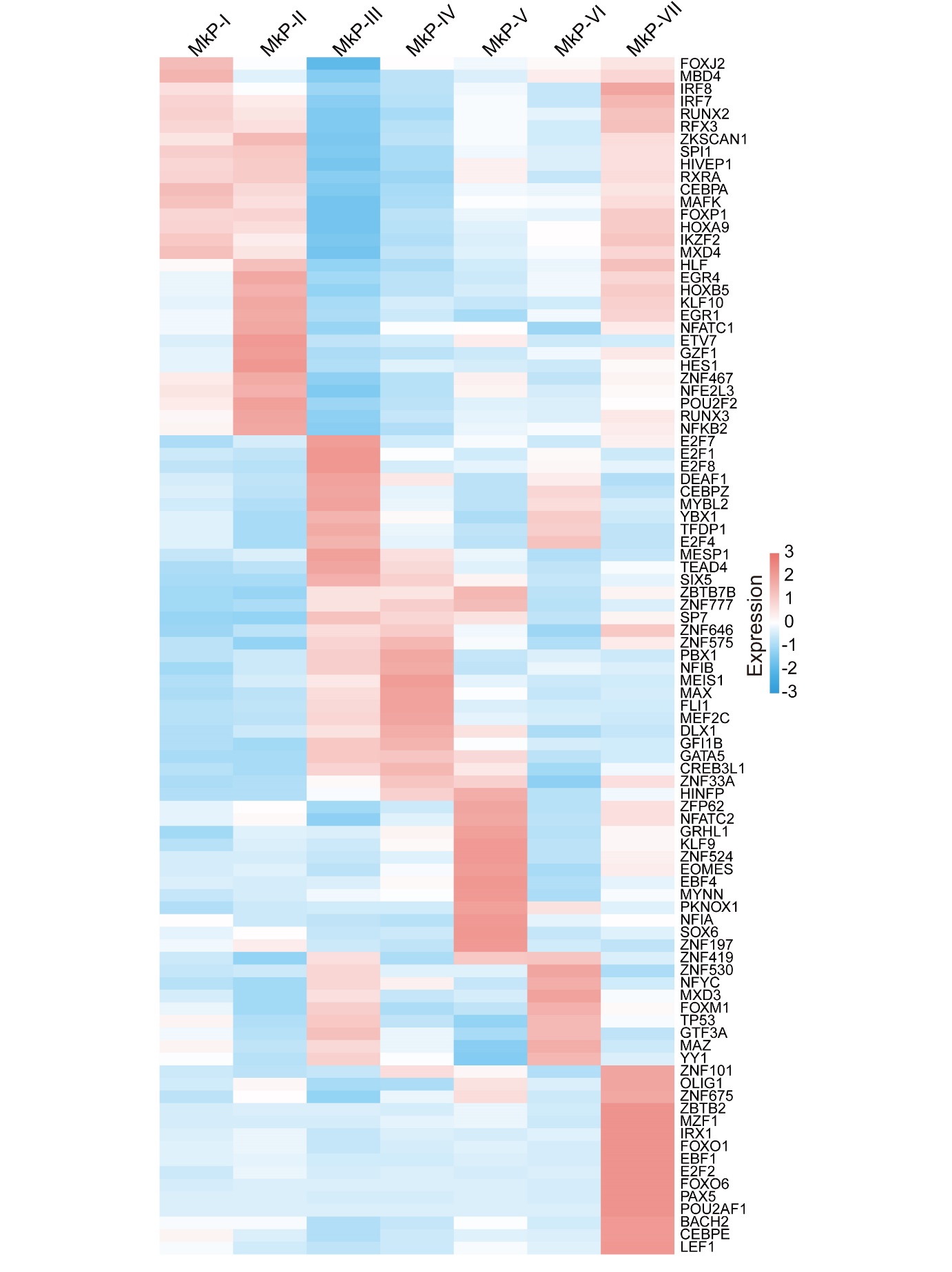


**Supplemental Fig. 14. The enlarged version of Fig. 8f.**
