# Supplementary figures and images for: Deciphering transcriptome alterations in bone marrow hematopoiesis at single-cell resolution in immune thrombocytopenia
Source: Signal Transduct Target Ther. 2022 Oct 7;7:347. doi: 10.1038/s41392-022-01167-9 (PMC9537316; doi:10.1038/s41392-022-01167-9)

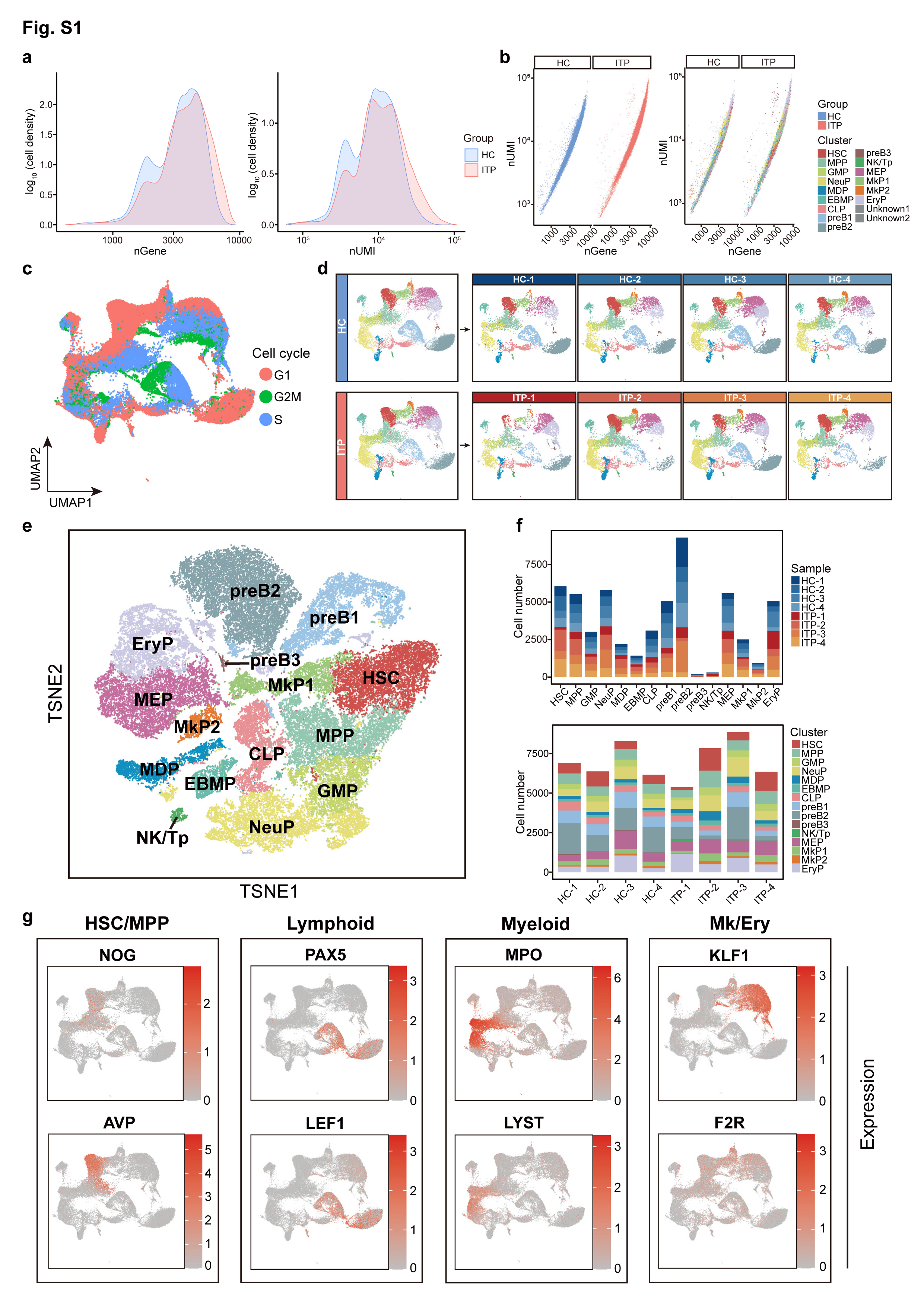

Supplement: Supplementary file 2 — Supplemental Fig. 1 [file 41392_2022_1167_MOESM2_ESM.jpg]

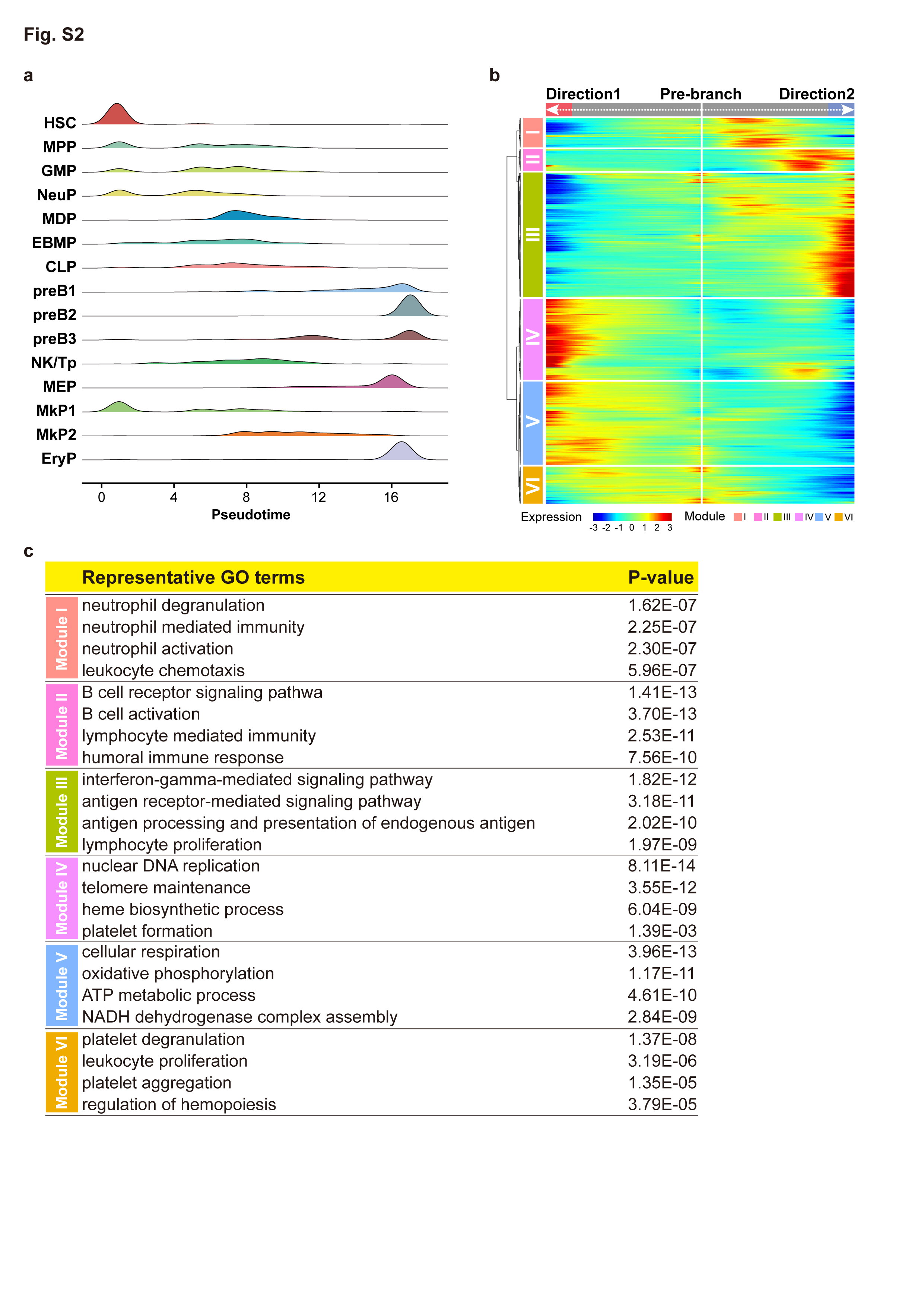

Supplement: Supplementary file 3 — Supplemental Fig. 2 [file 41392_2022_1167_MOESM3_ESM.jpg]

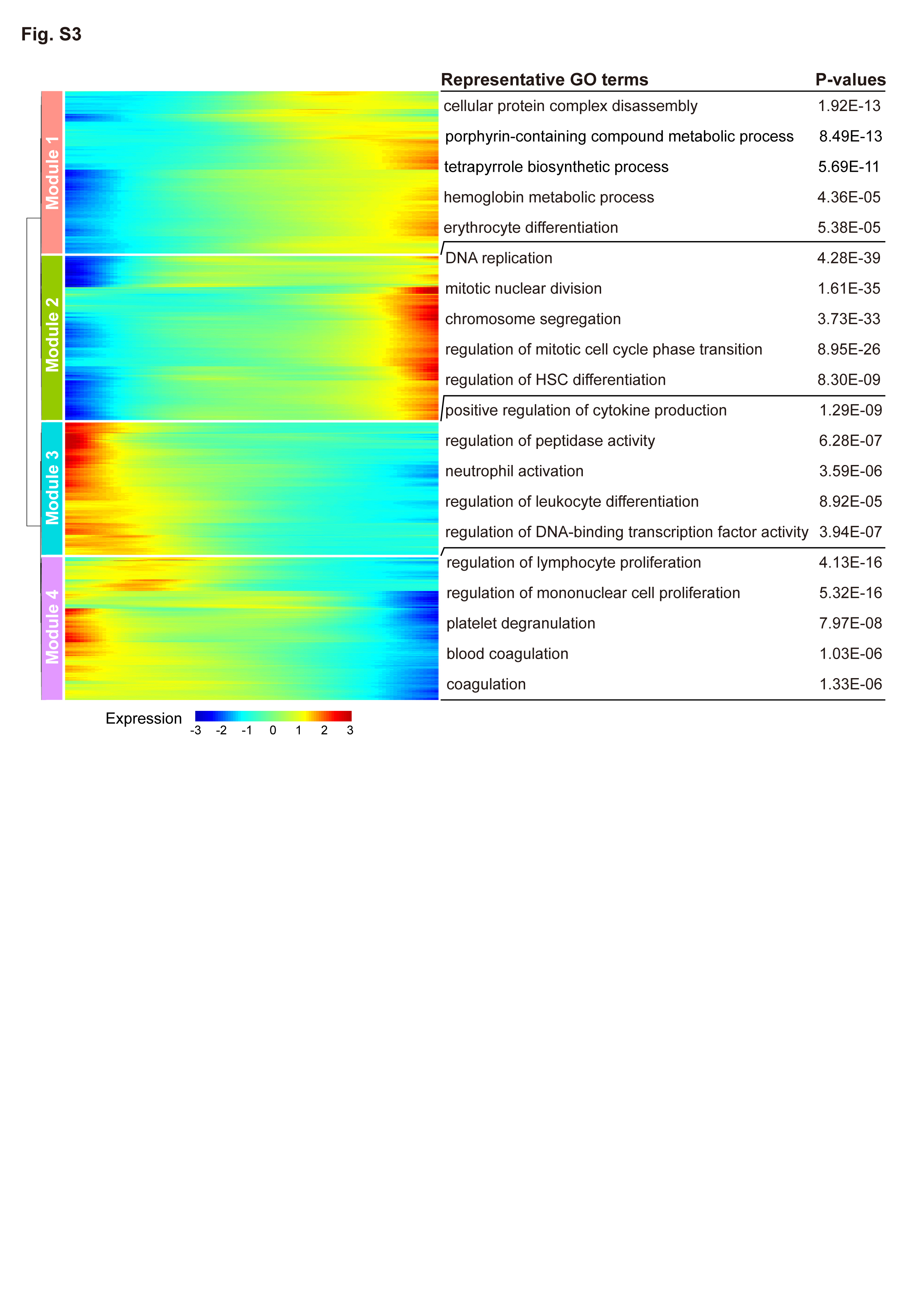

Supplement: Supplementary file 4 — Supplemental Fig. 3 [file 41392_2022_1167_MOESM4_ESM.jpg]

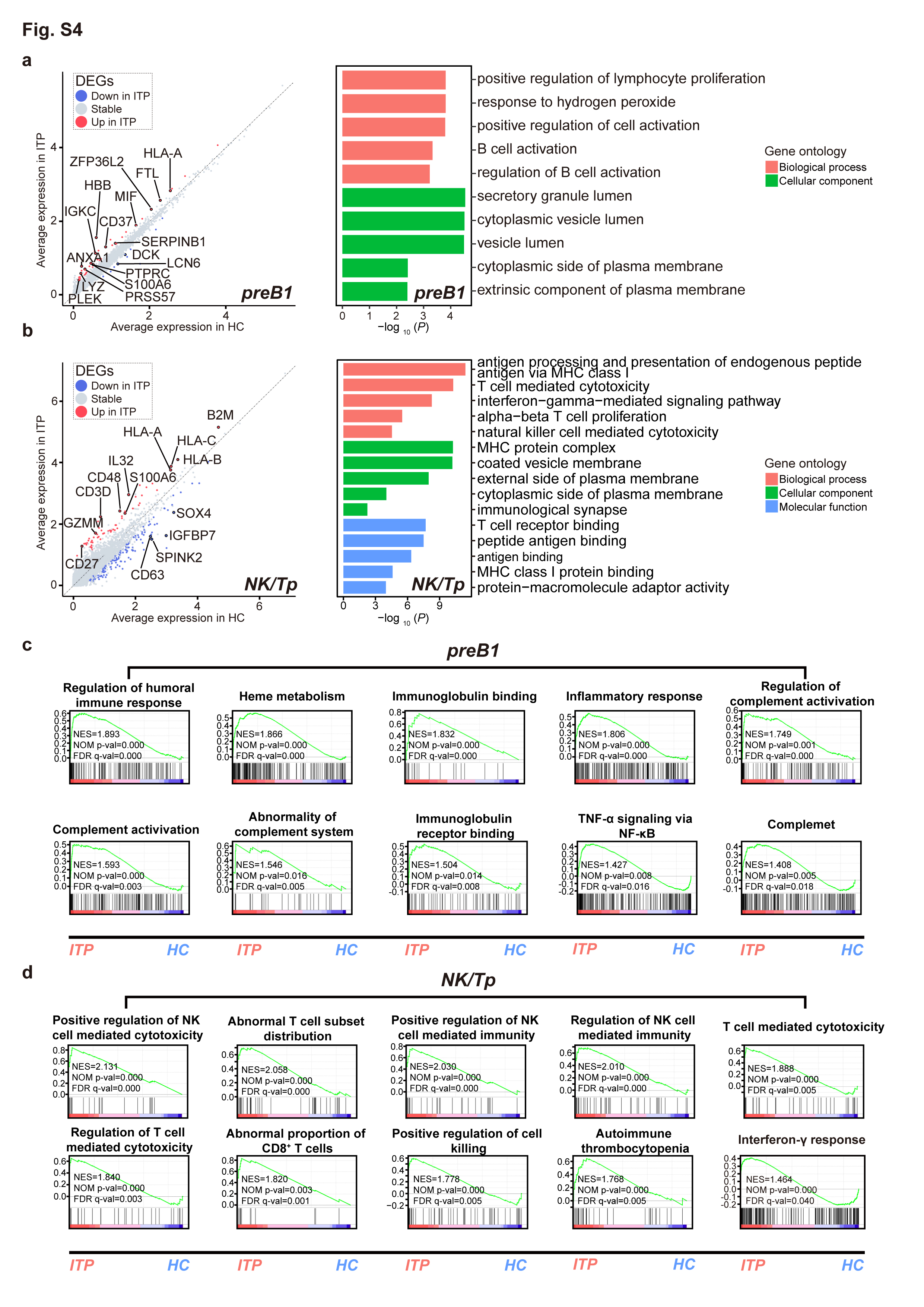

Supplement: Supplementary file 5 — Supplemental Fig. 4–1 [file 41392_2022_1167_MOESM5_ESM.jpg]

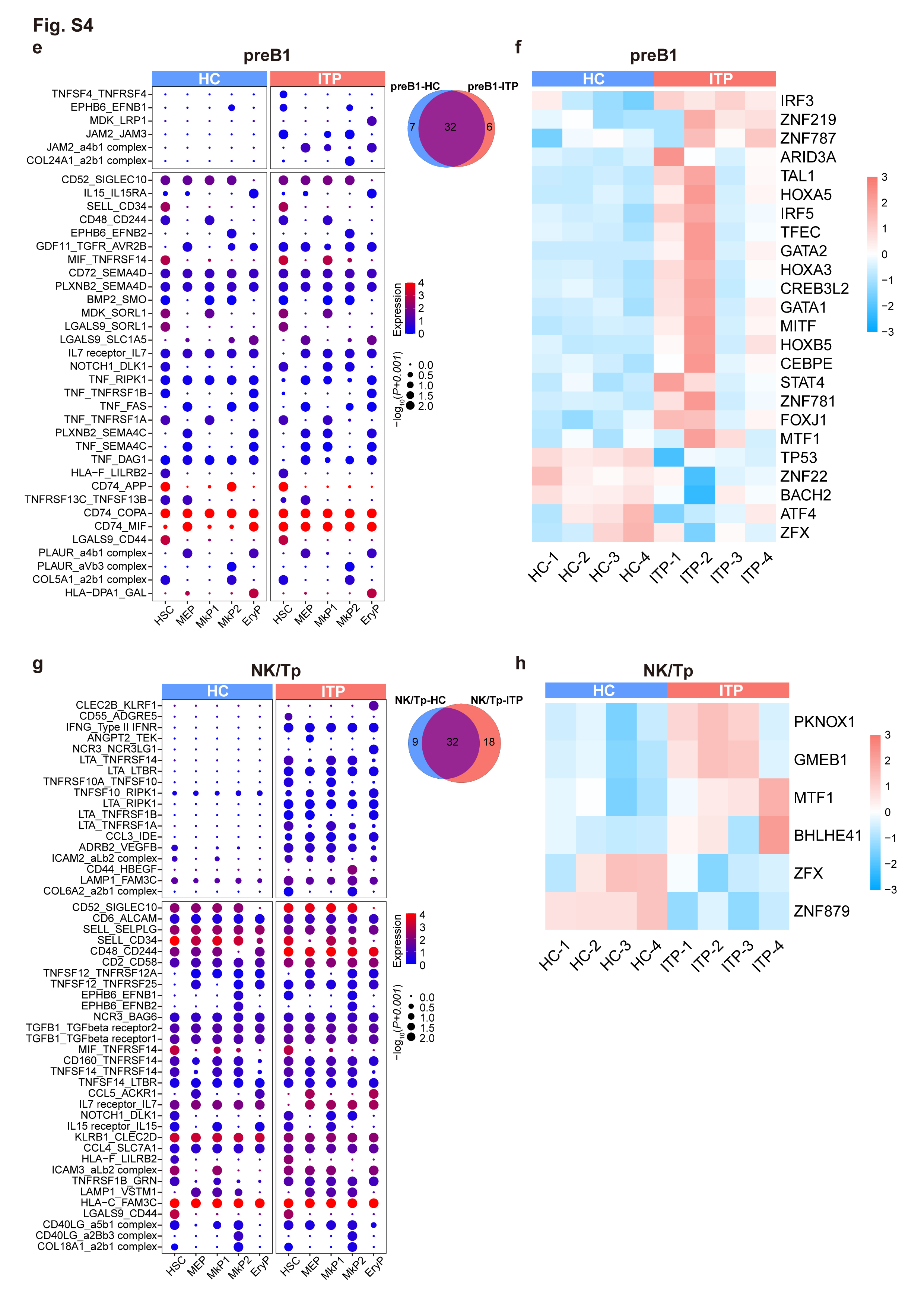

Supplement: Supplementary file 6 — Supplemental Fig. 4–2 [file 41392_2022_1167_MOESM6_ESM.jpg]

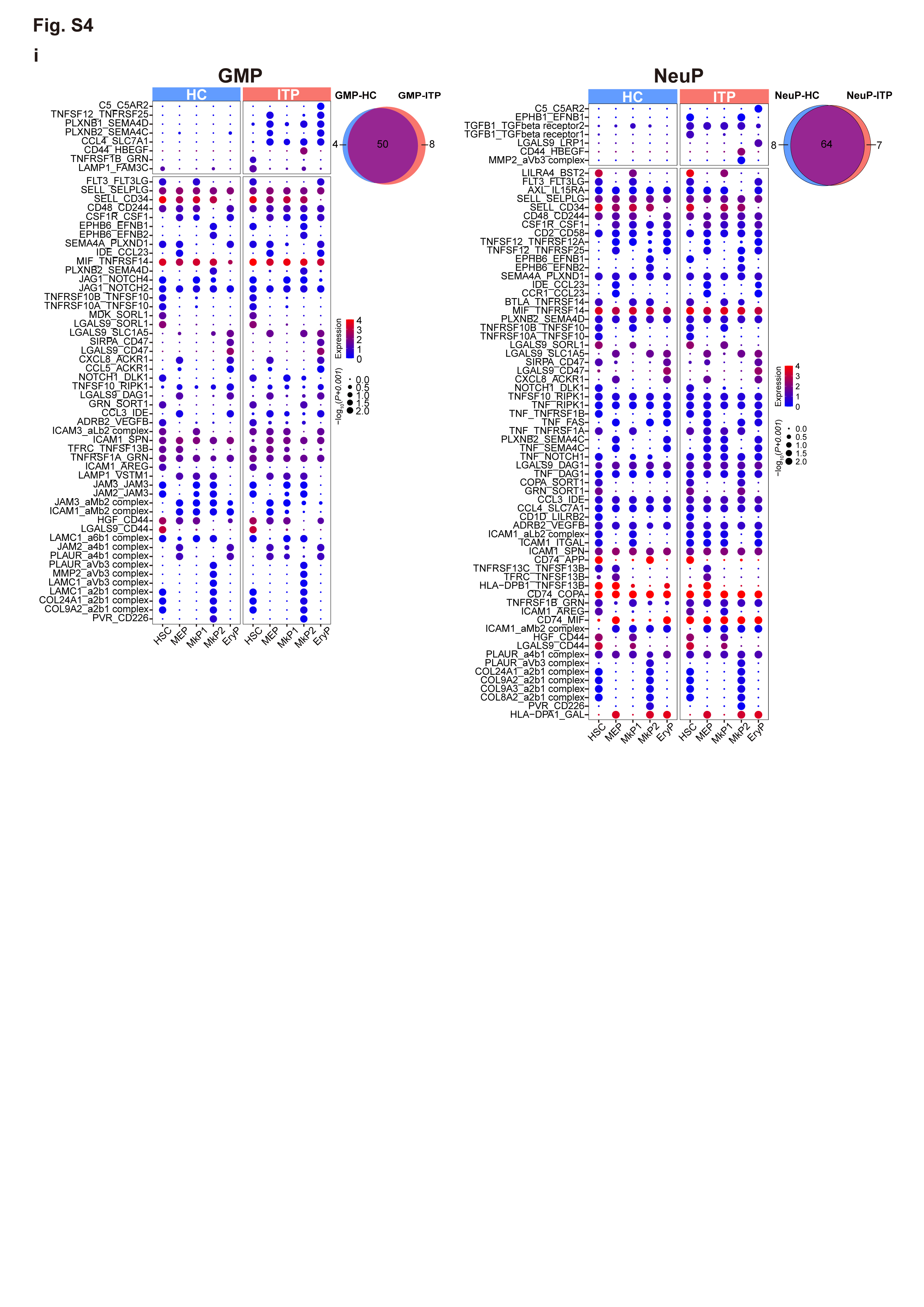

Supplement: Supplementary file 7 — Supplemental Fig. 4–3 [file 41392_2022_1167_MOESM7_ESM.jpg]

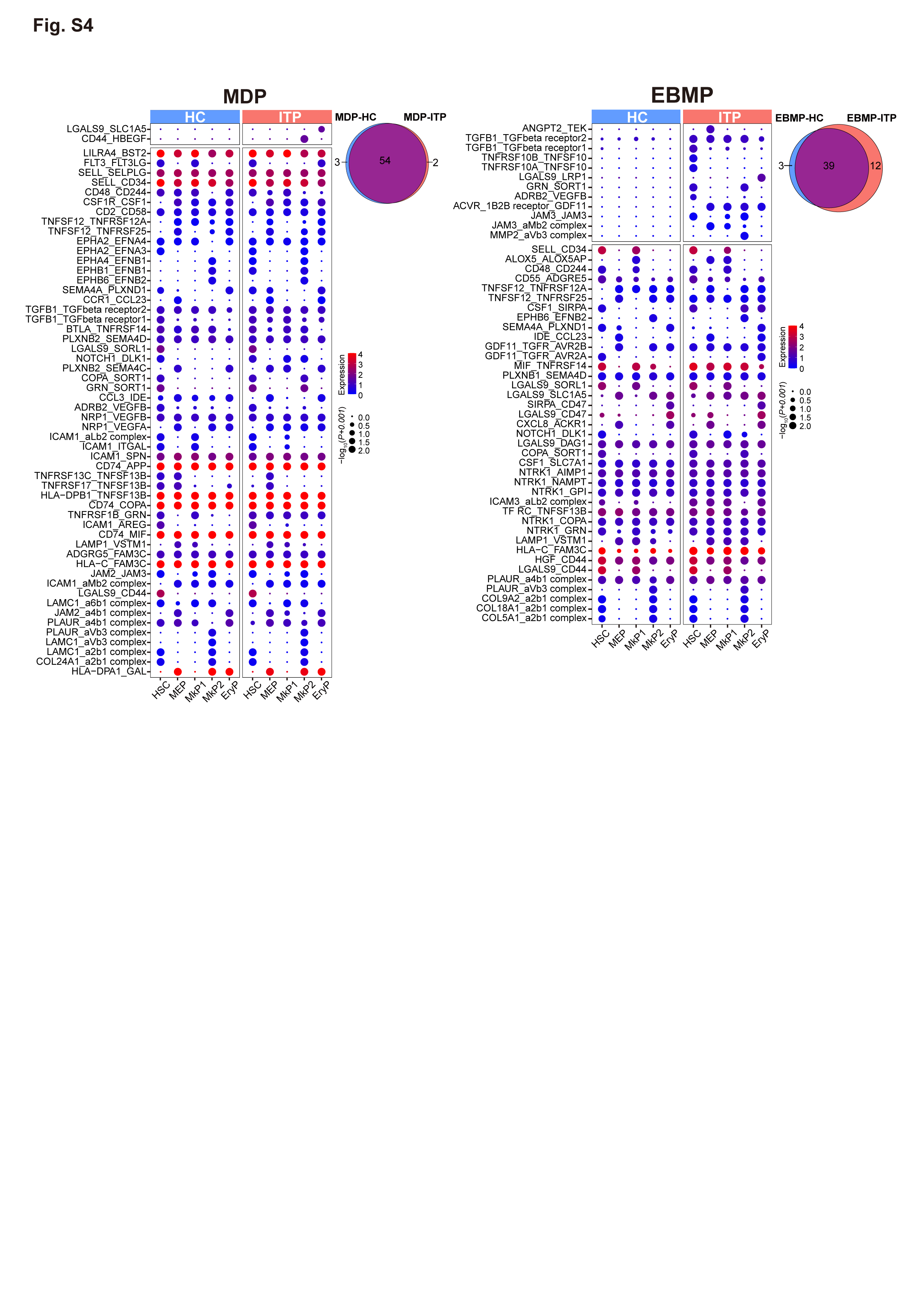

Supplement: Supplementary file 8 — Supplemental Fig. 4–4 [file 41392_2022_1167_MOESM8_ESM.jpg]

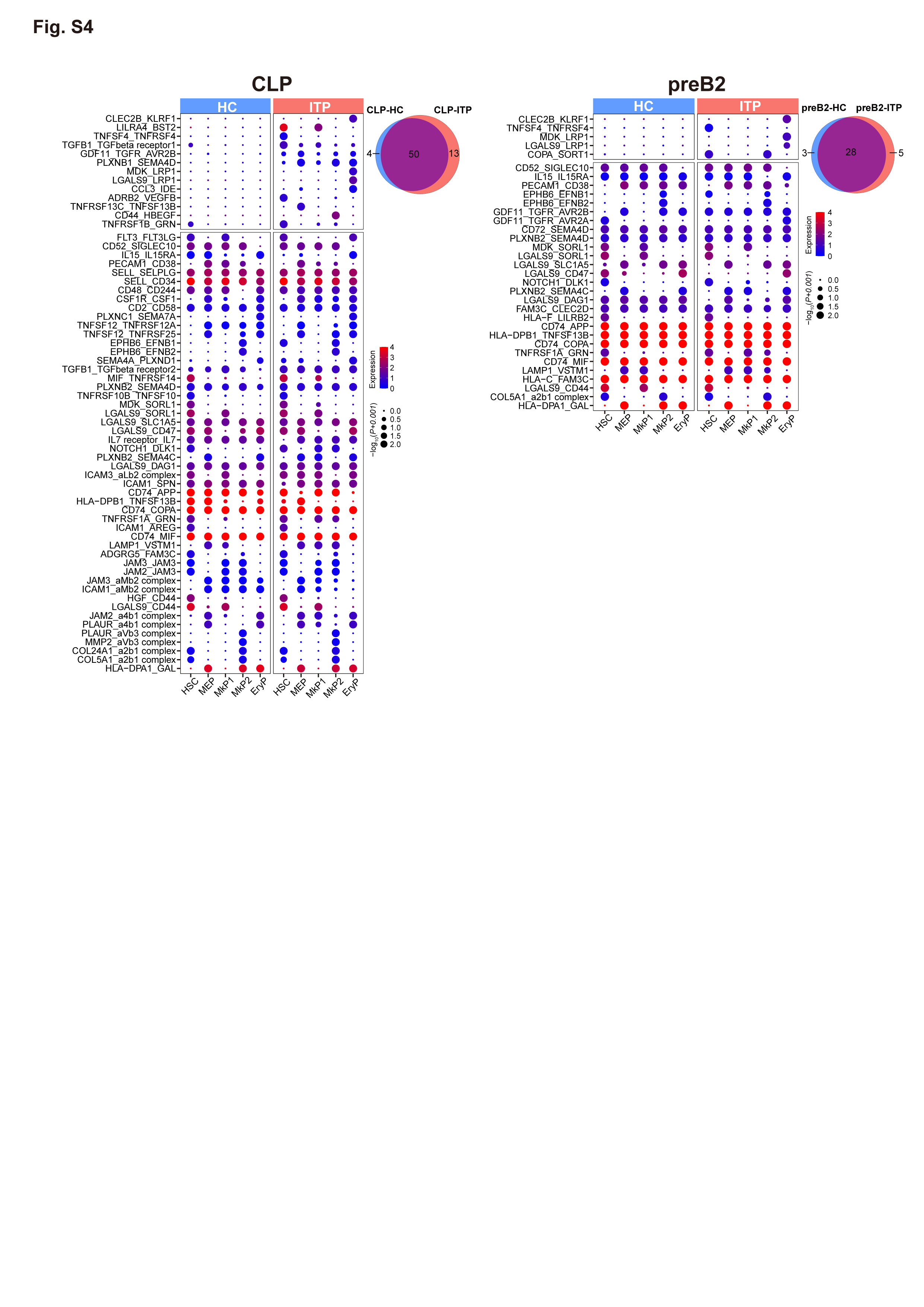

Supplement: Supplementary file 9 — Supplemental Fig. 4–5 [file 41392_2022_1167_MOESM9_ESM.jpg]

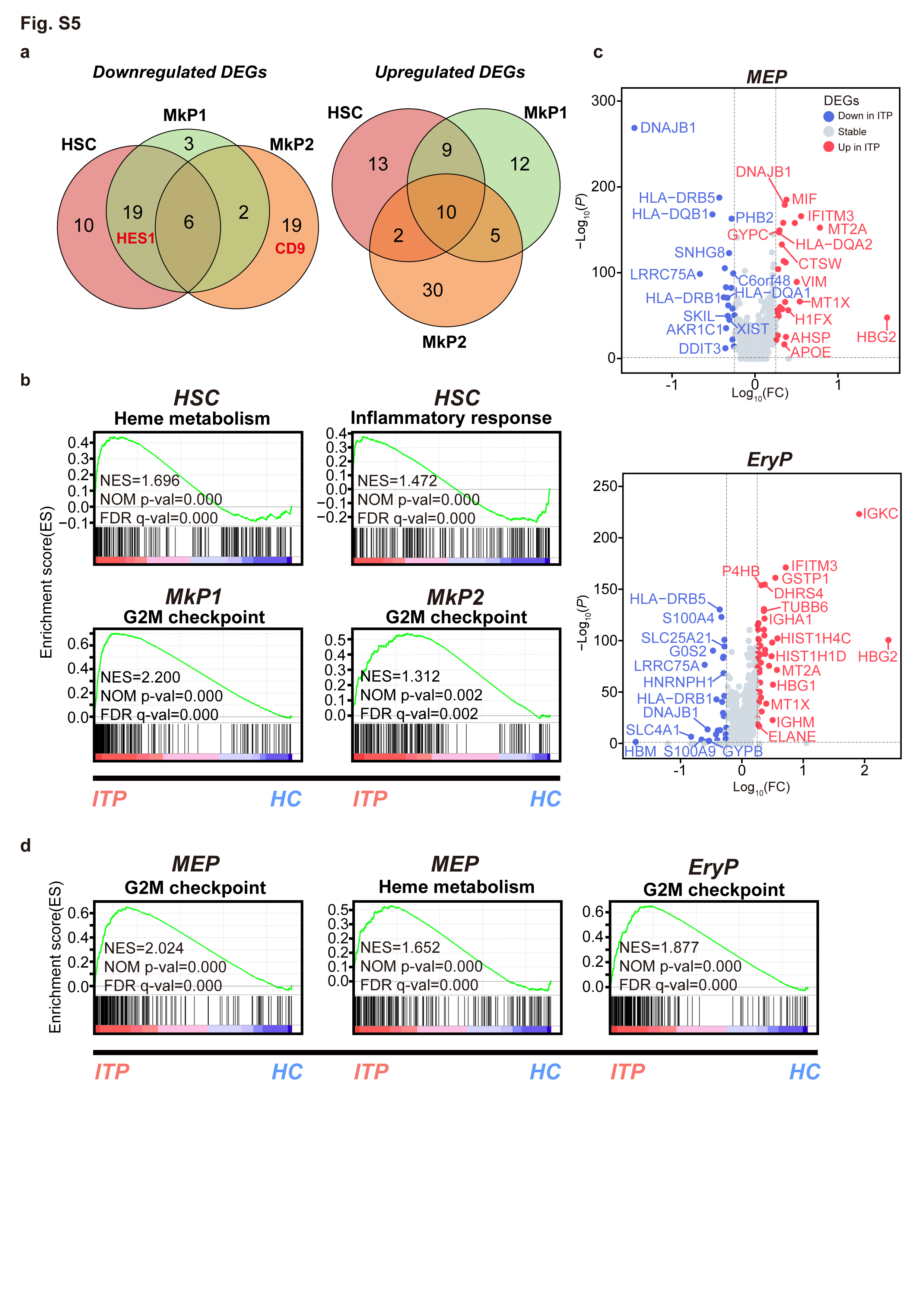

Supplement: Supplementary file 10 — Supplemental Fig. 5 [file 41392_2022_1167_MOESM10_ESM.jpg]

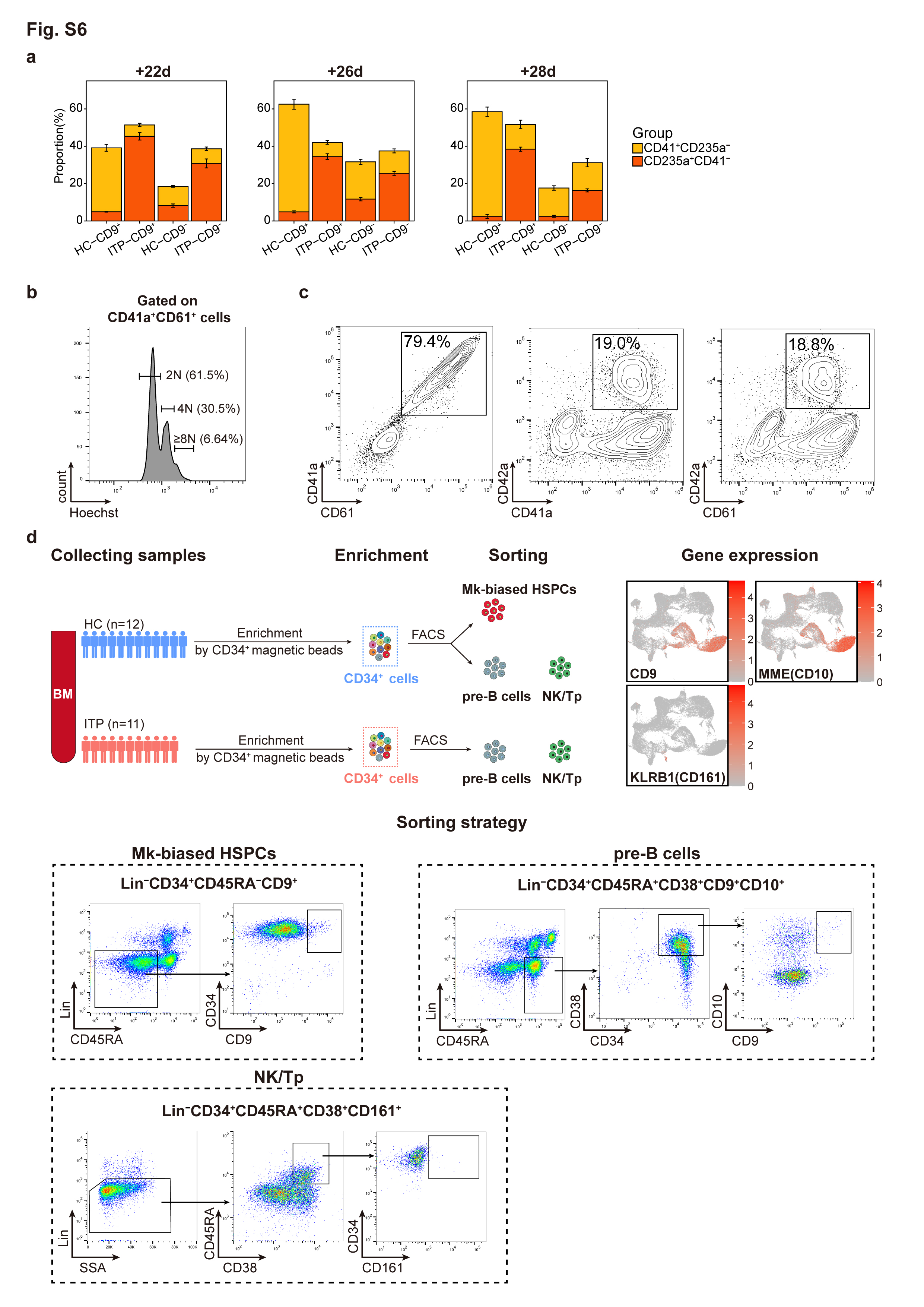

Supplement: Supplementary file 11 — Supplemental Fig. 6 [file 41392_2022_1167_MOESM11_ESM.jpg]

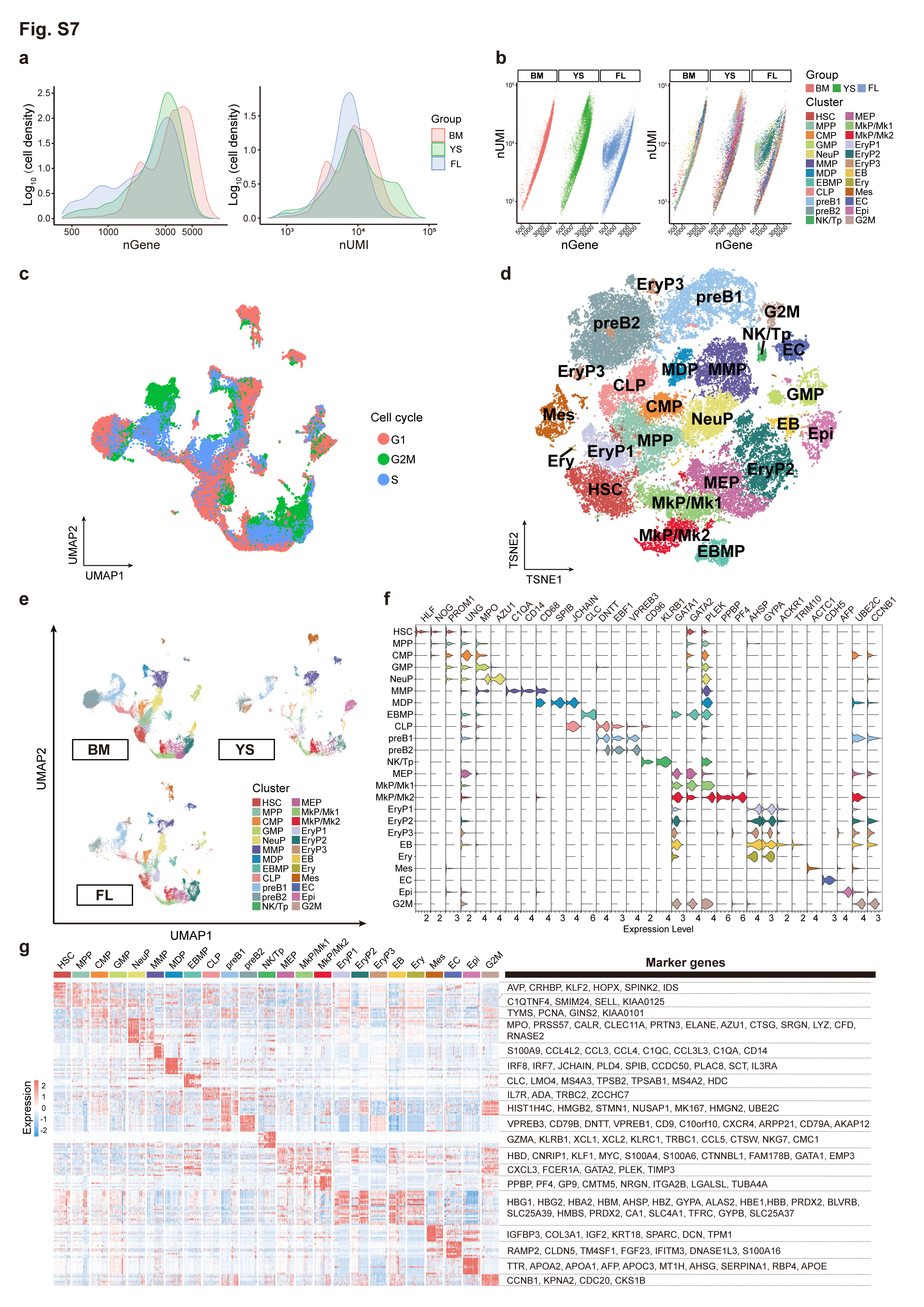

Supplement: Supplementary file 12 — Supplemental Fig. 7–1 [file 41392_2022_1167_MOESM12_ESM.jpg]

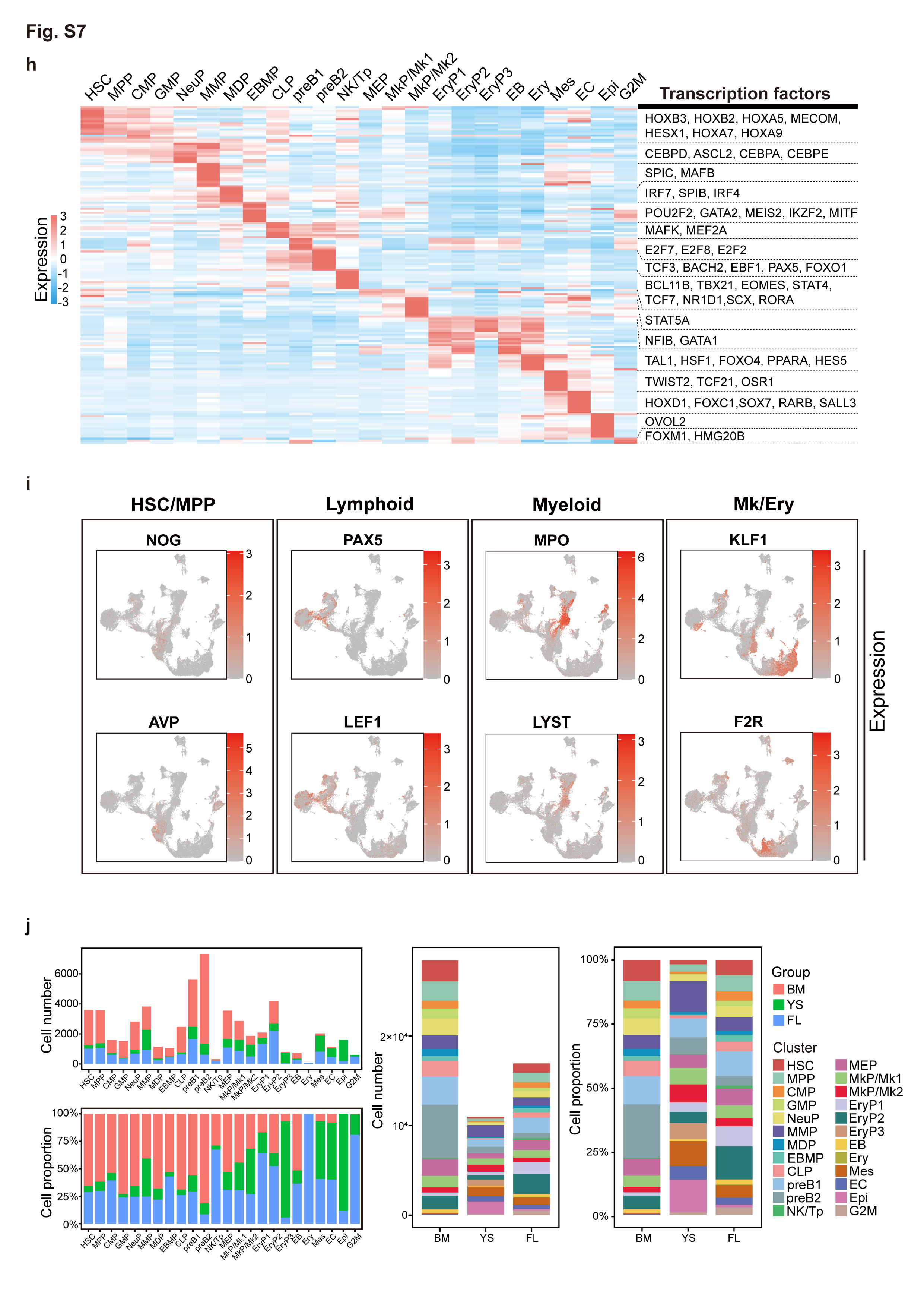

Supplement: Supplementary file 13 — Supplemental Fig. 7–2 [file 41392_2022_1167_MOESM13_ESM.jpg]

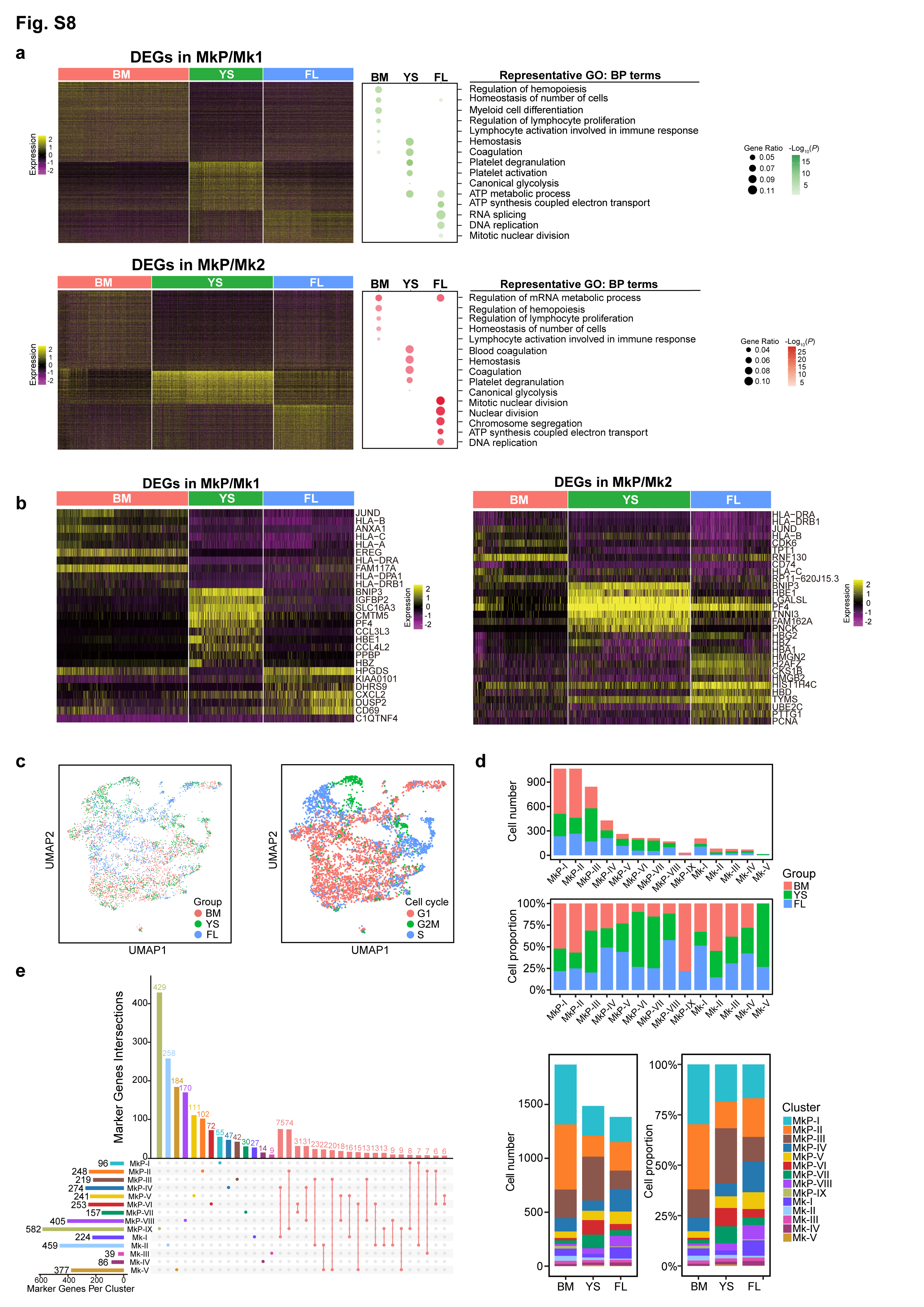

Supplement: Supplementary file 14 — Supplemental Fig. 8–1 [file 41392_2022_1167_MOESM14_ESM.jpg]

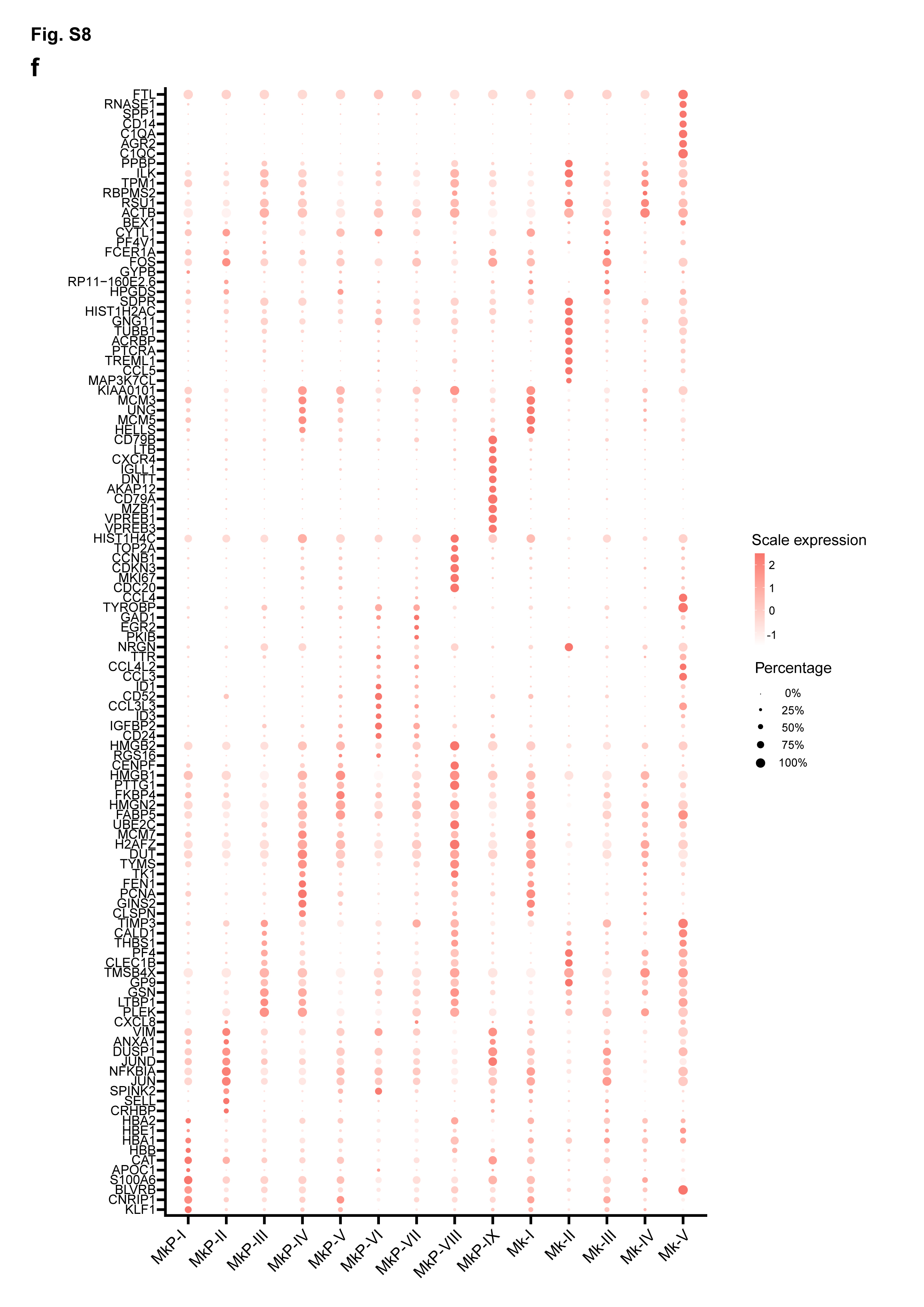

Supplement: Supplementary file 15 — Supplemental Fig. 8–2 [file 41392_2022_1167_MOESM15_ESM.jpg]

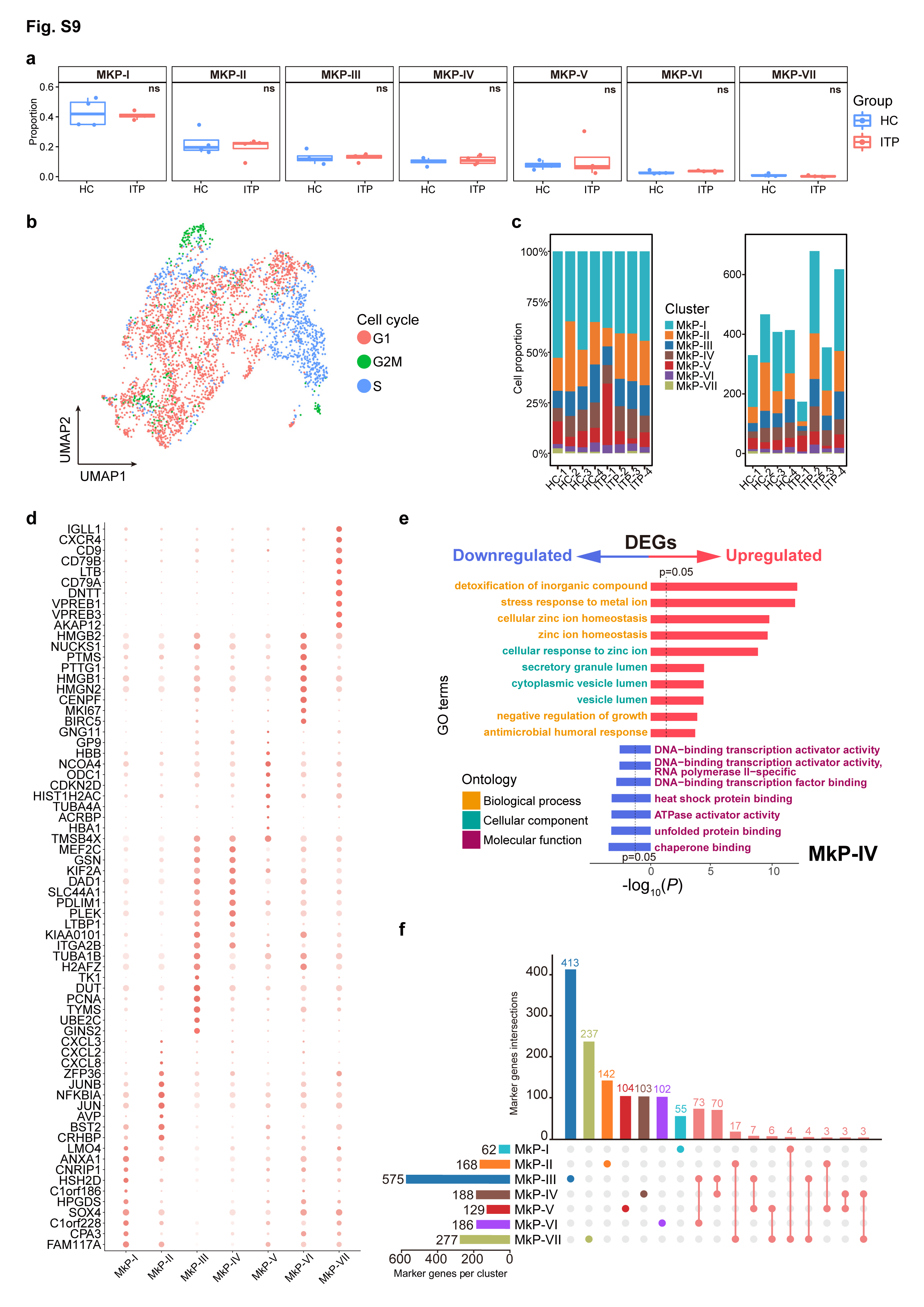

Supplement: Supplementary file 16 — Supplemental Fig. 9 [file 41392_2022_1167_MOESM16_ESM.jpg]

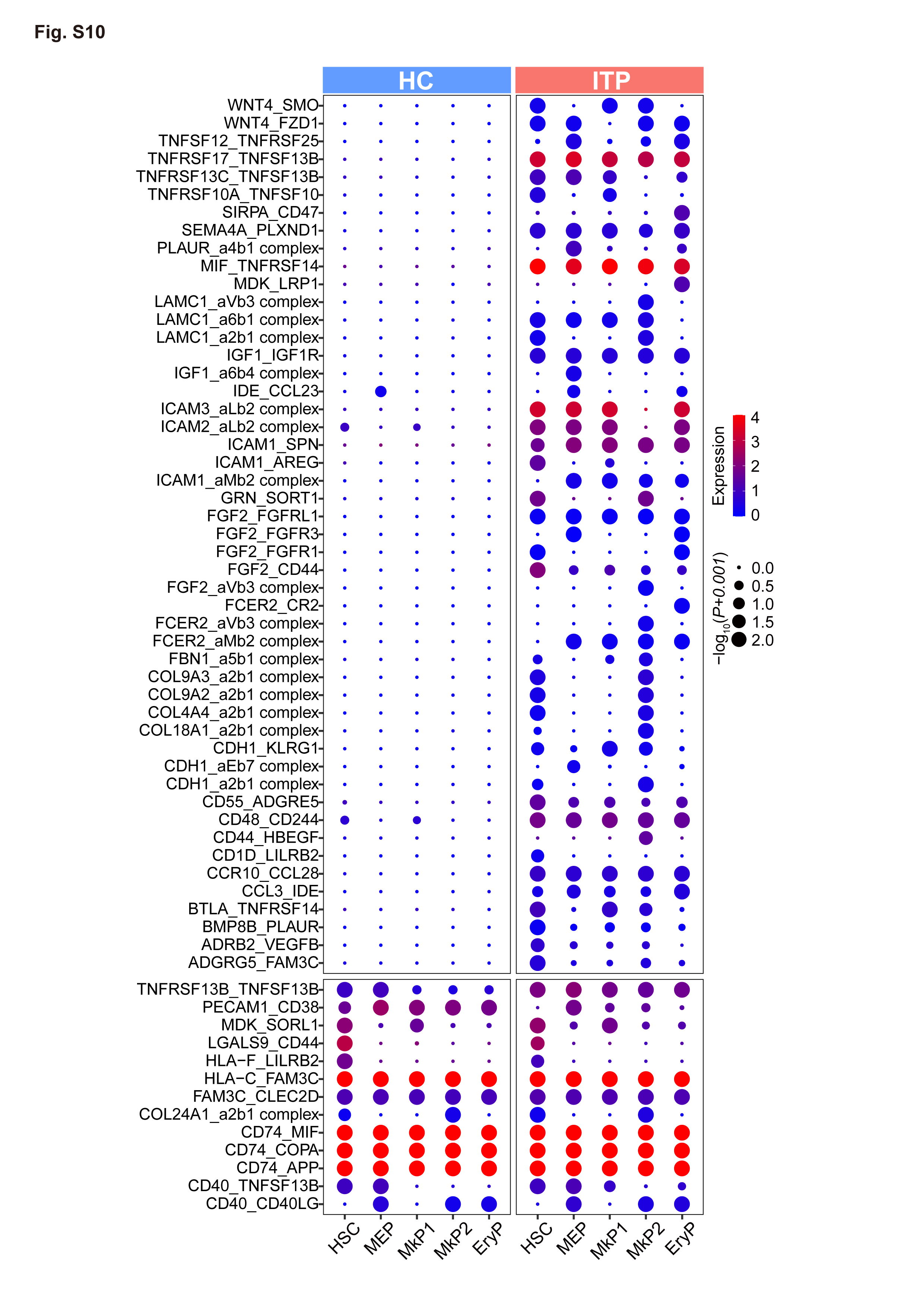

Supplement: Supplementary file 17 — Supplemental Fig. 10 [file 41392_2022_1167_MOESM17_ESM.jpg]

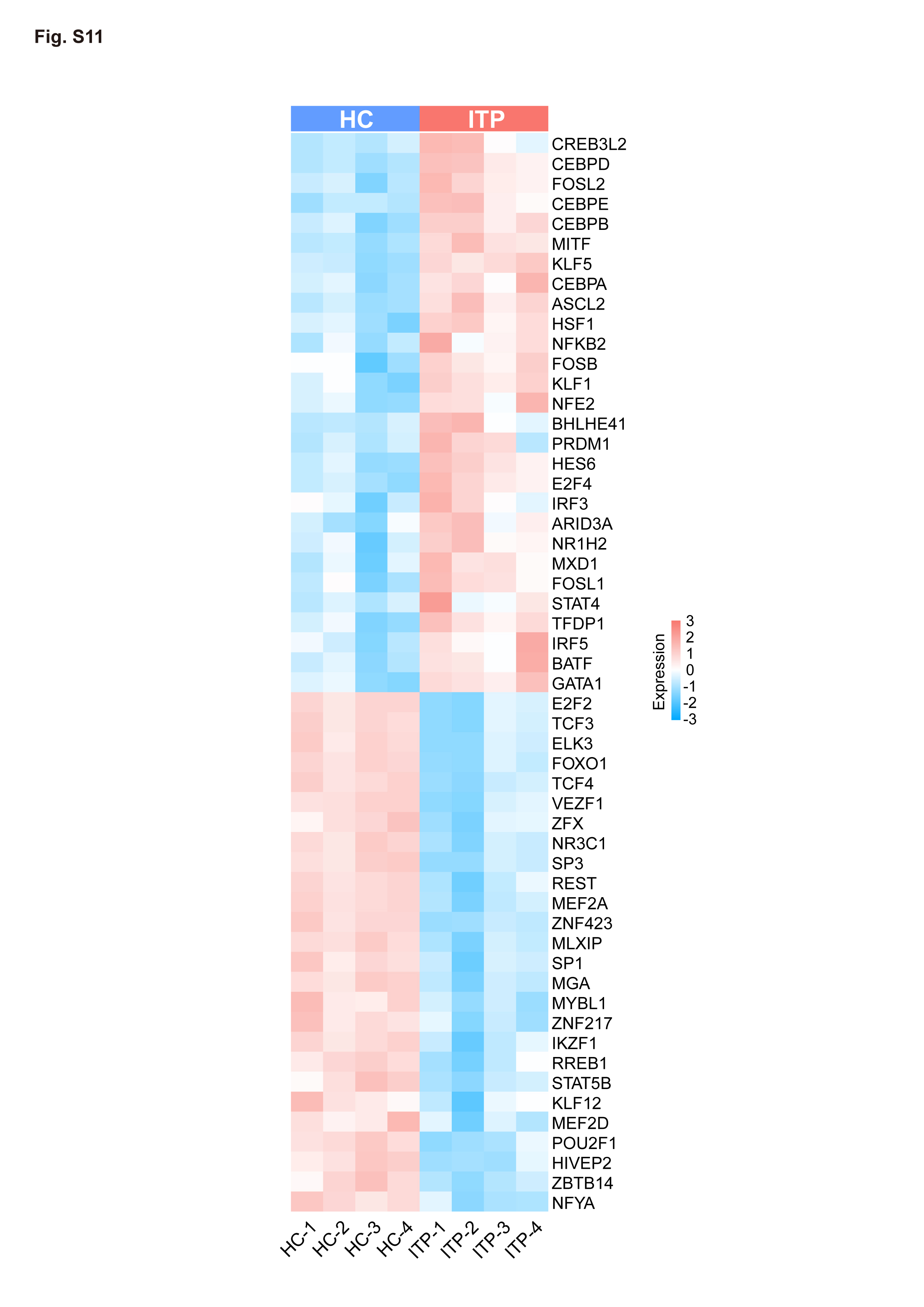

Supplement: Supplementary file 18 — Supplemental Fig. 11 [file 41392_2022_1167_MOESM18_ESM.jpg]

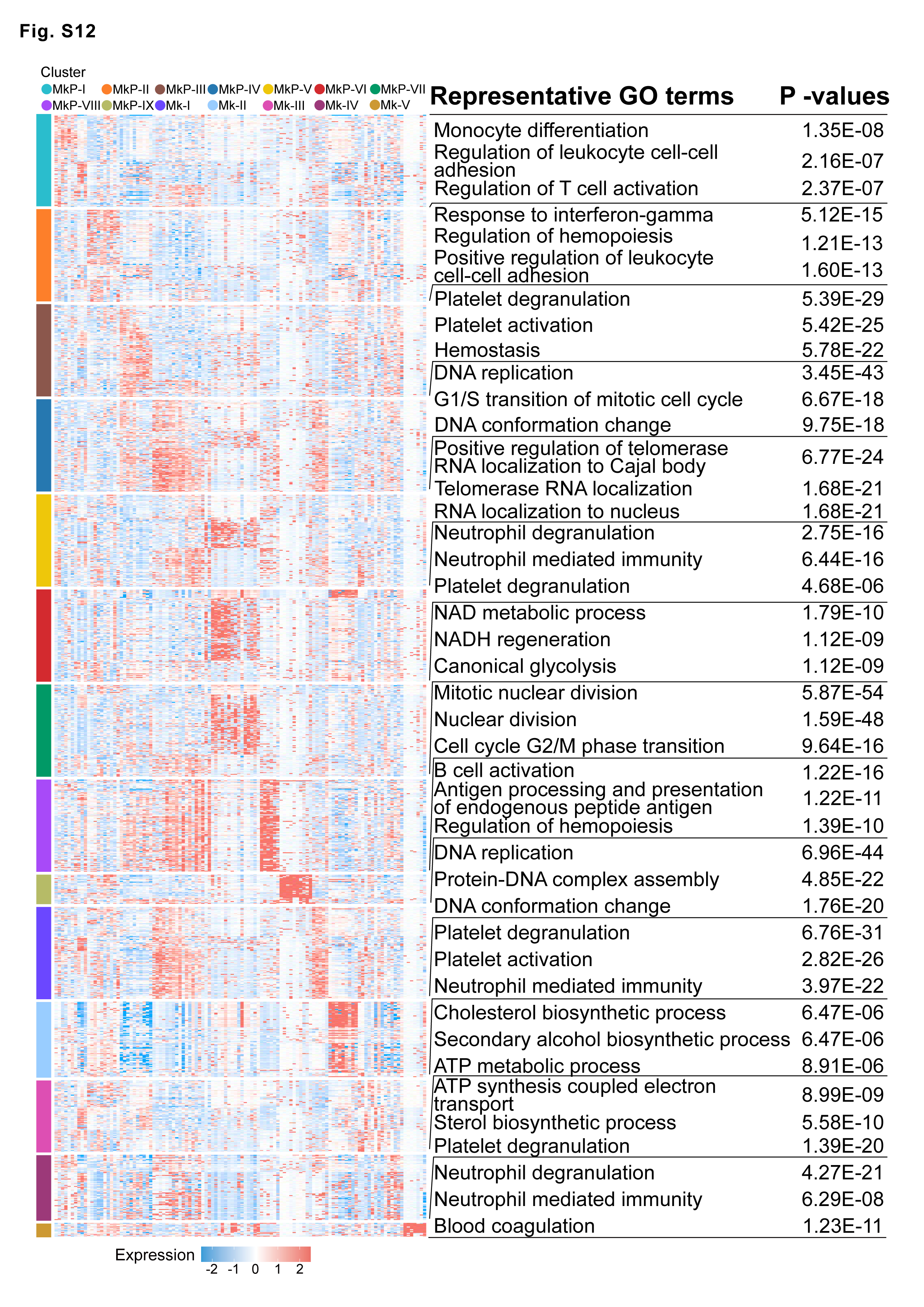

Supplement: Supplementary file 19 — Supplemental Fig. 12 [file 41392_2022_1167_MOESM19_ESM.jpg]

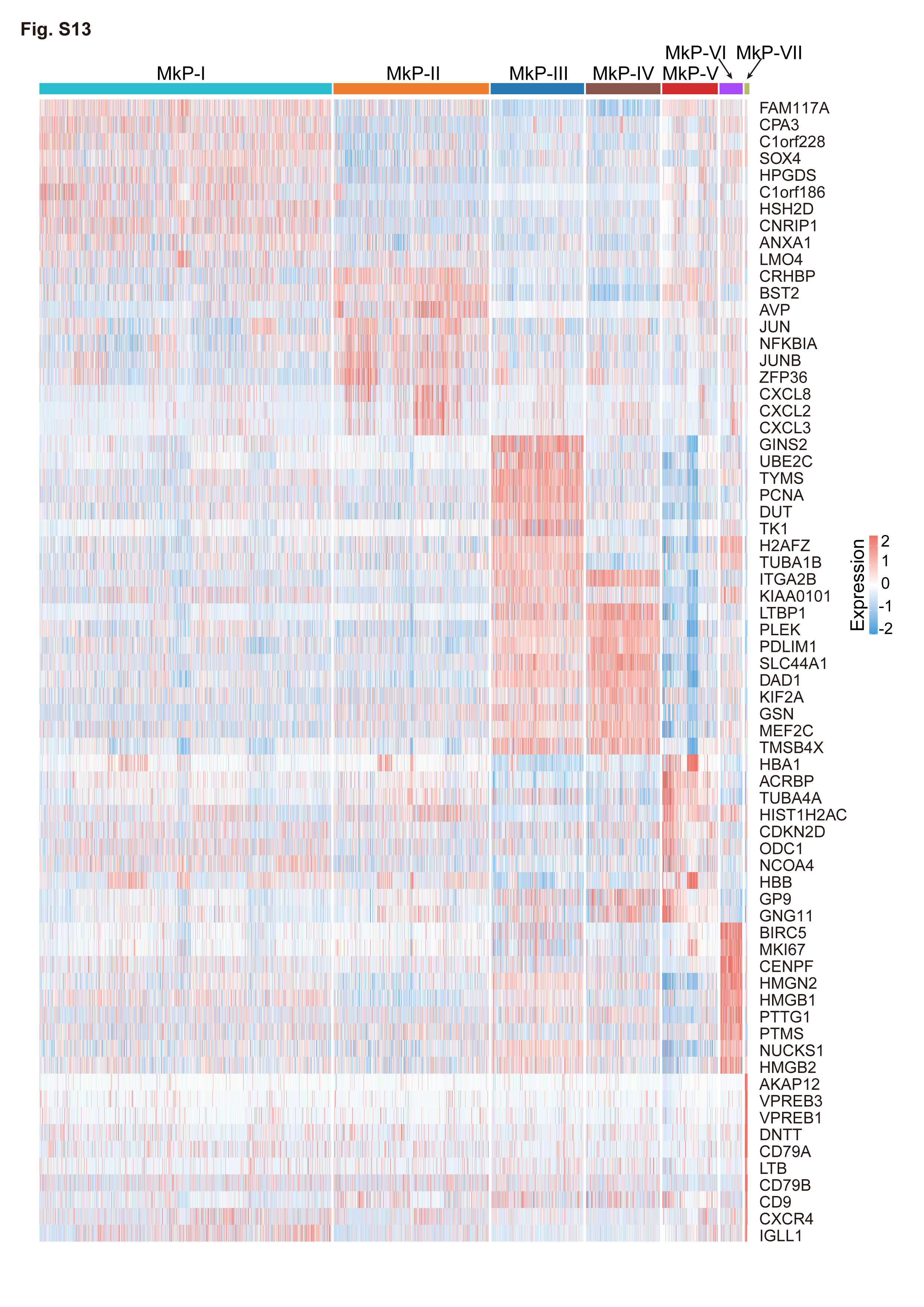

Supplement: Supplementary file 20 — Supplemental Fig. 13 [file 41392_2022_1167_MOESM20_ESM.jpg]

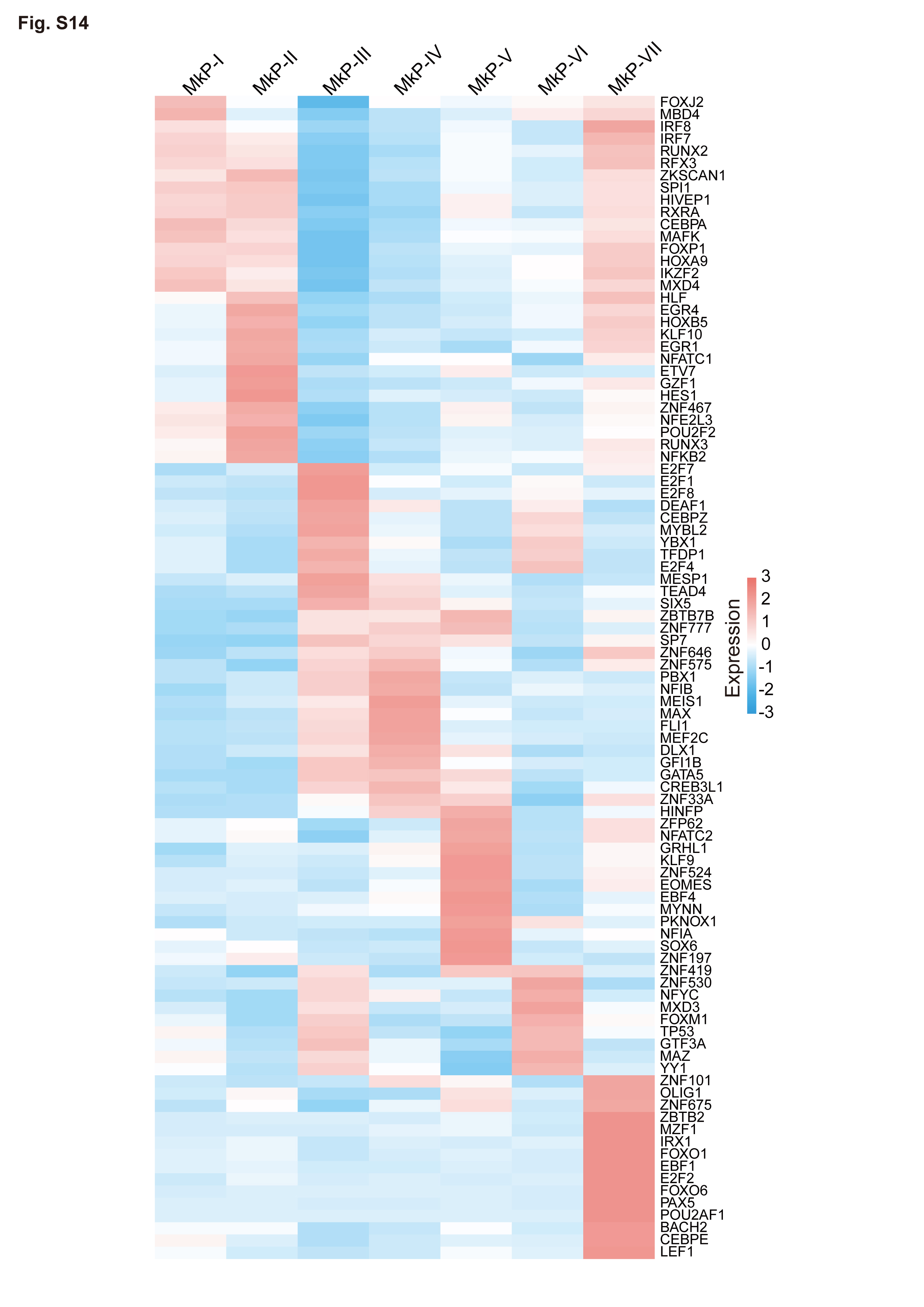

Supplement: Supplementary file 21 — Supplemental Fig. 14 [file 41392_2022_1167_MOESM21_ESM.jpg]
